# Heterogeneous genetic landscape of congenital neutropenia in Korean patients revealed by whole exome sequencing: genetic, phenotypic and histologic correlations

**DOI:** 10.1038/s41598-022-11492-2

**Published:** 2022-05-07

**Authors:** Dajeong Jeong, Sung-Min Kim, Byung Joo Min, Ju Han Kim, Young Seok Ju, Yong-Oon Ahn, Jiwon Yun, Young Eun Lee, Seok Ryun Kwon, Jae Hyeon Park, Jong Hyun Yoon, Dong Soon Lee

**Affiliations:** 1 Department of Laboratory Medicine, Seoul National University Hospital, Seoul National University College of Medicine, 101, Daehak-ro Jongno-gu, Seoul, 110-744 Republic of Korea; 2 Cancer Research Institute, Seoul National University College of Medicine, Seoul, Republic of Korea; 3 Division of Biomedical Informatics, Seoul National University College of Medicine, Seoul, Republic of Korea; 4 Graduate School of Medical Science and Engineering, Korea Advanced Institute of Science and Technology, Daejeon, 34141 Republic of Korea; 5 Department of Laboratory Medicine, Seoul National University Boramae Medical Center, Seoul, Republic of Korea

**Keywords:** Haematological diseases, Medical genetics

## Abstract

Congenital neutropenia (CN) is a hematological disease heterogeneous in its genetic, phenotypic and histologic aspects. We aimed to identify the genetic etiology of Korean CN patients in the context of bone marrow (BM) histology and clinical phenotype. Whole-exome sequencing (WES) or targeted sequencing was performed on the BM or peripheral blood specimens of 16 patients diagnosed with CN based on BM exam from 2009 to 2018. Absolute count of myeloperoxidase (MPO)-positive cells was calculated using ImageJ software. Semi-quantitation of MPO-positive cells in BM sections was performed by MPO grading (grades 0–3). Comprehensive retrospective review on real-world data of 345 pediatric patients with neutropenia including 16 patients in this study during the same period was performed. Seven disease-causing variants were identified in *ELANE*, *G6PC3* and *CXCR4* in 7 patients. A novel homozygous *G6PC3* variant (K72fs) of which the mechanism was copy-neutral loss of heterozygosity was detected in two brothers. A low myeloid-to-erythroid ratio (0.5–1.5) was consistently observed in patients with *ELANE* mutations, while MPO-positive cells (40%–50%) with MPO grade 1 or 2 were detected in myelokathexis caused by *G6PC3* and *CXCR4* mutations. Meanwhile, disease-causing variants were detected in *ELANE*, *TAZ* and *SLC37A4* in 5 patients by retrospective review of medical records. Our results suggest that following the immunological study and BM exam, WES or an expanded next generation sequencing panel that covers genes related to immunodeficiency and other inherited bone marrow failures as well as CN is recommended for neutropenia patient diagnosis.

## Introduction

Congenital neutropenia (CN) is a hematological disease heterogeneous in its phenotypic, histologic and molecular aspects. CN can manifest as isolated neutropenia or neutropenia with extra-hematopoietic abnormalities, immunodeficiency or metabolic diseases. Mutations in more than 20 genes have been demonstrated to cause CN, some of which cause complex phenotypes. Some CN-causing genes show characteristic bone marrow (BM) histologic features such as maturation arrest of granulopoiesis or myelokathexis^1–3^.

Relationship among the clinical phenotype, BM histology and genotype have been documented in many studies. Also, different genes are associated with different inheritance patterns: autosomal dominant (AD), autosomal recessive (AR) or X-linked recessive (XR). For example, CN without extra-hematopoietic abnormalities can result from *ELANE* mutations (AD inheritance), whereas pathogenic variants of *HAX1*, *G6PC3* and *VPS13B* (AR inheritance) can lead to syndromic features affecting multiple organ systems. Variable BM histologic features can be observed in patients carrying each causative gene variant. Maturation arrest at the promyelocyte or myelocyte stage can be caused by *ELANE*, *GFI1* (AD inheritance), *WAS* (XR inheritance), *HAX1* and *G6PC3* mutations^4–10^. Myelokathexis can be observed in patients with *CXCR4* (AD inheritance), *CXCR2* (AR inheritance) or *G6PC3* mutations^11–13^. Mutations in *SBDS*, *EFL1* or *USB1* (AR inheritance) may result in dysplastic hematopoietic cells^3^.

Although clinical features and BM histologies provide some clues to the diagnosis of CN, the same characteristics can be present in other diseases. Some immunodeficiency syndromes, which are often associated with hematologic abnormalities, might be responsible for neutropenia. Mutations in the *ADA2* gene, which encodes one of the two adenosine deaminases, show overlapping features of immunodeficiency and bone marrow failure, both of which contribute to cytopenia^14^.

Differential diagnosis of CN from acquired neutropenia including autoimmune neutropenia is crucial because the clinical and prognostic implications are different: the former is a pre-leukemic disorder with an increased risk of transformation to myelodysplastic syndrome (MDS) or acute myeloid leukemia (AML) or recurrent chronic and life-threatening infections, whereas the latter usually shows a benign clinical course with rare infectious complications^15^. Several algorithms for the diagnosis of CN have been proposed, which commonly include processes for ruling out acquired neutropenia. Thorough investigation of medical history, repeated complete blood count (CBC) with immunological work up such as immunoglobulin (Ig) measurement, anti-neutrophil antibodies and lymphocyte subset analysis followed by a BM exam have been suggested. Then, genetic study was recommended depending on the context^1,3^.

CN patients may benefit from early and timely genetic tests in terms of prognostic perspective. Early hematopoietic stem cell transplantation (HSCT) is known to lower the risk of leukemia development in *ELANE*-mutation patients^16^. It can also reduce the exposure of CN patients to granulocyte-colony stimulating factor (G-CSF) whose cumulative dose is one of the main causes of leukemia development^17^. Early HSCT can reduce the mortality of CN patients by precluding severe infections to which they are predisposed because of low absolute neutrophil count (ANC). Consequently, elucidating the genetic etiology of CN reduces the risk of death and leukemic progression and allows physicians to establish patients’ long-term management plan and familial genetic counseling.

An *ELANE* mutation has been reported to be the most common genetic etiology of CN in Europeans and North Americans^1^. Meanwhile, some CN-causing gene mutations are known to be closely linked to geographic origin. CN caused by a *HAX1* mutation is prevalent in Iran (41%) and Sweden (19%), whereas CN due to mutated *G6PC3* is frequently identified in Israel (19%)^18–20^. In Korea, a few sporadic cases have been reported which include mutations in *ELANE* (NM_001972.4:c.607G > C, p.(G203R) and c.597 + 1G > A) and *CXCR4* (NM_003467.2:c.966_967delAG, p.(G323fs)), as revealed by Sanger sequencing^21–23^. However, no extensive molecular study of Korean CN patients has been conducted so far.

Herein, we aimed to present the comprehensive genetic data of Korean CN patients in the context of clinical phenotype and BM histology. Whole-exome sequencing (WES) or targeted sequencing (TS) was performed on 16 pediatric patients who were diagnosed with CN based on BM exam so that we could detect novel variants and establish the Korean CN molecular data, which is critical for efficient diagnosis of CN.

## Results

### Patients

The median age at the initial presentation of neutropenia was 7.1 months (interquartile range (IQR), 3.8–15.3) and the median age when an initial BM exam was performed was 11.7 months (IQR, 6.8–20.9). The male-to-female ratio was 1:1. Seven (43.8%) patients had a family history of cancer or recurrent infections. Six (37.5%) patients had organomegaly: hepatomegaly in 3, splenomegaly in 1 and hepatosplenomegaly in 2 patients. Fourteen (87.5%) patients experienced infectious complications and half of them suffered from severe infections. Five (31.3%) patients displayed extra-hematopoietic abnormalities in central nervous system, heart and other organs. Permanent and intermittent neutropenia was found in 5 (31.3%) and 11 (68.8%) patients, respectively. Neutropenia was severe in 15 patients (93.8%) and moderate in 1 patient (6.3%). No one exhibited a cyclic pattern of neutropenia. Filgrastim (Grasin; Jeil Pharm, Seoul, Korea) or lenograstim (Neutrogin; Chungai, Seoul, Korea) was administered to 12 (75.0%) patients. Five out of 12 (41.7%) patients responded to G-CSF, while 7 (58.3%) did not. One of the G-CSF responder (P-08) became a G-CSF non-responder later. Five (31.3%) patients underwent allogeneic peripheral blood stem cell transplantation (PBSCT): 4 unrelated PBSCT (uPBSCT) and one haploidentical PBSCT (hPBSCT) from father. Four out of 5 (80.0%) patients had successful PBSCT while one patient (P-05) died despite two successive uPBSCT (Table 1). Alternatively, 3 patients were observed without any treatment. No patients evolved to MDS or AML during the follow-up period (median, 48.6 months; IQR, 20.9–72.7).

**Table 1:** Clinical, histologic, cytogenetic and molecular characteristics of 16 neutropenia patients.

P	Age^*^	Hb (g/l)	WBC (× 10^9^/l)	PLT (× 10^9^/l)	ANC (× 10^9^/l)	Neutropenia	Recurrent infection	Extra-hematopoietic features	G-CSF	G-CSF response	PBSCT	IgG/A/M (mg/dl)	Maturation arrest^†^	Myelo-kathexis^†^	MPO (%)^‡^	MPO grade^‡^	Likely pathogenic or pathogenic Variants^§^
01	16	13.7	7000	305	146	Intermittent	Yes	None	Grasin 75mcg × 9 for 1341 days	No	No	N/A	Band stage	None	30.1	1	None
02	18	11.9	11,530	142	189	Intermittent	No	None	Grasin 75mcg × 2 for 3 days	Yes	No	N/A	Band stage	None	46.2	2	None
03	0.5	8.3	10,340	565	0	Permanent	Yes	High arched palate	Grasin 75mcg × 13 for 24 days	No	No	1570/85/215	Myelocyte stage	None	N/A	N/A	*ELANE* (NM_001972.2):c.452G > A, p.(C151Y), heterozygous
04	8	10.1	9190	457	254	Intermittent	Yes	None	Grasin 75mcg × 11 for 428 days	Yes	No	N/A	Band stage	None	51.6	3	None
05	3	12.4	5590	244	102	Permanent	Yes	None	N/A	N/A	uPBSCT x2^||^	1074/183/62	N/A	N/A	12.6	0	*ELANE* (NM_001972.2):c.640G > A, p.(G214R), heterozygous
06	18	11.2	2300	140	125	Intermittent	Yes	None	Neutrogin 50mcg × 2 for 2 days	No	No	N/A	None	None	38.5	3	None
07	10	11.3	5350	382	0	Intermittent	Yes	None	N/A	N/A	No	N/A	Band stage	None	47.7	3	None
08	4	13.0	2950	280	0	Permanent	Yes	Incomplete cleft lip, inguinal hernia	Grasin 75mcg × 13 for 410 days	Yes^¶^	uPBSCT	282/19/59	None	Yes	44.4	2	*CXCR4* (NM_003467.2)c.978_979del, p.(G323fs), heterozygous
09	14	10.9	4110	252	0	Intermittent	Yes	None	Grasin 75mcg × 4 for 302 days	Yes	No	N/A	Band stage	None	58.9	3	None
10	4	10.8	7460	364	64	Intermittent	Yes	Lipomeningomyelocele, pes calcaneous	Grasin 75mcg × 2 for 11 days	Yes	No	N/A	None	None	20.1	0	None
11	0.3	10.0	8660	153	0	Permanent	Yes	None	Grasin 75mcg × 88 for 137 days	No	hPBSCT × 2	1023/71/52	Promyelocyte stage	None	45.5	3	*ELANE* ((NM_001972.2):c.640G > A, p.(G214R), heterozygous
12	10	11.2	5180	227	825	Intermittent	Yes	None	N/A	N/A	No	N/A	None	None	39.4	3	None
13	28	11.5	1430	204	1301	Intermittent	Yes	ASD, Crohn's disease, growth retardation, JRA, PTC, nephrocalcinosis	Neutrogin 150mcg × 7 for 11 days Grasin 150mcg × 316 for 3180 days	No	No	2253/231/143	None	Yes	46.5	1	*G6PC3* (NM_138387.3):c.214delA, p.(K72fs), homozygous
14	48	11.3	3320	155	958	Intermittent	Yes	ASD, Crohn's disease, growth retardation, prominent skin vessels, testicular microlithiasis, clinodactyly of both 5th fingers	Neutrogin 100mcg × 14 for 75 days Grasin 150mcg × 316 for 3095 days	No	uPBSCT	1362/139/59	None	Yes	40.4	1	*G6PC3* (NM_138387.3):c.214delA, p.(K72fs), homozygous
15	1	10.5	10,090	292	796	Permanent	Yes	None	Grasin 75mcg × 19 for 115 days	No	uPBSCT	1283/89/89	Promyelocyte stage	None	25.2	1	*ELANE* (NM_001972.2:c.608G > A, p.(G203D), heterozygous
16	3	6.7	5480	40	1064	Intermittent	No	None	N/A	N/A	No	N/A	None	None	N/A	N/A	None

^*^Age at initial presentation of neutropenia.

^†^One patient with diluted BM aspiration in which reliable differential count could not be made was excluded.

^‡^Two patients were not included due to inadequate BM section quality or retrospectively unavailable BM paraffin block for MPO stain.

^§^Only likely pathogenic or pathogenic variants are documented in this table. Information on all the variants detected including VUS is displayed in Table S3.

^||^One out of 5 (20.0%) who underwent PBSCT died despite two successive uPBSCT.

^¶^One patient (P-08) initially responded to G-CSF but then became a non-responder.

^#^Haploidentical PBSCT from father.

*ANC* absolute neutrophil count, *ASD* atrial septal defect, *BM* bone marrow, *G-CSF* granulocyte-colony stimulating factor, *Hb* haemoglobin, *hPBSCT* haploidentical peripheral blood stem cell transplantation, *JRA* juvenile rheumatoid arthritis, *M:E* myeloid to erythroid, *MPO* myeloperoxidase, *N/A* not applicable, *P* patient, *PBSCT* peripheral blood stem cell transplantation, *PLT* platelet, *PTC* papillary thyroid cancer, *uPBSCT* unrelated peripheral blood stem cell transplantation, *WBC* white blood cell.

### Laboratory results

Median CBC values were as follows: hemoglobin 11.2 g/l (IQR, 10.2–11.8), white blood cell count 5.54 × 10^9^/l (IQR, 3.52–9.06) and platelet level 248 × 10^9^/l (IQR, 154–349). Median ANC was 0.07 × 10^9^/l (IQR, 0.00–0.31), while median absolute monocyte count was 0.56 × 10^9^/l (IQR, 0.33–0.89). Median absolute lymphocyte count was 4.76 × 10^9^/l (IQR, 2.66–6.83) and median absolute eosinophil count was 0.16 × 10^9^/l (IQR, 0.09–0.45). Monocytosis was observed in 2 patients (P-03 and P-11), while eosinophilia was not detected based on the reference range for each patient’s age.

Ig levels were retrospectively available only in 7 (43.8%) patients. Median IgG and IgM levels were within the reference range (IgG, 1362 mg/dl, IQR 1179–1797; IgM, 62 mg/dl, IQR 59–116), while IgA was slightly reduced (median 89 mg/dl, IQR 78–161). One patient (P-08) showed decreased levels of both IgG and IgA while two patients (P-11 and P-15) had slightly decreased level of IgA compared to reference range (Table 1).

### Bone marrow histology

BM of the 16 patients had a median myeloid-to-erythroid (M:E) ratio of 1.9 (IQR, 1.2–3.7) and cellularity of 85% (IQR, 75–90). The BM aspirate of one patient (P-08) revealed dysplastic neutrophils with pyknotic lobes with long filaments and hypolobation. Maturation arrest of granulopoiesis was detected in 8 out of 15 (53.3%) patients whose BM aspirates were of good quality for the assessment. Three (20.0%) of the 15 patients presented maturation arrest at the promyelocyte or myelocyte stage, while in 5 (33.3%) patients maturation block was observed at the band stage. Myelokathexis was noted in 3 (20.0%) of the 15 patients. No BM fibrosis or histiocytes with hemophagocytic activity were observed in any of the patients.

Median percentage of Myeloperoxidase (MPO)-positive cells in the 14 patients for whom BM biopsy specimens of adequate quality were available was 42.4% (IQR, 32.2–46.5). The MPO grade was as follows: grade 0 (n = 2, 14.3%), grade 1 (n = 4, 28.6%), grade 2 (n = 2, 14.3%) and grade 3 (n = 6, 42.9%). There was a tendency toward a positive correlation between the MPO grade and the percentage of MPO-positive cells, though it was not statistically significant (*P* = 0.086). No distinct association between the MPO grade and M:E ratio was observed (*P* = 0.477) (Supplementary Fig. S1).

### Cytogenetics

Most patients (n = 15, 93.8%) had a normal karyotype, whereas one patient (P-16) carried 46,XX,add(14)(p13).

### Results of whole-exome sequencing and targeted sequencing

A total of 102 variants in genes related to CN, inherited bone marrow failure (IBMF), or immunodeficiency were detected in the 16 patients. One benign, 64 likely benign, 29 variants of unknown significance (VUS), 2 likely pathogenic and 6 pathogenic variants were identified. Pathogenic variants in CN-related genes included *ELANE* (NM_001972.2):c.640G > A, p.(G214R) in 2 patients, *G6PC3* (NM_138387.3):c.214delA, p.(K72fs) in 2 brothers, which was a novel variant, and *CXCR4* (NM_003467.2):c.966_967del, p.(R326fs) in 1 patient, which was previously reported^23^. A heterozygous pathogenic variant of *FANCI* (NM_001113378.1:c.3172G > T, p.(E1058X)) was identified in one patient. Likely pathogenic variants in *ELANE* (NM_001972.2: c.452G > A, p.(C151Y) and c.608G > A, p.(G203D)) were found in 2 patients. Twenty-nine VUS were as follows: *SEPTIN6* (NM_015129.5:c.1085A > G, p.(K362R)).

*LRBA* (NM_006726.3:c.3778G > C, p.(A1260P) and c.1408A > T, p.(I470F)) in 2 patients, *IL12RB1* (NM_005535.1:c.1601C > T, p.(P534L)), *SLC7A7* (NM_001126106.1:c.333 T > G, p.(F111L)) in 2 patients, *C6* (NM_000065.2:c.449G > A, p.(R150H)), *CFP* (NM_002621.2:c.1366C > G, p.(L456V)), *TCF3* (NM_003200.2:c.1069G > A, p.(V357M)), *LYST* (NM_000081.2:c.5480G > A, p.(C1827Y)), *SMARCAL1* (NM_014140.3:c.1786G > A, p.(A596T)), *LIG1* (NM_000234.1:c.1879C > T, p.(R627W)), *MAGT1* (NM_032121.5:c.572G > A, p.(R191Q)), *NCF4* (NM_013416.3:c.457C > T, p.(R153C)), *RNASEH2C* (NM_032193.3:c.270G > C, p.(K90N)), *DNASE2* (NM_001375.2:c.319G > A, p.(D107N)), *DOCK2* (NM_004946.2:c.5017C > T, p.(P1673S)), *EPG5* (NM_020964.2:c.7736G > A, p.(R2579Q)), *FANCG* (NM_004629.1:c.70G > A, p.(V24I)), *SPINK5* (NM_006846.3:c.775G > C, p.(A259P)), *IRAK1* (NM_001569.3:c.609 T > G, p.(C203W)), *TGFBR2* (NM_003242.5:c.1013C > T, p.(T338M)), *NFE2L2* (NM_006164.3:c.76C > T, p.(R42X)), *CD3G* (NM_000073.2:c.56-1G > A), *CD79B* (NM_000626.2:c.97G > C, p.(E33Q)), *NLRP3* (NM_004895.4:c.200C > G, p.(A67G)), *NSMCE3* (NM_138704.4:c.342C > A, p.(H114Q)) and *SKIV2L* (NM_006929.5:c.151G > A, p.(A51T)).

Meanwhile, 8 cancer predisposition gene mutations which were assessed as VUS were detected in 4 patients: *ANKRD26* (NM_014915.2:c.3086A > T, p.(E1028V)), *BARD1* (NM_000465.2:c.1862 T > C, p.(M151T)), *DHX34* (NM_014681.5:c.1831G > A, p.(A611T)), *ERCC6* (NM_000124.2:c.2996A > G, p.(A2996G)), *FH* (NM_000143.3:c.1434T > A, p.(N478K)), *KDM1A* (NM_001009999.2:c.44C > T, p.(A15V)), *MST1R* (NM_002447.2:c.1729delC, p.(H577fs)) and *SMARCA4* (NM_001128849.1:c.4925A > C, p.(K1580T)) (Table 1 and Supplementary Table S1).

### Copy-number variant analysis

A total of 31 Copy-number variants (CNV) in 11 patients were assessed as VUS, none of which involved regions including genes associated with CN, IBMF or immunodeficiency. No pathogenic CNVs were identified (Supplementary Table S2). Copy-neutral loss of heterozygosity (CN-LOH) at 17q21.31 was revealed in 2 brothers (P-13 and P-14) with pathogenic homozygous mutations in *G6PC3* (NM_138387.3:c.214delA, c.214delA, p.K72fs) (Supplementary Fig. S2).

### Sixteen neutropenia patients: clinical, histologic and molecular features

In the 16 neutropenia patients, an average of 2.4 variants, including VUS, per patient were detected in genes related to CN, IBMF or immunodeficiency. An average of 2.9 variants, including VUS, per patient were identified in genes related to CN, IBMF, immunodeficiency or cancer predisposition. Detailed information on the clinical features of the neutropenia, recurrent infections, extra-hematopoietic features, G-CSF doses, G-CSF response, PBSCT, Ig levels, BM histology of maturation arrest, myelokathexis, MPO-positive cell percentage and MPO grade, and variants detected by WES or TS in each patient is described in Table 1 and Supplementary Table S1.

### Congenital neutropenia with causal pathogenic variants

The 4 patients who harbored likely pathogenic or pathogenic *ELANE* mutations showed common features of permanent neutropenia, recurrent infections with several neutropenic fever events, G-CSF non-responsiveness and maturation arrest at the promyelocyte or myelocyte stage with no dysplastic hematopoietic cells. Three out of 4 (75.0%) patients had organomegaly: 2 had hepatosplenomegaly and 1 had splenomegaly. One patient had an extra-hematopoietic feature of a high arched palate and family history of liver and gastric cancer in his maternal grandfather. Monocytosis was observed in 2 (50.0%) patients. The 4 patients displayed a variable percentage of MPO-positive cells (range, 12.6%–45.5%) and MPO grade (range, 0–3). Two patients (P-05 and P-11) harbored the same pathogenic *ELANE* mutation (NM_001972.2:c.640G > A, p.(G214R)) but their BM histology and clinical outcomes were totally different: BM biopsy revealed 12.6% of MPO-positive cells and MPO grade 0 in P-05 but 45.5% of MPO-positive cells and MPO grade 3 in P-11 (Supplementary Fig. S3). The former underwent two uPBSCT, but he died of sepsis. The latter patient had successful second hPBSCT, which led to the correction of the ANC level. (Supplementary Fig. S4).

The patient (P-08) with a pathogenic *CXCR4* mutation (NM_003467.2:c.966_967del, p.(R326fs)) showed permanent neutropenia, several neutropenic fever events with or without infections, inguinal hernia, incomplete cleft lip and serum IgG and IgA deficiencies. Myelokathexis was the most representative histologic feature with increased M:E ratio of 7.9, MPO grade 2 and 44.4% of MPO-positive cells on a BM section (Supplementary Fig. S5). An ANC of > 1.00 × 10^9^/l has been maintained after he had uPBSCT (Supplementary Fig. S6).

**Figure 1:**
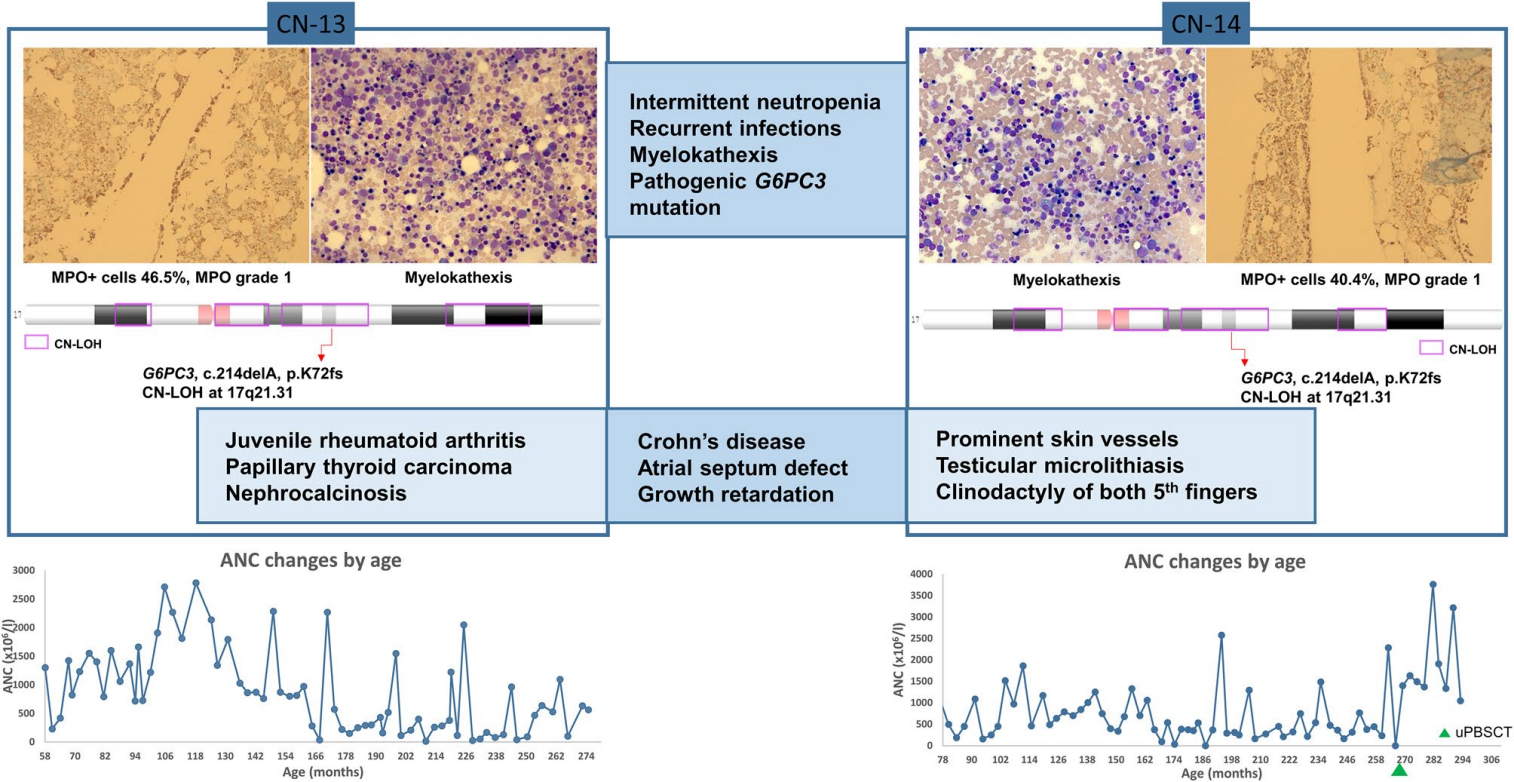
Overlapping clinical, histological and genetic characteristics of the two brothers with the *G6PC3* mutation. Two brothers (P-13 and P-14) with the same pathogenic homozygous *G6PC3* mutation (NM_138387.3:c.214delA, p.(K72fs)), which was caused by a copy-neutral loss of heterozygosity at 17q21.31, showed overlapping clinical manifestations and BM histologic features. Both of them presented intermittent neutropenia and experienced mild to severe infections. They had similar extra-hematopoietic features such as an atrial septum defect, Crohn’s disease and growth retardation. Meanwhile, some features were different: P-13 suffered from juvenile rheumatoid arthritis, papillary thyroid carcinoma and medullary nephrocalcinosis with multiple cysts, whereas P-14 showed prominent skin vessels, bilateral testicular microlithiasis, necrotizing enterocolitis and clinodactyly of both 5th fingers. Myelokathexis with markedly increased segmented neutrophils was noted with MPO grade 1 and 40.4% (P-13) and 46.5% (P-14) of MPO-positive cells. The graphs at the bottom show absolute neutrophil count changes by age, which show a similar pattern of intermittent neutropenia in both patients.

Clinical manifestations and BM histologies were very similar in the P-13 and P-14 brothers. Both presented intermittent neutropenia and suffered from numerous infectious events from mild to severe with neutropenic or non-neutropenic fever (Supplementary Fig. S7). They showed similar extra-hematological involvement such as an atrial septum defect, Crohn’s disease and growth retardation. Meanwhile, some different features were noted: P-13 suffered from juvenile rheumatoid arthritis, papillary thyroid carcinoma and medullary nephrocalcinosis with multiple cysts, whereas P-14 showed prominent skin vessels, bilateral testicular microlithiasis, necrotizing enterocolitis and clinodactyly of both 5th fingers. Both were G-CSF non-responders. Myelokathexis with prominently increased hypermature segmented neutrophils was observed with MPO grade 1 and 40.4%–46.5% MPO-positive cells on BM sections (Fig. 1). In contrast to BM of the patient with the *CXCR4* (R326fs) mutation, who exhibited myelokathexis with an overall increase in the number of myeloid cells at each stage, BM of patients with the *G6PC3* (K72fs) mutation displayed a prominent increase in the number of segmented neutrophils but not of the other kinds of myeloid cells (Supplementary Fig. S5).

### Chronic idiopathic neutropenia

One patient (P-01) with a variant of unknown significance in *SEPTIN6* (NM_015129.5:c.1085A > G, p.(K362R)) had intermittent neutropenia for more than 3 years. She experienced non-severe infections such as upper respiratory infection, acute pharyngotonsillitis and acute otitis media with neutropenic or non-neutropenic fever. She was a G-CSF non-responder. A BM exam revealed maturation arrest at the band stage, 30.1% of MPO-positive cells, MPO grade 1 and no dysplastic hematopoietic cells (Supplementary Fig. S8).

### Acquired neutropenia

No pathogenic mutations or candidate variants suspected as the cause of neutropenia were identified in 8 out of 16 (50.0%) patients. In these 8 patients, median neutropenia duration was 18.7 months. They showed intermittent neutropenia. Five out of 8 received G-CSF treatment and one of them was a G-CSF non-responder (P-06). Two out of the 8 (25.0%) patients (P-02 and P-16) experienced no infections or fever and both had hepatomegaly. Meanwhile, 2 (25.0%) patients suffered from neutropenic fever with non-severe infection, whereas 4 (50.0%) patients experienced neutropenic or non-neutropenic fever with non-severe infection. Four (50.0%) patients showed maturation block at the band stage, whereas 4 (50.0%) patients showed no maturation arrest. One of the 7 patients (P-02) had a history of immune thrombocytopenia and received intravenous immunoglobulin (IVIG) treatment. Fluorescent antinuclear antibody test revealed weak positivity. Another patient (P-07) had a history of Kawasaki disease and received IVIG treatment. She had an older brother who suffered from recurrent pneumonia in childhood but became healthy later. Meanwhile, there was a patient (P-16) who had the constitutional karyotype of 46,XX,add(14)(p13), the significance of which is not clear.

### Re-diagnosis of 16 neutropenia patients based on genetic analysis

**Figure 2:**
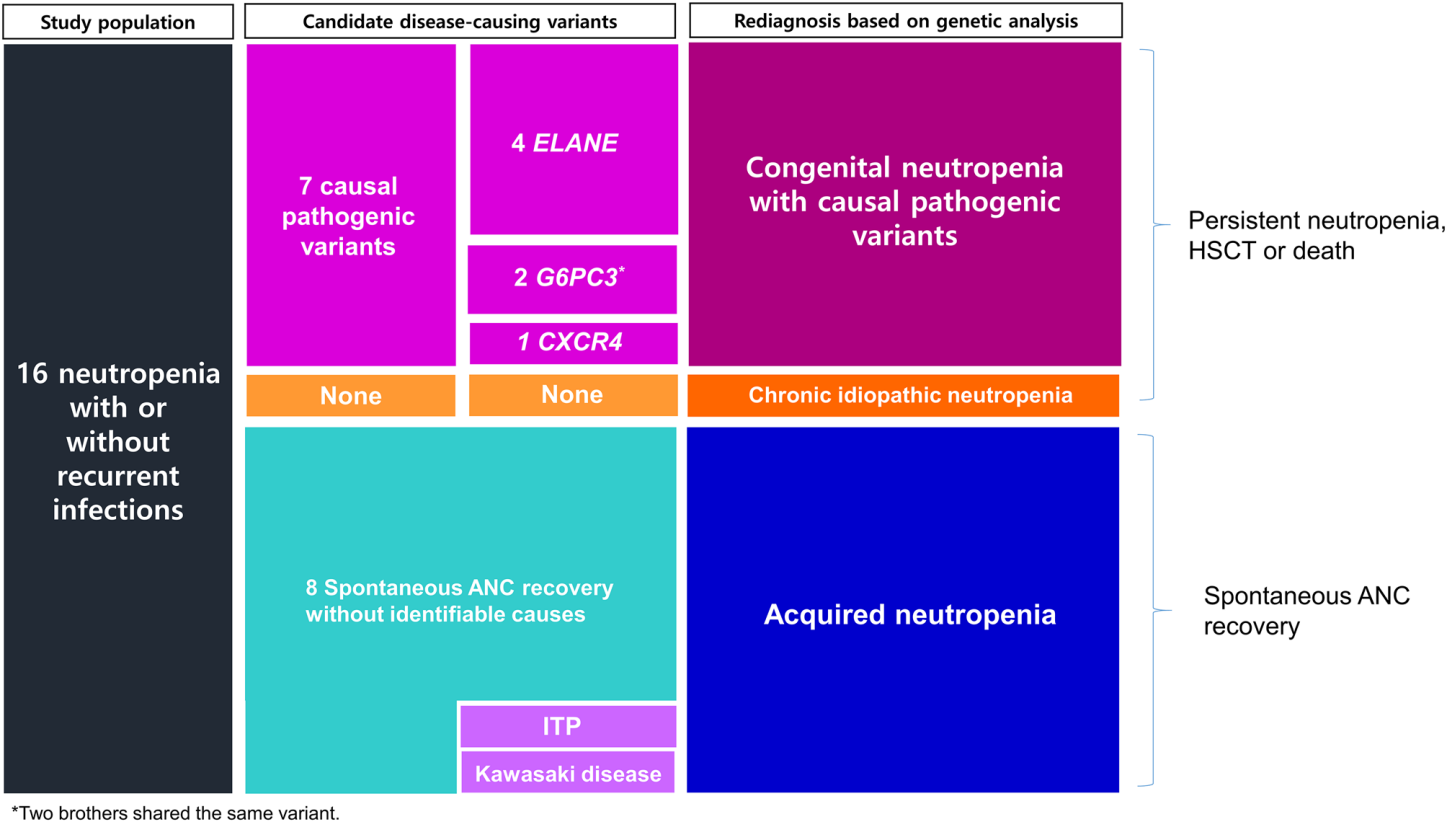
Re-diagnosis of 16 neutropenia patients based on genetic analysis. Seven patients with pathogenic variants in *ELANE*, *G6PC3* and *CXCR4* were diagnosed with CN with causal pathogenic variants. One patient harbored no pathogenic variant and was classified as chronic idiopathic neutropenia. Other 8 patients who achieved spontaneous ANC recovery were re-diagnosed with acquired neutropenia. Two out of the 5 patients had a history of immune thrombocytopenia and Kawasaki disease, respectively, and both were treated with IVIG. ITP, immune thrombocytopenic purpura.

The causal pathogenic variants were detected in 7 patients and included 4 mutations in *ELANE*, 2 in *G6PC3* and 1 in *CXCR4*. They were re-diagnosed with CN with causal pathogenic variants. A patient with intermittent neutropenia for more than 3 years without identifiable causes was classified into chronic idiopathic neutropenia. The 8 remaining patients were re-diagnosed as having acquired neutropenia (Fig. 2).

### Genotype–BM histology correlations

Based on the genotyping results, we examined the relationship between genotype and BM histology, mainly focusing on maturation arrest and myelokathexis. Early maturation arrest at the promyelocyte or myelocyte stage was characteristic for *ELANE* mutations. The *CXCR4* mutation was associated with myelokathexis with MPO grade 2 and *G6PC3* mutations with BM retention with MPO grade 1. Maturation block at the band neutrophil stage was observed in the chronic idiopathic neutropenia patient (P-01) and 4 patients (P-02, P-04, P-07 and P-09) who were re-diagnosed with acquired neutropenia. The 4 patients with acquired neutropenia showed normal maturation with no myelokathexis (Supplementary Fig. S9).

**Figure 3:**
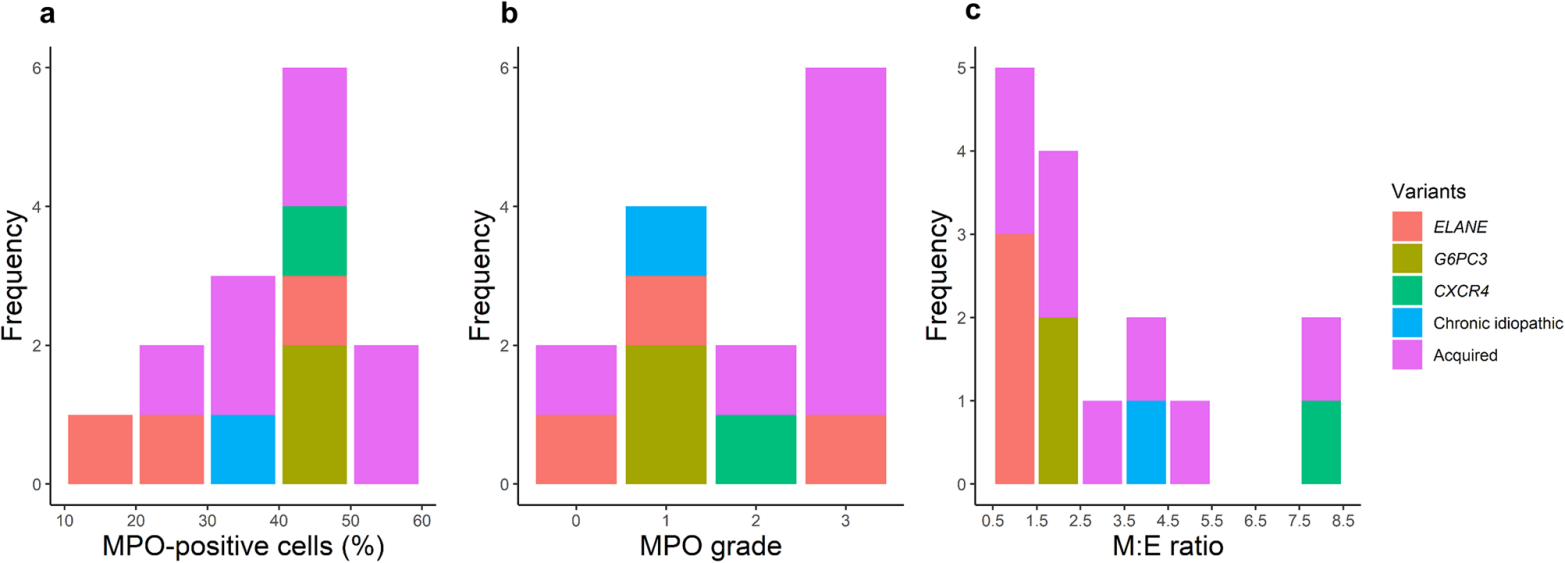
Distributions of MPO-positive cells, MPO grade and M:E ratio in the context of genetic variants. The percentage of MPO-positive cells ranged from 40 to 60% in patients with acquired neutropenia. Two of the 3 patients with pathogenic *ELANE* mutations showed 10%–30% of MPO-positive cells, while one patient showed 40%–50%. Three patients with pathogenic *G6PC3* and *CXCR4* mutations whose BM displayed myelokathexis had 40%–50% of MPO-positive cells. Two patients with VUS in *CXCR2* and acquired neutropenia showed 50%–60% of MPO-positive cells, which was higher than in patients with myelokathexis. The MPO grade of patients with acquired neutropenia was 2 or 3. Three patients with pathogenic *ELANE* mutations showed various distributions of MPO grade. BM with the pathogenic *CXCR4* mutation had MPO grade 2, whereas BM with *G6PC3* mutations had MPO grade 1. The M:E ratio of patients with *ELANE* mutations ranged from 0.5 to 1.5, which was lower than normal. *G6PC3*-mutated patients had an M:E ratio of 1.5–2.5, whereas a patient with the *CXCR4* mutation had an increased M:E ratio of 7.5–8.5.

We analyzed the distribution of MPO-positive cells, MPO grade and M:E ratio in the context of genetic variants. MPO-positive cells ranged from 20 to 60% in the patients with acquired neutropenia. Among patients with pathogenic *ELANE* mutations 3 showed 10%–30% MPO-positive cells and 1 patient showed 40%–50%. Three patients whose BM displayed myelokathexis and who had pathogenic *G6PC3* and *CXCR4* mutations showed 40%–50% MPO-positive cells. Three patients with pathogenic *ELANE* mutations showed various distribution of MPO grades. BM of the patient with a pathogenic *CXCR4* mutation showed MPO grade 2, whereas BM of patients with *G6PC3* mutations showed MPO grade 1. The M:E ratio of patients with *ELANE* mutations ranged from 0.5 to 1.5, which was lower than normal. That of the patients with *G6PC3* mutations was 1.5–2.5, and that of the patient with a *CXCR4* mutation was increased to 7.5–8.5 (Fig. 3).

### Congenital neutropenia: genetic and clinical aspects

Two comparisons were made in terms of genetic and clinical aspects. In comparison 1, the 16 patients were classified into 2 groups. Group 1–1 consisted of 7 patients with tier 1 variants and group 1–2 included the others. Permanent neutropenia (n = 5 in group 1–1 and n = 0 in group 1–2) and male patients (n = 7 in group 1–1 n = 1 in group 1–2) were more frequent in group 1–1 (*P* < 0.01 each). Severe infections were present at a higher percentage in group 1–1 (n = 6) than in group 1–2 (n = 1) (*P* < 0.01). ANC level at the latest follow-up was significantly lower in group 1–1 (median 0 × 10^9^/l) than in group 1–2 (median 1.797 × 10^9^/l) (*P* < 0.01). Early-stage maturation arrest was predominant in group 1–1 (n = 3 in group 1–1 and n = 0 in group 1–2), whereas late stage maturation arrest was predominant in group 1–2 (n = 0 in group 1–1 and n = 5 in group 1–2) (*P* < 0.05). Other clinical and histologic features such as the age when low ANC was discovered, family history of cancer or recurrent infections, fever pattern, G-CSF responsiveness, organomegaly, extra-hematopoietic features, initial ANC level, M:E ratio, BM cellularity, MPO-positive cells, MPO grade and myelokathexis were not significantly different. In comparison 2, 8 patients who did not achieve spontaneous recovery of ANC were categorized as group 2–1 and the other 8 patients who achieved spontaneous recovery of ANC were categorized as group 2–2. Like comparison 1, permanent neutropenia (n = 5 in group 2–1 and n = 0 in group 2–2), male patients (n = 7 in group 2–1 and n = 1 in group 2–2) and severe infections infections (n = 6 in group 2–1 and n = 1 in group 2–2) were more frequently observed in group 2–1 (*P* < 0.05, *P* < 0.01 and *P* < 0.01, respectively). The last ANC level was significantly higher in group 2–2 (median 2.246 × 10^9^/l) than in group 2–1 (median 0.026 × 10^9^/l) (*P* < 0.01). The number of G-CSF non-responders was significantly higher in group 2–1 (n = 6) than in group 2–2 (n = 1) (*P* < 0.05).

Although this difference was not statistically significant, 6 of 7 patients (85.7%) in group 2–1 were G-CSF non-responders, whereas 4 out of 5 (80.0%) in group 2–2 were G-CSF responders (*P* = 0.072). No patients in group 1–2 showed myelokathexis, whereas 3 of 7 (42.9%) patients in group 1–1 did (*P* = 0.063). Most patients in group 1–1 (n = 5, 83.3%) and group 2–1 (n = 6, 85.7%) showed MPO grade of 2 or less (*P* = 0.138), whereas most patients in group 1–2 (n = 5, 62.5%) and group 2–2 (n = 5, 71.4%) showed MPO grade 3 (*P* = 0.103) (Table 2).

**Table 2:** Comparisons between groups based on genetic evidence or spontaneous recovery of ANC.

	Comparison 1			Comparison 2		
	Group 1–1	Group 1–2	Statistical significance	Group 2–1	Group 2–2	Statistical significance
	Congenital neutropenia with causal variants (n = 7)	Neutropenia without pathogenic genetic evidence (n = 9)		No spontaneous recovery of ANC (n = 8)	Spontaneous recovery of ANC (n = 8)	
Age at initial presentation^*^ (month)	3.0 (0.5–28.0)	10.0 (6.0–17.0)	*P* = 0.222	3.5 (0.6–25.0)	10.0 (5.0–17.0)	*P* = 0.399
**Sex, n (%)**			*P* < .01			*P* < .01
Male	7 (100.0)	1 (11.1)		7 (87.5)	1 (12.5)	
Female	0 (0.0)	8 (88.9)		1 (12.5)	7 (87.5)	
**Family history, n(%)**			*P* = 1.000			*P* = 1.000
Yes	3 (42.9)	4 (44.4)		4 (50.0)	3 (37.5)	
No	4 (57.1)	5 (55.5)		4 (50.0)	5 (62.5)	
**Organomegaly, n (%)**			*P* = 1.000			*P* = 1.000
Yes	3 (42.9)	3 (33.3)		3 (37.5)	3 (37.5)	
No	4 (57.1)	6 (66.7)		5 (62.5)	5 (62.5)	
**Extra-hematopoietic feature, n (%)**			*P* = 0.106			*P* = 0.282
Yes	4 (57.1)	1 (11.1)		4 (50.0)	1 (12.5)	
No	3 (42.9)	8 (88.9)		4 (50.0)	7 (87.5)	
**Severe infection, n(%)**			*P* < .01			*P* < .01
Yes	6 (85.7)	1 (11.1)		6 (75.0)	1 (12.5)	
No	1 (14.3)	8 (88.9)		2 (25.0)	7 (87.5)	
**Fever pattern, n (%)**^†^			*P* = 0.592			*P* = 1.000
Neutropenic fever only	5 (71.4)	3 (33.3)		5 (62.5)	3 (37.5)	
Neutropenic fever and non-neutropenic fever	2 (28.6)	4 (44.4)		3 (37.5)	3 (37.5)	
**Neutropenia pattern, n (%)**			*P* < .01			*P* < .05
Permanent	5 (71.4)	0 (0.0)		5 (62.5)	0 (0.0)	
Intermittent	2 (28.6)	9 (100.0)		3 (37.5)	8 (100.0)	
**G-CSF responder, n (%)**^‡^			*P* = 0.242			*P* = 0.072
Yes	1 (14.3)	4 (44.4)		1 (12.5)	4 (50.0)	
No	5 (71.4)	2 (22.2)		6 (75.0)	1 (12.5)	
Initial ANC^*^	102 (0–958)	146 (32–540)	*P* = 0.872	124 (0–917)	157 (16–682)	*P* = 0.915
Last ANC^*^	0 (0–69)	1797 (1366–3892)	*P* < .01	26 (0–567)	2246 (1652–3946)	*P* < .01
M:E ratio^*§^	1.6 (0.6–3.4)	2.7 (1.3–4.1)	*P* = 0.194	1.9 (0.8–3.2)	2.4 (1.2–4.3)	*P* = 0.354
MPO-positive cell (%)^*^^¶^	42.38 (22.06–45.78)	42.65 (32.21–50.64)	*P* = 0.439	42.38 (26.45–46.05)	42.7 (28.5–50.6)	*P* = 0.259
MPO grade, n (%)^*^^¶^			*P* = 0.138			*P* = 0.103
Grade 0, 1 or 2	5 (71.4)	3 (33.3)		6 (0.75)	2 (25.0)	
Grade 3	1 (14.3)	5 (55.5)		1 (12.5)	5 (62.5)	
**Myelokathexis, n (%)**			*P* = 0.063			*P* = 0.200
Yes	3 (42.9)	0 (0.0)		3 (37.5)	0 (0.0)	
No	4 (57.1)	9 (100.0)		5 (62.5)	8 (100.0)	
**Maturation arrest, n (%)**			*P* < .05			*P* = 0.143
Early stage	3 (42.9)	0 (0.0)		3 (37.5)	1 (12.5)	
Late stage	0 (0.0)	5 (55.5)		0 (0.0)	4 (50.0)	

^*^Values presented as medians (interquartile ranges).

^†^Two patients who did not suffer from infection or fever during the follow-up period were excluded.

^‡^Three (18.8%) patients in whom G-CSF was not administered were not included. Also, one patient who could not assess the response to G-CSF were excluded.

^§^One patient with diluted BM aspiration in which reliable differential count could not be made was excluded.

^¶^Two patients were not included due to inadequate BM section quality or retrospectively unavailable BM paraffin block for MPO stain.

Abbreviations: BM, bone marrow; G-CSF, granulocyte-colony stimulating factor; M:E, myeloid to erythroid; MPO, myeloperoxidase.

### Comprehensive real-world data on neutropenia in Korean children

**Figure 4:**
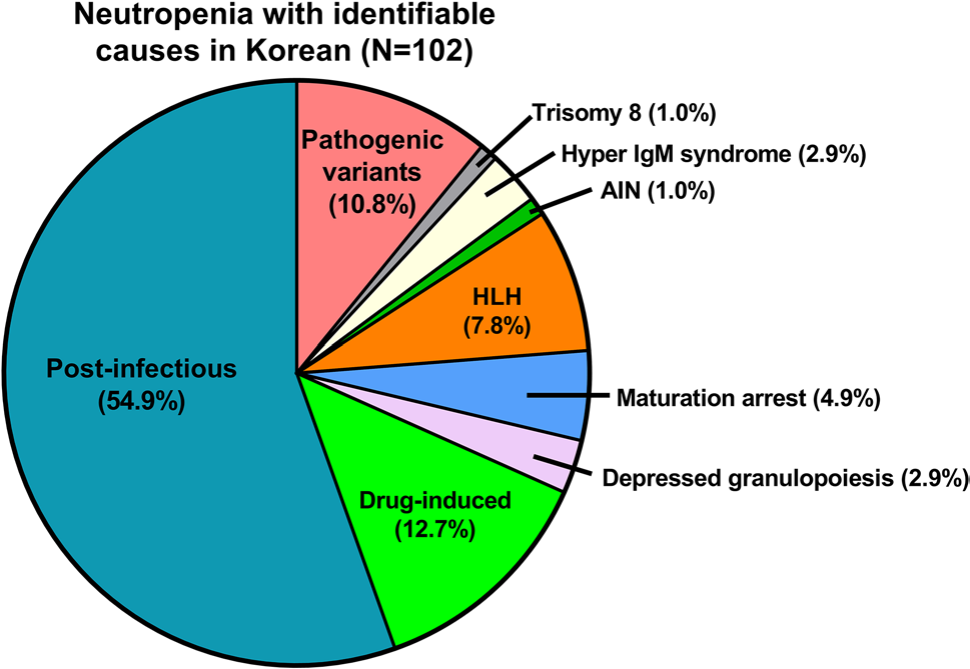
Real-world data on identifiable causes of neutropenia in Seoul National University Children’s Hospital from 2009 to 2018. Identifiable causes of neutropenia were detected in 102 out of 345 patients (29.6%). Post-infectious neutropenia was the most common (n = 56, 54.9%) followed by neutropenia with disease-causing variants (n = 11, 10.8%), drug-induced neutropenia (n = 13, 12.7%), hemophagocytic lymphohistiocytosis (n = 8, 7.8%), neutropenia due to maturation arrest (n = 5, 4.9%), neutropenia due to depressed granulopoiesis (n = 3, 2.9%), neutropenia with trisomy 8 (n = 1, 1.0%), hyper IgM syndrome (n = 1, 1.0%) and autoimmune neutropenia (n = 1, 1.0%). AIN, autoimmune neutropenia; HLH, hemophagocytic lymphohistiocytosis.

Including the 16 patients in this study, electronic medical records on a total of 345 neutropenia patients for the same period were reviewed. Two brothers with the same G6PC3 variant were counted as one person. Identifiable causes of neutropenia were detected in 102 out of 345 patients (29.6%). Post-infectious neutropenia was the most common (n = 56, 54.9%) followed by neutropenia with disease-causing variants (n = 11, 10.8%), drug-induced neutropenia (n = 13, 12.7%), hemophagocytic lymphohistiocytosis (n = 8, 7.8%), neutropenia due to maturation arrest (n = 5, 4.9%), neutropenia due to depressed granulopoiesis (n = 3, 2.9%), neutropenia with trisomy 8 (n = 1, 1.0%), hyper IgM syndrome (n = 3, 2.9%) and autoimmune neutropenia (n = 1, 1.0%) (Fig. 4).

Five patients who underwent genetic tests without BM exam harbored *ELANE* mutation (NM_001972.4:c.669C > A, p.(C223X) and c.455 T > C, p.(L152P)) in 2 patients, hemizygous *TAZ* mutations (NM_001348362.1:c.227delC, p.(P76fs) and NM_000116:c.350A > C, p.(K117T) ) in 2 male patients and a homozygous *SLC37A4* mutation (NM_001467.6):c.1179G > A, p.(W393X)) in one patient.

**Figure 5:**
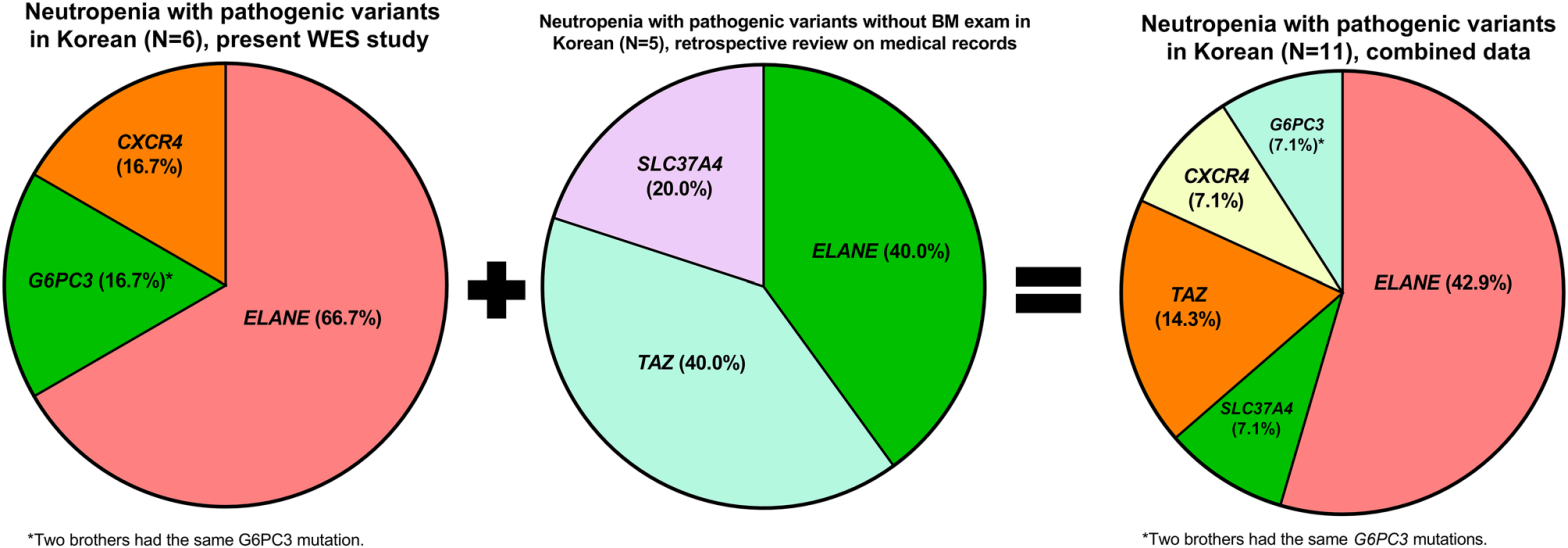
Comprehensive data on disease-causing genetic variants in 11 neutropenia patients obtained by combining this WES study results of the 6 patients and medical records of another 5 patients for whom BM exam was not performed. Focusing on the 11 patients with pathogenic genetic variants including 8 patients without BM exam, variants in an *ELANE* gene were the most common (n = 6, 42.9%), followed by *TAZ* (n = 2, 14.3%), *G6PC3* (n = 1, 7.1%), *CXCR4* (n = 1, 7.1%) and *SLC37A4* (n = 1, 7.1%).

Focusing on the 11 patients with pathogenic genetic variants including 8 patients without BM exam, variants in an *ELANE* gene were the most common (n = 6, 42.9%), followed by *TAZ* (n = 2, 14.3%), *G6PC3* (n = 1, 7.1%), *CXCR4* (n = 1, 7.1%) and *SLC37A4* (n = 1, 7.1%) (Fig. 5).

## Discussion

We performed WES or TS on 16 neutropenia patients and re-diagnosed each of them on the basis of genetic study, BM histology and clinical features. A heterogeneous genetic landscape was noted in the 7 neutropenia patients with pathogenic variants, two of whom were brothers who shared the same variant: *ELANE* (n = 4, 66.7%), *G6PC3* (n = 1, 16.7%) and *CXCR4* (n = 1, 16.7%).

The 4 patients with *ELANE* mutations showed permanent neutropenia with early-stage maturation arrest and no extra-hematopoietic manifestations. An *ELANE* mutation is associated with the most serious infectious complications^24^, and one of the *ELANE*-mutated patients, who was the only non-survivor among the 16 neutropenia patients, died of sepsis. Two patients with the same *ELANE* mutation (G214R) showed totally different BM histology and clinical course, which suggests that the same mutation does not necessarily lead to the same or similar clinical phenotype or BM histology. *ELANE* mutations with the same codon change are known to result in different clinical phenotypes. For example, ELANE p.(F43L), p.(A61V), p.(V101M), p.(S126L), p.(S126W), p.(P139L), p.(Q194X), IVS4 + 1G > A, IVS4 + 5G > A, p.(G214X), p.(R220Q) and p.(Y228X) can manifest as CN that can develop into MDS or AML, while they can also result in cyclic neutropenia with a relatively good prognosis^25^. In a case report on Korean patients with the same *ELANE* mutation (NM_001982.2:c.591 + 1G > A), different clinical phenotypes were found even within the same family^22^. In the present study, low MPO-positive cells and MPO grade might have affected poor prognosis due to decreased granulopoietic cell reservoir.

In the present study, a novel variant in *G6PC3* (NM_138387.3):c.214delA, p.(K72fs)) was discovered in two brothers. Of note, one of them showed an extra-hematopoietic feature of juvenile rheumatoid arthritis, which has not been reported as a consequence of *G6PC3* mutations. Furthermore, they showed myelokathexis, which has been reported as rare in patients with a *G6PC3* mutation^26^. Both brothers showed very similar clinical features and BM histology. The CNV analysis of these brothers detected the CN-LOH of the 17q21.31 region (chr17:45,287,005–48,276,944 in P-13 and chr17:44,771,405–48,276,944 in P-14), disclosing the mechanism of homozygous *G6PC3* mutations. Although we could not perform a trio study due to the unwillingness of their parents, we inferred the possible causes and mechanisms as follows. Among 22 autosomal chromosomes, homozygosity was observed only in chromosome 17 (data not shown), which tentatively rules out consanguinity. Chromosome 17 with CN-LOH at 17q21.31 might have been monosomy- or trisomy-rescued or a gamete carrying CN-LOH at 17q21.31 with non-disjunction might have been inherited^27^. On the basis of the same CN-LOH pattern of chromosome 17 in both brothers we may attribute the autosomal recessive CN to either maternal or paternal whole uniparental isodisomy of chromosome 17 rather than to two carrier parents with the same mutation. (Supplementary Fig. S10).

Regarding the BM histology, myelokathexis was a common feature in patients with *CXCR4* and *G6PC3* mutations. However, the former showed permanent neutropenia and dysplastic neutrophils, whereas the latter had intermittent neutropenia with no distinct dysplasia. The two brothers with *G6PC3* mutations showed very similar BM histology of myelokathexis with a strikingly increased proportion of old neutrophils in comparison with patients carrying *CXCR4* mutations, who showed relatively overall increase in the number of myeloid cells at each stage. The difference in myelokathexis features between patients with *CXCR4* and *G6PC3* mutations might have resulted from different mutated genes. Alternatively, it might be attributable to the different age when the BM exam was performed: the patient with the *CXCR4* mutation was 21 months old, whereas the *G6PC3* patients were 19 and 21 years old and therefore might have had a more advanced state of myelokathexis.

One of the 16 patients (P-01) was assessed as chronic idiopathic neutropenia because we could not find any related pathogenic candidate variants. She had intermittent neutropenia for more than 3 years; her BM exam revealed a low percentage of MPO-positive cells with MPO grade 1. We infer that considering the clinical course, there might be a veiled cause for her neutropenia, although it was not identified in this study by WES.

A total of 8 patients were re-diagnosed as acquired neutropenia. Pathogenic disease-causing variants were not detected in those patients who showed clinically benign course, which supported the likelihood of acquired or transient neutropenia. However, it would be possible that they have CN due to inherited variants in novel genes not yet associated with CN. So, they can be reassessed in the future when more genomic databases are available.

Meanwhile, for BM histologic assessment, we analyzed absolute count of MPO-positive cells in BM section using ImageJ and MPO grade. MPO-positive cell count in BM sections is not influenced by peripheral blood dilution or site variation in BM. For that reason, the amount of granulopoiesis is assessed more accurately in BM sections rather than in BM aspirates. Also, it might be useful to assess the absolute numbers of myeloid cells compared to the M:E ratio which is relative and is largely influenced by erythroid values and hemodilution. The MPO grade assessment on BM sections aids in evaluating the objective myelopoiesis status. Moreover, topological observation of myeloid cells near the trabecular bone helps us to determine whether neutropenia arises from defective production or BM retention.

We examined a relationship between genotype and BM histology based on the percentage of MPO-positive cells, MPO grade and M:E ratio. Patients with *ELANE* mutations showed variable ranges of MPO-positive cells and MPO grades, while their M:E ratios were consistently low (0.5–1.5), indicating that although the absolute numbers of MPO-positive cells are variable, myeloid cells tend to be relatively fewer than erythroid cells. We speculate that this might be attributable to apoptosis of myeloid precursors in patients with *ELANE* mutations. On the other hand, in the cases of myelokathexis caused by *CXCR4* and *G6PC3* mutations, MPO-positive cells constituted 40%–50% with MPO grade 1 or 2. The M:E ratio was increased in the patient with a *CXCR4* mutation (M:E ratio, 7.9), but it was not in patients with *G6PC3* mutations (M:E ratio, 1.5–2.5). These observations imply that the M:E ratio is not reliable for the assessment of myelokathexis. Instead, the combination of the percentage of MPO-positive cells and low MPO grade might be helpful for the evaluation of the BM retention status. Although information on MPO-positive cells, MPO grade and M:E ratio provides clues to the BM granulopoiesis status, confirmation by genetic study is necessary for the accurate diagnosis of CN.

Clinically significant variants might have been missed in our study because of methodological limitations. Our CNV analysis detected no clinically significant gains or losses, contrary to the reported detection of 16.4% of pathogenic CNVs in IBMF patients^28^. WES-based CNV analysis by ExomeDepth has been reported to have sensitivity of approximately 40% for deletions and 30% for duplications compared to chromosomal microarray, which is the current gold-standard method for CNV analysis^29^. Pathogenic variants located in deep introns or variants with mosaicism might have been missed either.

Real-world data on neutropenia patients and their diagnostic work-up showed the pitfalls of the diagnostic algorithm for CN in our hospital, in which immunological work-up is not performed routinely. Meanwhile, a retrospective review on medical records revealed pathogenic variants in *ELANE*, *TAZ* and *SLC37A4* in 5 patients. Patients with *TAZ* or *SLC37A4* mutations showed cardiomyopathy or glycogen storage disease as their main clinical phenotype, respectively. Considering the heterogeneity of CN in terms of genotypes and phenotypes, WES or expanded next generation sequencing panel which covers genes related to immunodeficiency and IBMF as well as CN would be necessary.

## Methods

### Patients

A total of 16 patients who visited Seoul National University Children’s Hospital from 2009 to 2018 and were diagnosed with CN based on BM histology and clinical information were enrolled. Fifteen BM aspirates and one peripheral blood sample were retrospectively collected from the 16 pediatric patients. Data on patients’ clinical course, family history and laboratory results such as CBC and Ig levels were retrospectively reviewed using electronic medical records.

Meanwhile, comprehensive review on real-world data of 345 pediatric patients with neutropenia including 16 patients in this study during the same period was performed. Ig levels were measured in 76 out of 345 (22.0%) patients. The patients were classified into 4 groups: patients who underwent both BM and genetic study, only BM exam, only genetic test and those who underwent none of the tests. Sanger sequencing was performed on *ELANE* in 38 patients, *TAZ* in 1 patient and *SLC37A4* in 1 patient. Meanwhile, targeted next generation sequencing of which panels included *ELANE* or *TAZ* was performed on 2 patients (Supplementary Figure S11).

This study was approved by the institutional review board (IRB) of Seoul National University Hospital (IRB No. 1912-099-1089). The research was performed in accordance with the Declaration of Helsinki. The requirement for obtaining informed consent was waived due to the retrospective nature of this study by the IRB of Seoul National University Hospital.

### Immunohistochemical staining for myeloperoxidase

MPO was stained in BM biopsy specimens. A paraffin-embedded tissue block was trimmed and sliced into 2-μm sections. The tissues on slides were incubated at 56 °C for 30 min and hydrated with xylene, 100% ethanol (EtOH), 95% EtOH and 70% EtOH. Each slide was then stained with Ventana BenchMark ULTRA (Ventana Medical Systems Inc., Tucson, AZ, USA). Polyclonal rabbit anti-human MPO antibody (DAKO, Glostrup, Denmark) was applied for 15 min at room temperature. Subsequently, the slides were dehydrated using 70% EtOH, 95% EtOH, 100% EtOH and xylene.

### MPO-positive cell count

Digital images of MPO-stained BM Sects. (200 ×) were captured with a Zeiss AxioCAM microscope (Zeiss, Oberkochen, Germany). MPO-positive cells were counted using ImageJ software (https://imagej.nih.gov/ij/). Two or three images per patient were analyzed to minimize the bias from site variation or suboptimal BM section quality. On average, 7,151 nucleated cells (range, 4,369–11,513) per patient were counted. MPO-positive cells were detected by analyzing particle counts. The minimum size of an MPO-positive cell was set from 8 to 40 pixel^2^ depending on the individual cell size variations; the minimum size of a BM nucleated cell was set to 10–20 pixel^2^. The maximum size of cells was set to 20,000 pixel^2^ to exclude non-cell components such as fat or trabecular bone. Hue, saturation and brightness were adjusted to accurately call MPO-positive cells as a numerator and nucleated cells as a denominator. The percentage of MPO-positive cells was calculated by dividing the number of MPO-positive cells by that of nucleated cells and multiplying the result by 100. Non-specific signals were excluded (Supplementary Fig. S12 and Table S3).

### Bone marrow histology

BM aspirates and biopsies of the 16 neutropenia patients were retrospectively reviewed. Maturation arrest of the granulocytic lineage and myelokathexis were assessed according to the pediatric age-specific reference range of BM differential count^30,31^. Myelokathexis was defined as the BM retention status with an increased sum of band plus segmented neutrophils in comparison with the reference range (Supplementary Fig. S13).

MPO grade was arbitrarily defined as the number of layers of MPO-positive cells along the trabecular bones: grade 0, < 1 layer; grade 1, < 2 layers; grade 2, < 3 layers; and grade 3, ≥ 3 layers (Supplementary Fig. S14).

### G-banding analysis

Chromosome analysis was performed as previously described^32^.

### Whole-exome sequencing

The SureSelectHuman All Exon V5 + UTR probe set (Agilent, Santa Clara, CA, USA) included 359,555 exons of 21,522 genes; the size of the total targeted region was 75 Mb. To generate standard exome capture libraries, the Agilent SureSelect Target Enrichment protocol for Illumina paired-end sequencing library (ver. B.3, June 2015) was used with 3 µg of input genomic DNA. The DNA was quantified and its quality was assessed by PicoGreen (Thermo Fisher Scientific, Waltham, MA, USA) and Nanodrop (Thermo Fisher Scientific). Fragmentation of 1 µg of genomic DNA was performed using adaptive focused acoustic technology (AFA; Covaris). The fragmented DNA is repaired, an ‘A’ is ligated to the 3′ end, and Agilent adapters are then ligated to the fragments. Once ligation had been assessed, the adapter-ligated product is PCR amplified. The final purified product is then quantified using qPCR according to the qPCR Quantification Protocol Guide and its quality was assessed using the Caliper LabChipHigh Sensitivity DNA kit (PerkinElmer, Waltham, MA, USA). For exome capture, 250 ng of DNA library was mixed with hybridization buffers, blocking mixes, RNase block and 5 µl of the SureSelect all exon capture library according to the standard Agilent SureSelect Target Enrichment protocol. Hybridization to the capture baits was conducted at 65 °C using the heated thermal cycler lid option at 105 °C for 24 h in a PCR machine. The captured DNA was then amplified. The final purified product was quantified using qPCR according to the qPCR Quantification Protocol Guide and its quality was assessed using the TapeStation DNA ScreenTape (Agilent, Santa Clara, CA, USA). Pooled DNA libraries were then sequenced using the HiSeq 2500 platform (Illumina, San Diego, CA, USA).

### Targeted sequencing

Targeted sequencing using an in-house panel of 507 genes related to hematologic malignancies and other cancers was performed in one patient (P-08) as previously described^32^. The panel included 182 out of 500 genes related to CN (20/29, 69.0%), IBMF (41/55, 74.5%), immunodeficiency (80/399, 20.1%) and cancer predisposition (41/77, 53.2%) (Supplementary Table S4).

### Variant analysis strategies

Low-quality data with a total depth of < 10, the number of reads with alternative allele of < 2 or variant allele frequency < 0.1 were removed. Variants with minor allele frequency of > 0.01 were filtered out based on the 1000 Genome, Exome Aggregation Consortium, Exome Sequencing Project v. 6500, Genome Aggregation Database, Korean Reference Genome Database and Korean Variant Archive. Synonymous and non-coding region variants were also eliminated while invariant splice site variants which are located on ± 2 base position from exons were kept. Variant allele frequencies of 30%–70% and 90%–100% were considered as indicative of candidate heterozygous and homozygous variants, respectively. A total of 500 genes were used for filtering in candidate variants: 29 genes known to cause CN, 55 genes related to IBMF, 339 genes associated with immunodeficiency and 77 cancer predisposition genes (Supplementary Table S4). Pathogenicity was assessed according to the American College of Medical Genetics and Genomics and the Association for Molecular Pathology guidelines^33^ (Supplementary Fig. S15).

### Copy-number variant analysis

CNV analysis of the 15 patients who underwent WES was conducted using Nexus Copy Number (version 10.0, BioDiscovery). A reference file was made by MUltiScale BAM Reference Builder. Using BAM files of the 4 male patients with *ELANE* pathogenic mutations as controls, CNV analysis of the other 11 patients was carried out. A total of 3,997 autosomal and sex-chromosomal CNVs, which included copy number gain, copy number loss, high copy number gain and homozygous copy loss, were called automatically by using pre-set thresholds provided by the software. We used strict probe median values for filtering in the candidate CNVs (Supplementary Fig. S16). Consequently, 100 copy number gains, 63 copy number losses, 102 high copy number gains and 13 homozygous losses in autosomal and sex chromosomes were detected. The pathogenicity of each CNV was assessed according to the American College of Medical Genetics and Genomics and the Clinical Genome Resource guidelines^34^. Conversely, BAM files of 4 female patients (P-02, P-04, P-06 and P-07) were used as controls to analyze the CNVs of the 4 patients with *ELANE* pathogenic mutations. Using the same methods as above, 46 copy number gains, 15 copy number losses, 29 high copy number gains and 9 homozygous losses in autosomal and sex chromosomes were detected (Supplementary Fig. S16). In the case of suspected LOH, manual inspection of the B-allele frequency plots of the chromosomal region was performed.

### Statistical analysis

Continuous data were presented as medians and interquartile ranges (IQRs). For comparisons of continuous variables, Mann–Whitney and Kruskal–Wallis tests were performed. Chi-square test was used for comparisons of non-continuous variables between groups. Correlation analysis was carried out using Spearman’s correlation. Statistical significance was defined as *P* < 0.05. The SPSS version 25.0 software program (SPSS Inc., Chicago, IL, USA), GraphPad Prism 8.0 (GraphPad Software, San Diego, CA, USA) and R package version 4.0.1 were used.

## Supplementary Information

Supplementary Information.

## Data Availability

The data and materials used in this study are available upon request to qualified researchers.
