# Author Correction: Heterogeneous genetic landscape of congenital neutropenia in Korean patients revealed by whole exome sequencing: genetic, phenotypic and histologic correlations

**DOI:** 10.1038/s41598-022-26128-8

**Published:** 2022-12-13

**Authors:** Dajeong Jeong, Sung-Min Kim, Byung Joo Min, Ju Han Kim, Young Seok Ju, Yong-Oon Ahn, Jiwon Yun, Young Eun Lee, Seok Ryun Kwon, Jae Hyeon Park, Jong Hyun Yoon, Dong Soon Lee

**Affiliations:** 1 Department of Laboratory Medicine, Seoul National University Hospital, Seoul National University College of Medicine, 101, Daehak-Ro Jongno-Gu, Seoul, 110-744 Republic of Korea; 2 Cancer Research Institute, Seoul National University College of Medicine, Seoul, Republic of Korea; 3 Division of Biomedical Informatics, Seoul National University College of Medicine, Seoul, Republic of Korea; 4 Graduate School of Medical Science and Engineering, Korea Advanced Institute of Science and Technology, Daejeon, 34141 Republic of Korea; 5 Department of Laboratory Medicine, Seoul National University Boramae Medical Center, Seoul, Republic of Korea

Correction to: *Scientific Reports* 10.1038/s41598-022-11492-2, published online 07 May 2022

The original version of this Article contained an error in the Methods section. The IRB number was incorrect.

“This study was approved by the institutional review board (IRB) of Seoul National University Hospital (IRB No. 2001-139-1096).”

now reads:

“This study was approved by the institutional review board (IRB) of Seoul National University Hospital (IRB No. 1912-099-1089).”

The original Article has been corrected.

