# Supplementary material for: Heterogeneous genetic landscape of congenital neutropenia in Korean patients revealed by whole exome sequencing: genetic, phenotypic and histologic correlations
Source: Sci Rep. 2022 May 7;12:7515. doi: 10.1038/s41598-022-11492-2 (PMC9079068; doi:10.1038/s41598-022-11492-2)
Supplement: Supplementary file 1 — Supplementary Information. [file 41598_2022_11492_MOESM1_ESM.docx]

**Supplementary information**

**Heterogeneous genetic landscape of congenital neutropenia in Korean patients revealed by whole exome sequencing: Genetic, phenotypic and histologic correlations**

Dajeong Jeong^1,6^, Sung-Min Kim^2,6^, ByungJoo Min^3^, Ju Han Kim^3^, Young Seok Ju^4^, Yong-Oon Ahn^2^, Jiwon Yun^1^, Young Eun Lee^1^, Seok ryun Kwon^1^, Jae Hyeon Park^1^, Jong Hyun Yoon^1,5^, Dong Soon Lee^1,2*^

^1^ Department of Laboratory Medicine, Seoul National University Hospital, Seoul, Korea

^2^ Cancer Research Institute, Seoul National University College of Medicine, Seoul, Republic of Korea

^3^ Division of Biomedical Informatics, Seoul National University College of Medicine, Seoul, Republic of Korea

^4^ Graduate School of Medical Science and Engineering, Korea Advanced Institute of Science and Technology, Daejeon 34141, Republic of Korea

^5^ Department of Laboratory Medicine, Seoul National University Boramae Medical Center, Seoul, Republic of Korea

^6^ These authors contributed equally

**Corresponding Author:** Dong Soon Lee, MD., PhD.

E-mail: soonlee@snu.ac.kr

**Contents**

**Abbreviation**

**Supplementary Figures**

**Figure S1.** Distributions of MPO-positive cells analyzed by ImageJ and M:E ratio according to MPO grade.

**Figure S2.** The log R ratio and B allele frequency plots of chromosome 17 in two brothers (P-13 and P-14) with the same *G6PC3* mutation (NM_138387.3:c.214delA, p.(K72fs)).

**Figure S3.** The *ELANE* (G214R) mutation in two patients showing different BM histologies.

**Figure S4** Different clinical course of two patients (P-05 and P-11) who harbored the same pathogenic *ELANE* variant (NM_001972.2:c.640G>A, p.(G214R)).

**Figure S5** Myelokathexis in patients with *CXCR4* and *G6PC3* mutations.

**Figure S6** ANC changes and clinical course of the patient (P-08) with a *CXCR4* mutation.

**Figure S7** ANC changes and clinical course of two brothers with the same *G6PC3* mutation.

**Figure S8** BM features and clinical course of the patient (P-01) who showed chronic idiopathic neutropenia

**Figure S9** Genotype–BM histology correlations in 16 neutropenia patients

**Figure S10** Four hypotheses for the occurrence of homozygous *G6PC3* mutations by CN-LOH.

**Figure S11** Real-world data on diagnostic work-up algorithms for neutropenia patients in Seoul National University Children’s Hospital from 2009 to 2018.

**Figure S12** MPO-positive cell count using ImageJ. Distributions of MPO-positive cells analyzed by ImageJ and M:E ratio according to MPO grade.

**Figure S13** Maturation arrest and myelokathexis assessment according to the pediatric age–specific reference range of BM differential count.

**Figure S14** BM section images showing different MPO grades

**Figure S15** Strategies for whole-exome or targeted sequencing variant analysis to search for disease-causing variants in 16 neutropenia patients

**Figure S16** CNV analysis strategies in 15 congenital neutropenia patients who underwent WES.

**Supplementary Tables**

**Table S1** Variants detected in the 16 neutropenia patients including VUS

**Table S2** CNV analysis of 15 patients for whom WES was performed

**Table S3** Enumeration of MPO-positive cells on bone marrow section using ImageJ

**Table S4** List of 500 genes selected for variant analysis

**Abbreviations**

AD: autosomal dominant; AML: acute myeloid leukemia; ANC: absolute neutropenia count; AR: autosomal recessive; BM: bone marrow; CBC: complete blood count; CN: congenital neutropenia; CN-LOH: copy-neutral loss of heterozygosity; CNV: copy-number variant; G-CSF: granulocyte-colony stimulating factor; hPBSCT: haploidentical peripheral blood stem cell transplantation; HSCT: hematopoietic stem cell transplantation; IBMF: inherited bone marrow failure; Ig: immunoglobulin; IQR: interquartile range; M:E: myeloid-to-erythroid ; MDS: myelodysplastic syndrome ; MPO: myeloperoxidase; PB: peripheral blood; PBSCT: peripheral blood stem cell transplantation; VUS: variant of unknown significance; WES: whole-exome sequencing; XR: X-linked recessive.

**
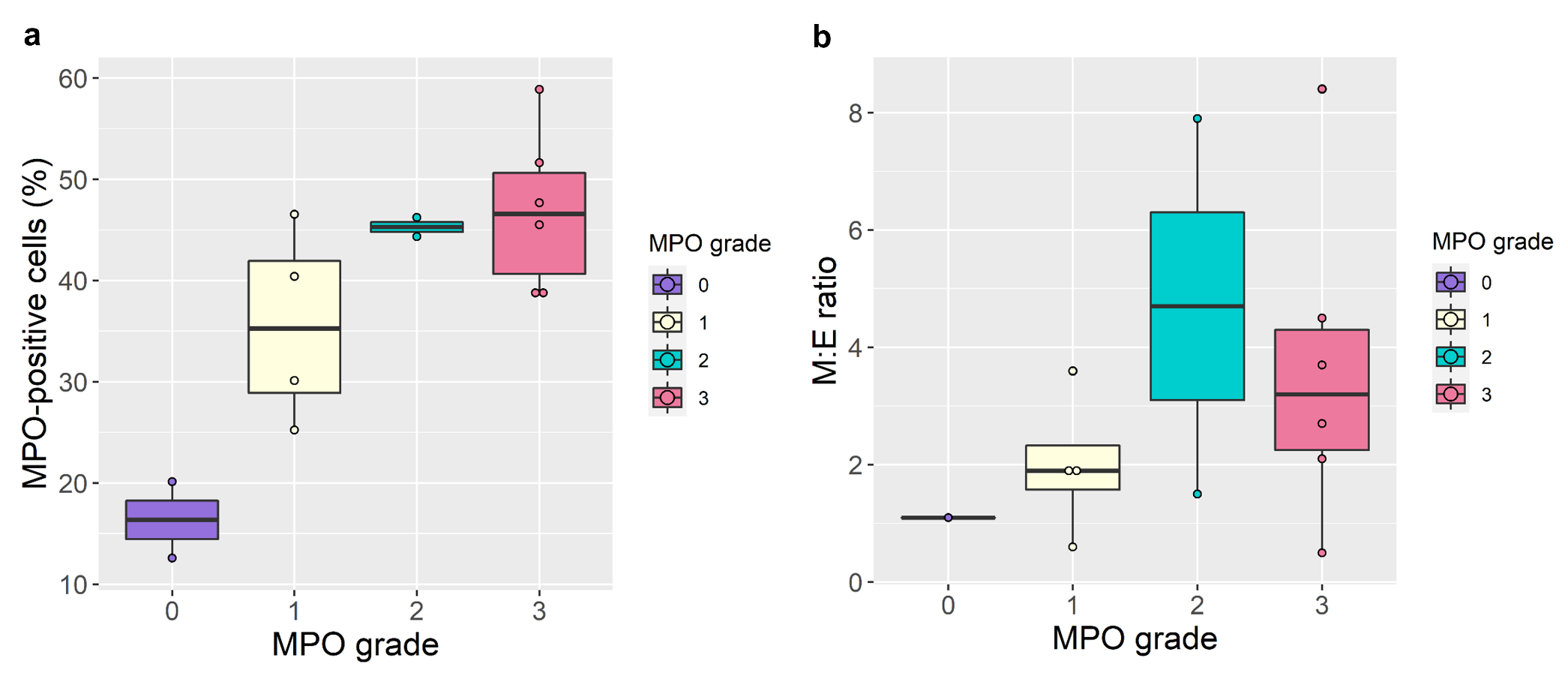
**

**Figure S1.** **Distributions of MPO-positive cells analyzed by ImageJ and M:E ratio according to MPO grade.** (a) A tendency toward positive correlation between MPO grade and the percentage of MPO-positive cells (*P* = 0.086). (b) The absence of distinct association between MPO grade and M:E ratio (*P* = 0.477). M:E, myeloid-to-erythroid; MPO, myeloperoxidase.


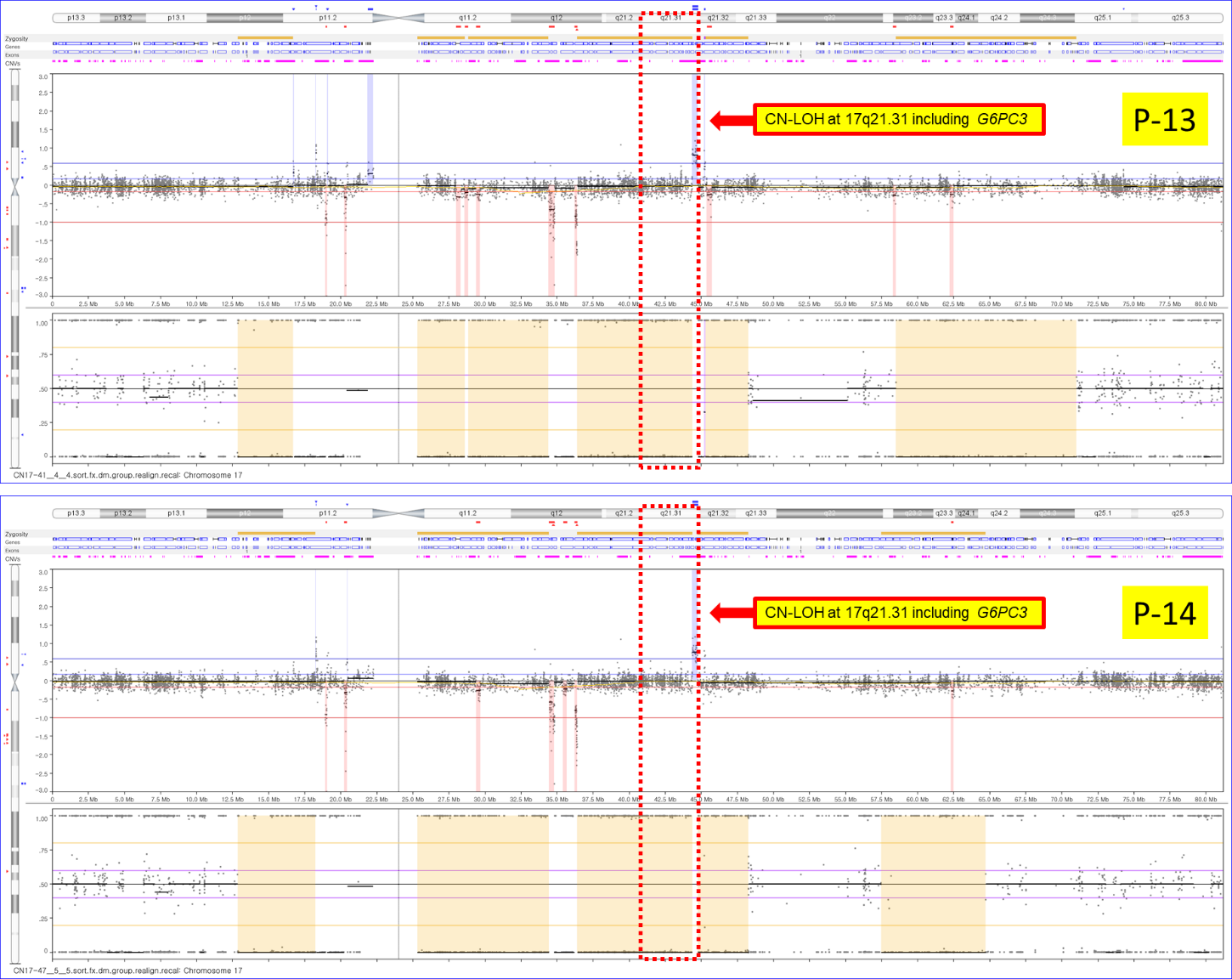


**Figure S2.** **The log R ratio and B allele frequency plots of chromosome 17 in two brothers (P-13 and P-14) with the same *G6PC3* mutation (NM_138387.3:c.214delA, p.(K72fs)).** CN-LOH, copy-neutral loss of heterozygosity.


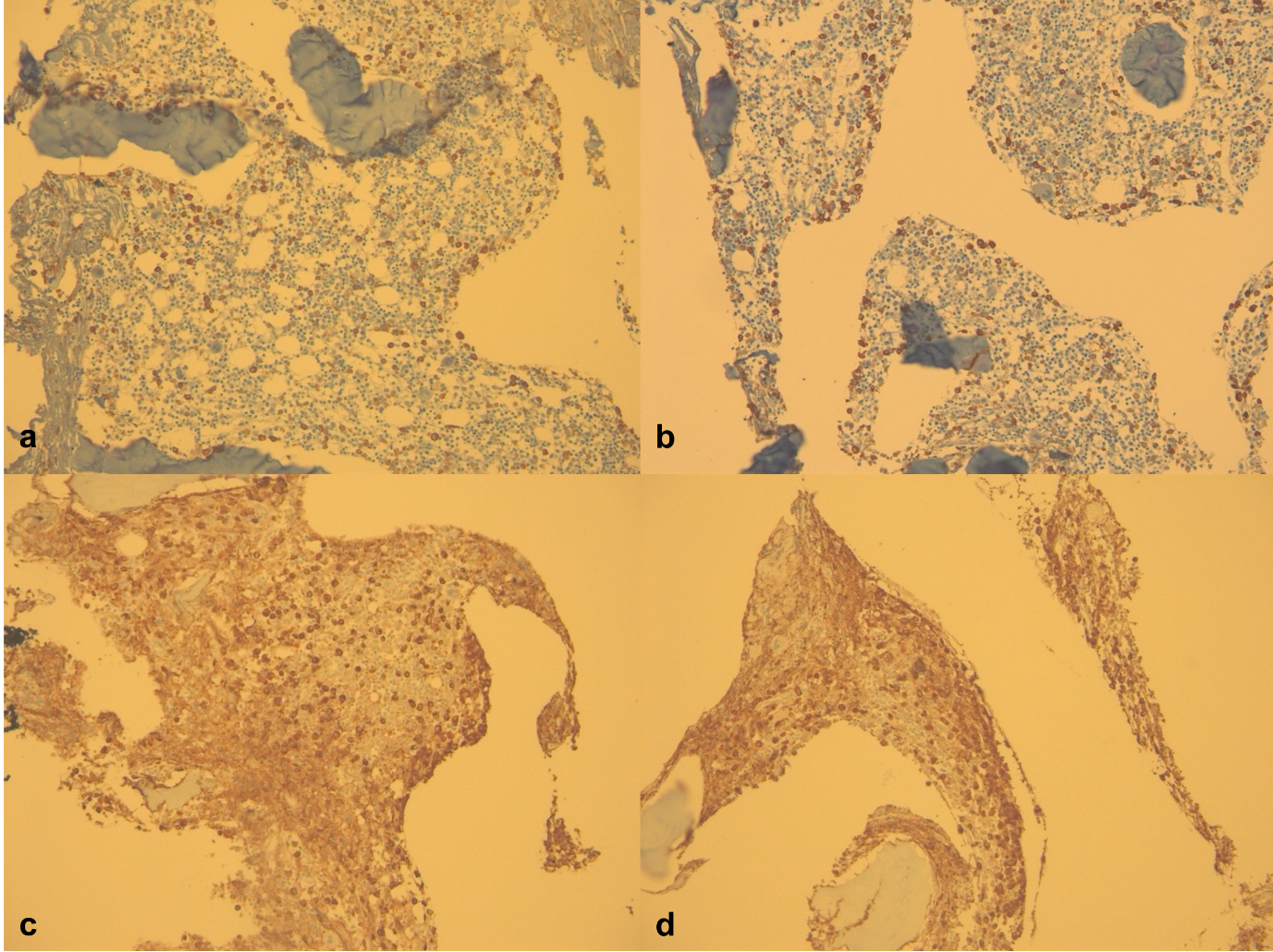


**Figure S3. The *ELANE* (****NM_001972.2:c.640G>A, p.(G214R)) mutation in two patients showing different BM histologies.** (a-b) Patient P-05 had 12.6% of MPO-positive cells and MPO grade 0. (c-d) Patient P-11 had 45.5% of MPO-positive cells and MPO grade 3. MPO stain, ×200. MPO, myeloperoxidase


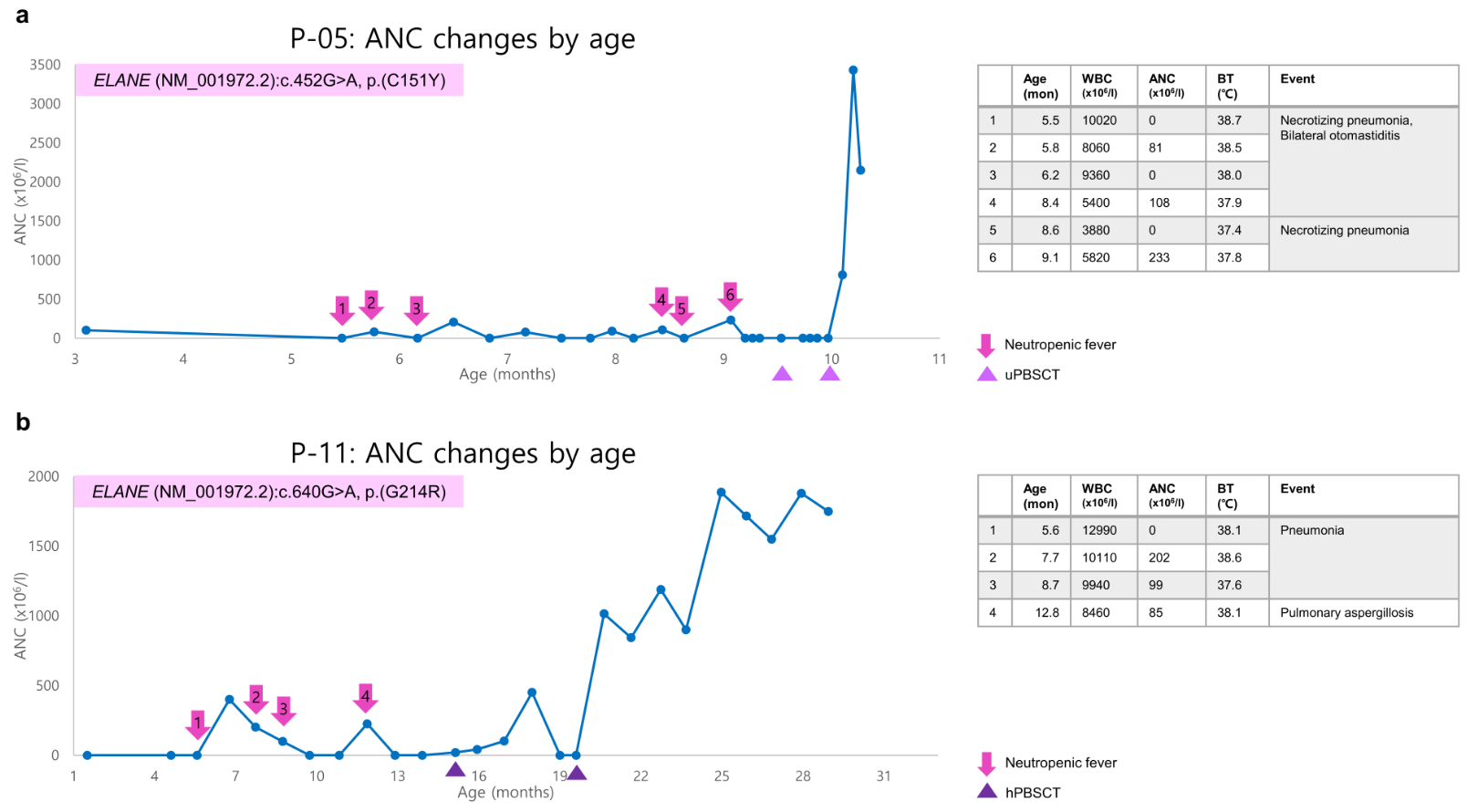


**Figure S4.** **Different clinical course of two patients (P-05 and P-11) who harbored the same pathogenic *ELANE* variant (NM_001972.2):c.640G>A, p.(G214R)).** (a) P-05 underwent two uPBSCT, but he died of sepsis. (b) P-11 had successful second hPBSCT, which led to ANC level recovery. ANC, absolute neutrophil count; BT, body temperature; hPBSCT, haploidentical peripheral blood stem cell transplantations; uPBSCT, unrelated peripheral blood stem cell transplantations; WBC, white blood cell.


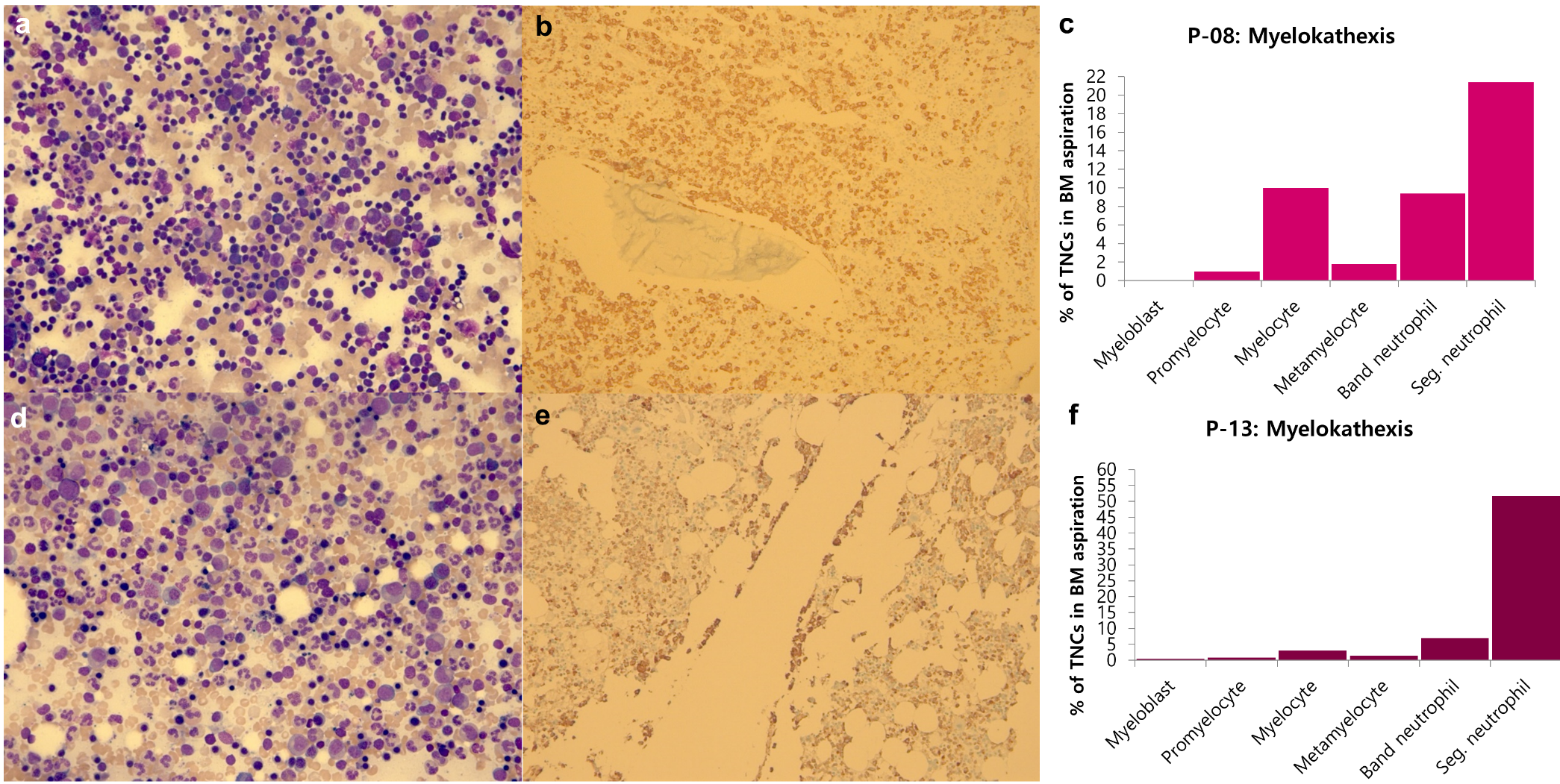


**Figure S5.** **Myelokathexis in patients with *CXCR4* and *G6PC3* mutations.** (a-c) Patient P-08 with *CXCR4* mutation. (d-f) Patient P-13 with *G6PC3* mutation. (a,d) Wright-Giemsa stain, ×200 (b,e) MPO stain, ×200. BM, bone marrow; MPO, myeloperoxidase; TNC, total nucleated cells.


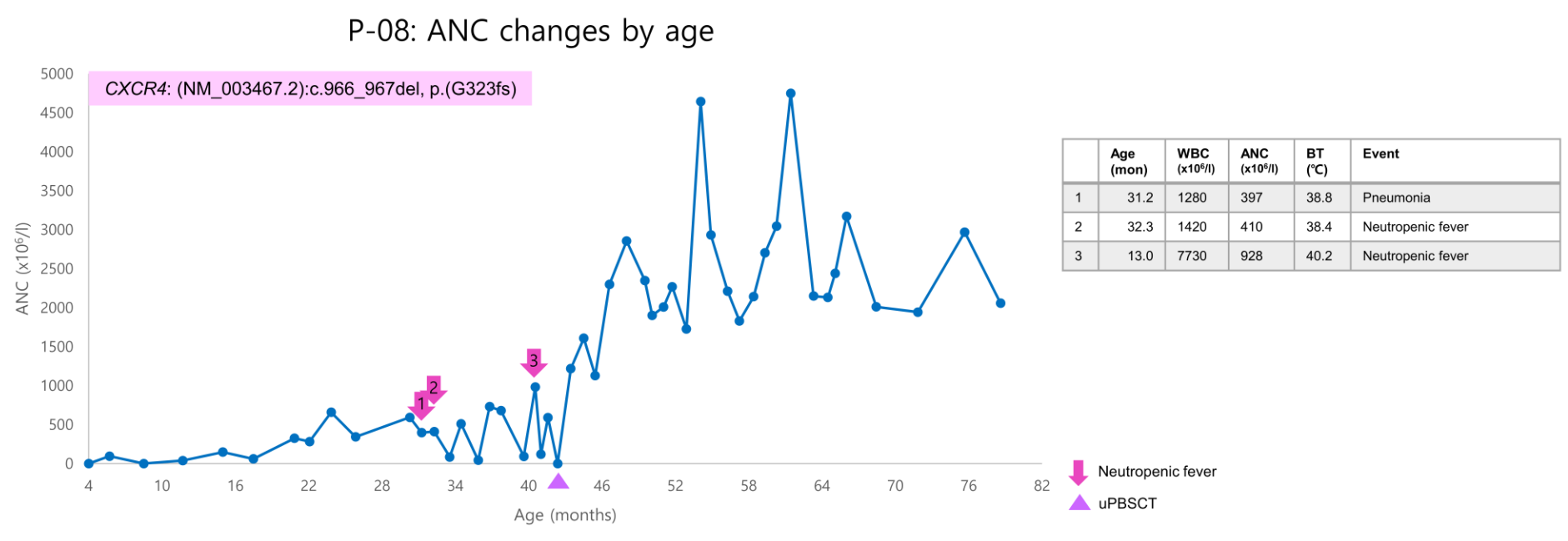


**Figure S6. ANC changes and clinical course of the patient (P-08) with a *CXCR4* mutation.** ANC, absolute neutrophil count; BT, body temperature; uPBSCT, unrelated peripheral blood stem cell transplantations; WBC, white blood cell.


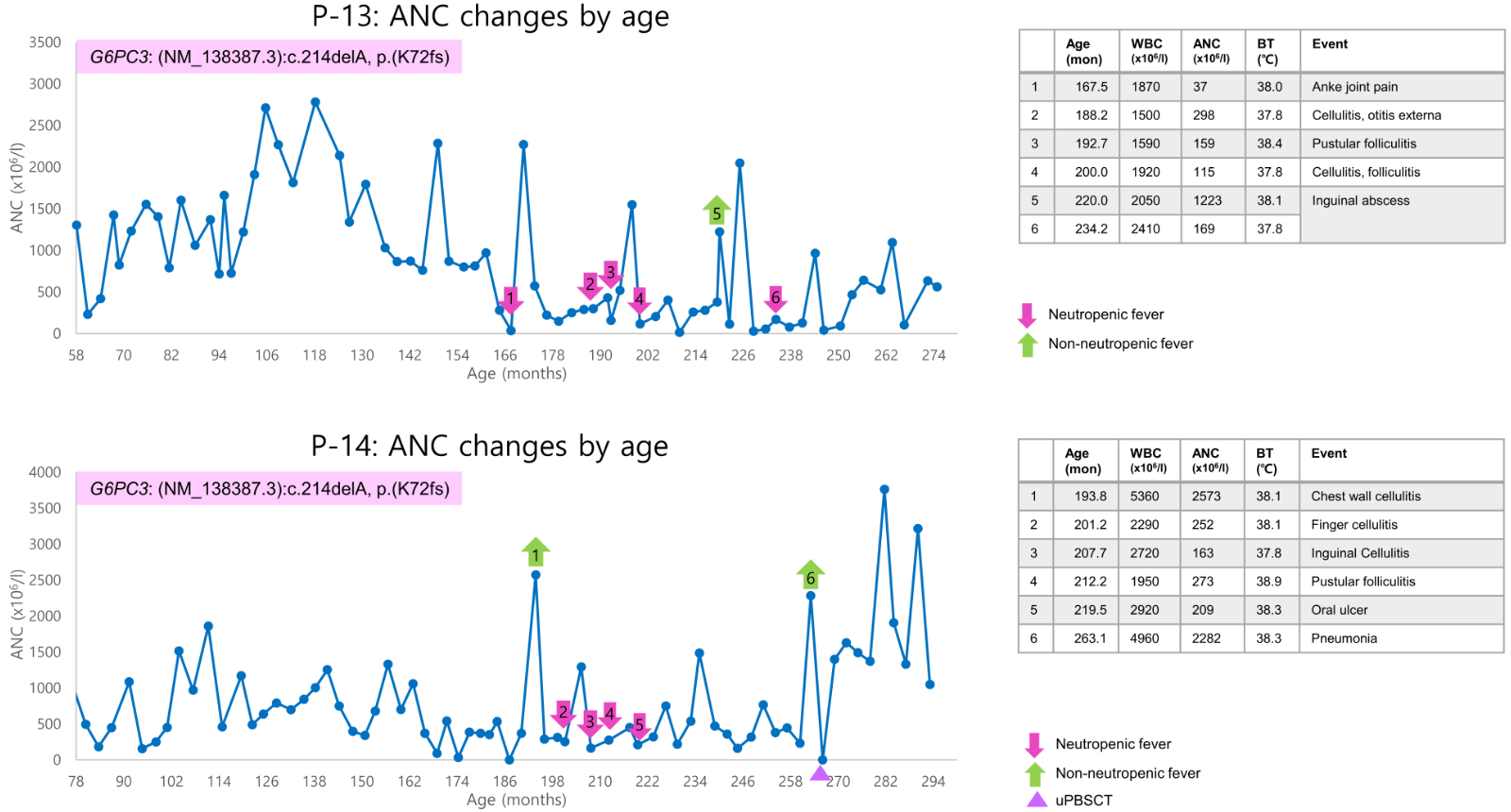


**Figure S7.** **ANC changes and clinical course of two brothers with the same *G6PC3* mutation.** ANC, absolute neutrophil count; BT, body temperature; uPBSCT, unrelated peripheral blood stem cell transplantations; WBC, white blood cell.


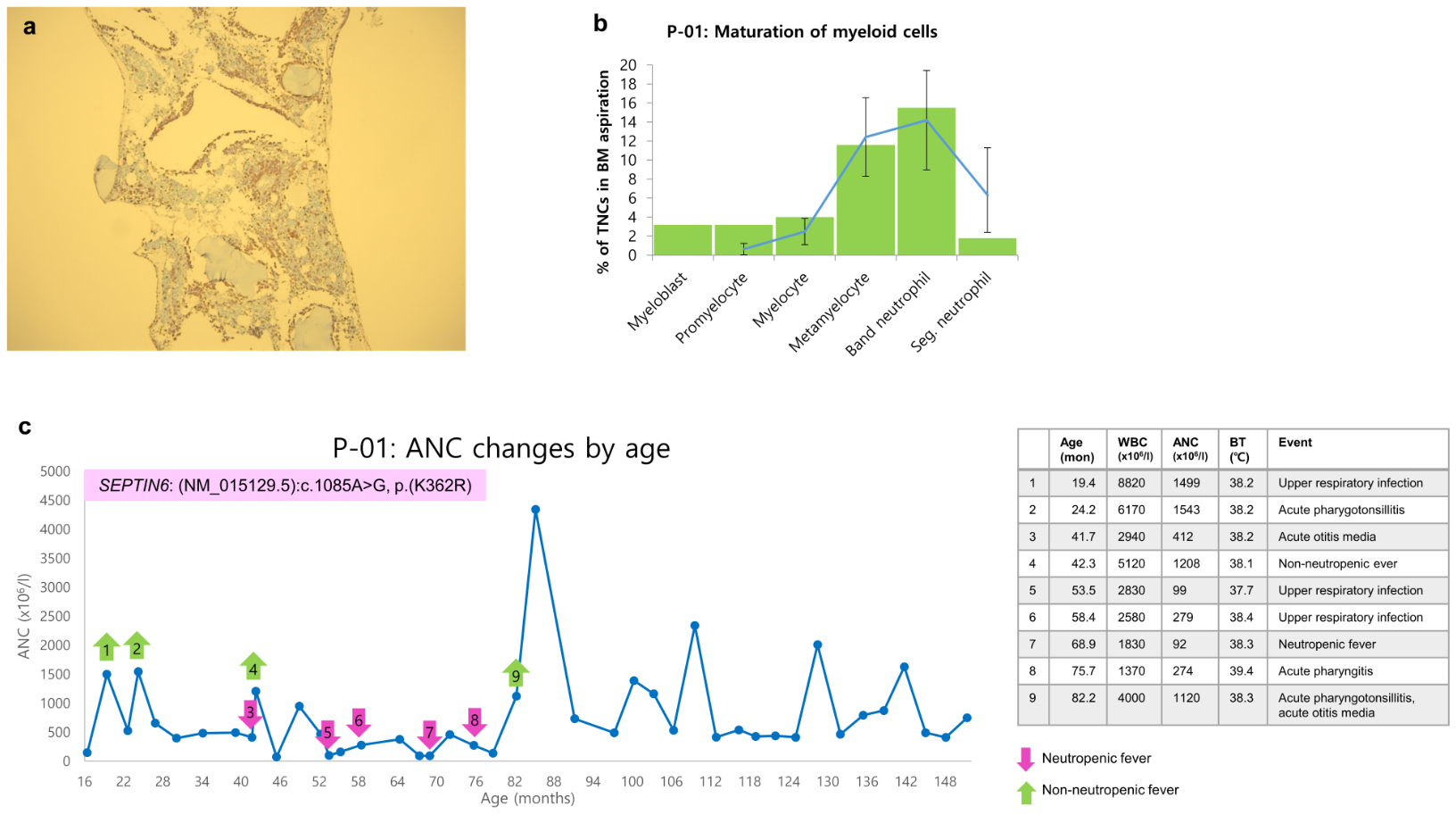


**Figure S8. BM features and clinical course of the patient (P-01) who showed chronic idiopathic neutropenia with a *SEPTIN6* variant of unknown significance. (**a) BM with 30.1% of MPO-positive cells and MPO grade 1. (b) Maturation arrest at the band stage. (c) Clinical course of the patient (P-01). (a) MPO stain, ×100. ANC, absolute neutrophil count; BM, bone marrow; BT, body temperature; MPO, myeloperoxidase WBC, white blood cell.


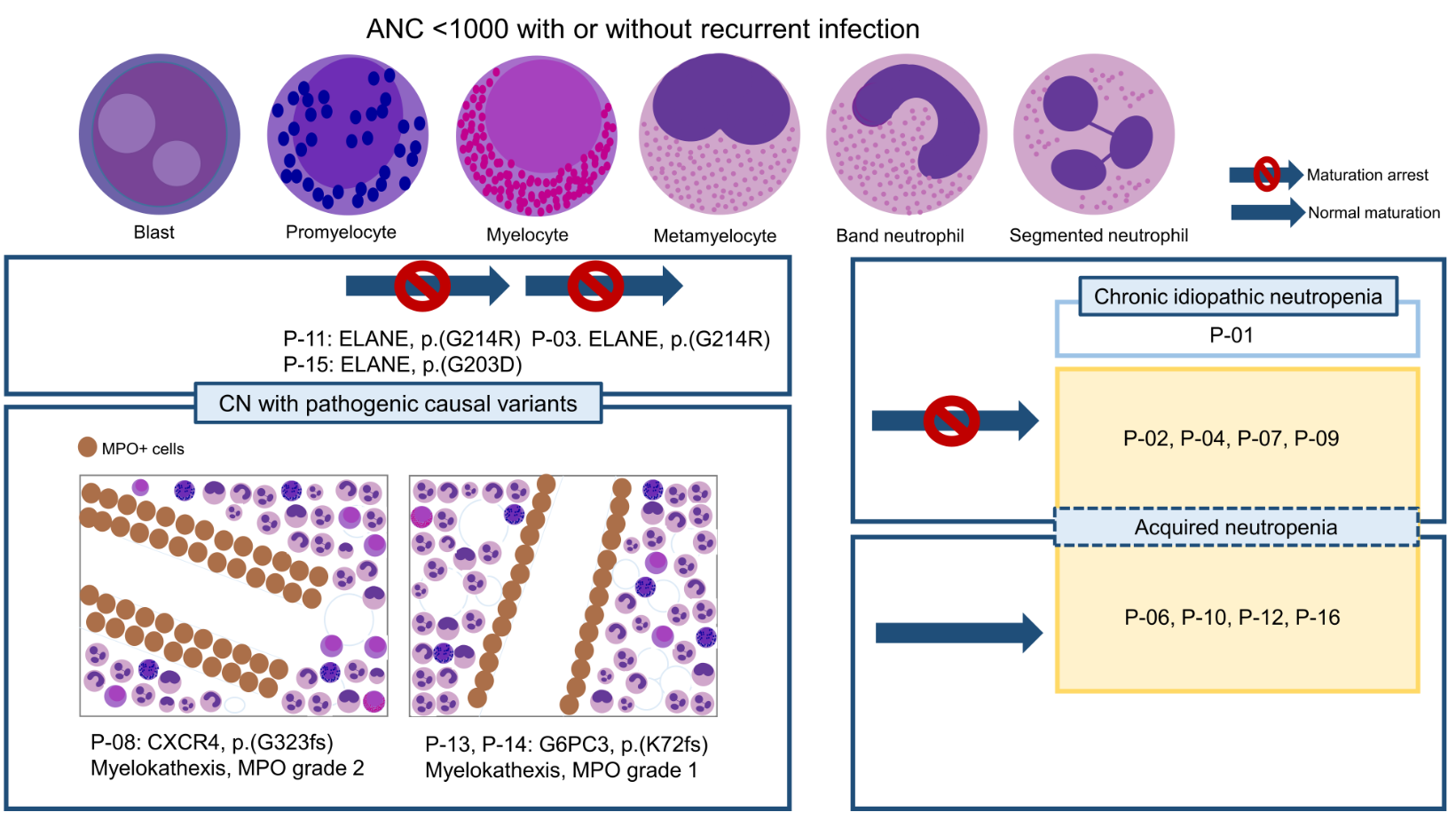


**Figure S9. Genotype–BM histology correlations in 16 neutropenia patients.** Early maturation arrest at the promyelocyte or myelocyte stage was characteristic for *ELANE* mutations. The *CXCR4* mutation was associated with myelokathexis with MPO grade 2, whereas the *G6PC3* mutations were associated with bone marrow retention with MPO grade 1. Maturation block at the band neutrophil stage was observed in one patient with chronic idiopathic neutropenia and 4 acquired neutropenia patients. Other 4 patients showed normal maturation with no myelokathexis. BM, bone marrow.


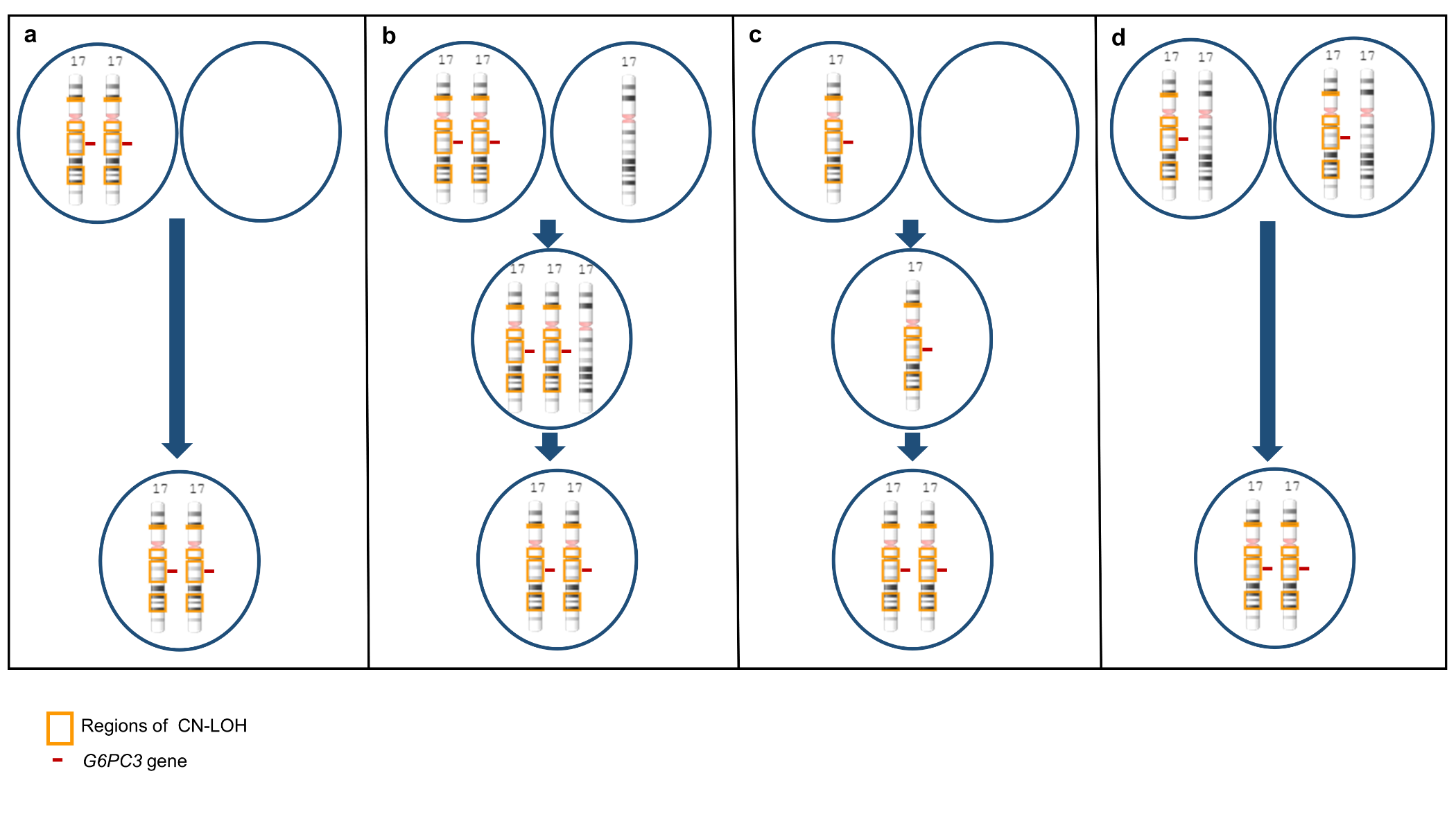


**Figure S10.** **Four hypotheses for the occurrence of homozygous *G6PC3* mutations by CN-LOH.** Four possible alternative scenarios are shown. (A) One gamete has two copies of chromosome 17 with copy-neutral loss of heterozygosity (CN-LOH) in the 17q21.31 region, while the other gamete has no copies. A zygote with the two copies from one gamete (whole uniparental isodisomy of chromosome 17) is generated. (B) One gamete with two copies of chromosome 17 with CN-LOH at 17q21.31 and the other gamete with one copy of chromosome 17 without CN-LOH at 17q21.31 are fertilized and trisomy rescue occurs, leading to a zygote with two copies of chromosome 17 from one gamete (whole uniparental isodisomy of chromosome 17). (C) A gamete with one copy of chromosome 17 with CN-LOH at 17q21.31 fertilizes another gamete with no copies of chromosome 17. Following monosomy rescue, a zygote with two copies of chromosome 17 from one gamete (whole uniparental isodisomy of chromosome 17) is formed. (D) Two gametes can have the same chromosome 17 with CN-LOH at 17q21.31. With a probability of 25%, a zygote with two copies of chromosome 17 with CN-LOH at 17q21.31 is generated. We estimated that the chances of having chromosome 17 with the same CN-LOH regions in both parents are extremely low unless they are consanguineous, which was tentatively ruled out by homozygosity plots, which showed the same CN-LOH regions in chromosome 17 only. CN-LOH, copy-neutral loss of heterozygosity.


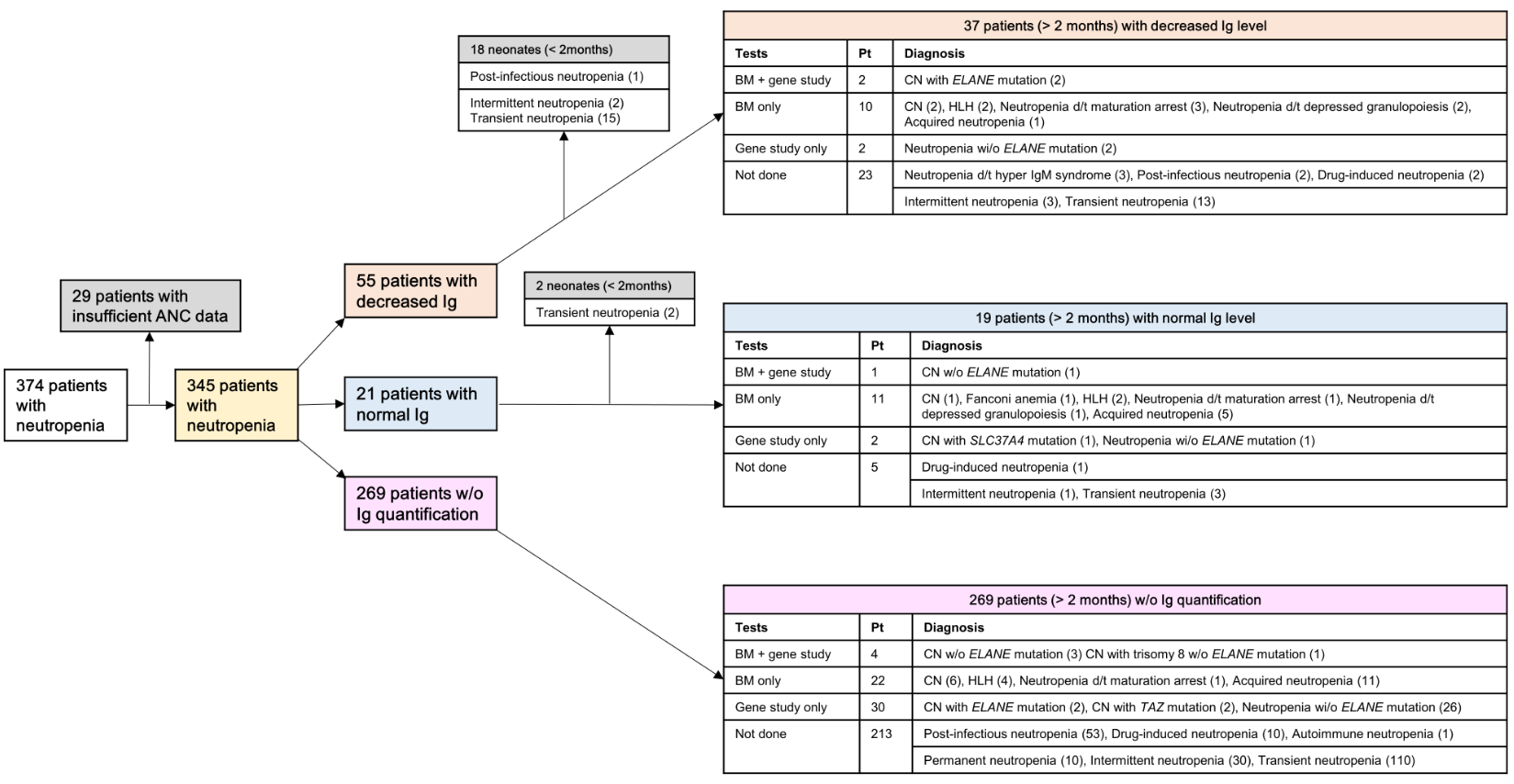


**Figure S11**. **Real-world data on diagnostic work-up algorithms for neutropenia patients in Seoul National University Children’s Hospital from 2009 to 2018.** ANC, absolute neutrophil count; BM, bone marrow; CN, congenital neutropenia; HLH, hemophagocytic lymphohistiocytosis; Ig, immunoglobulin.


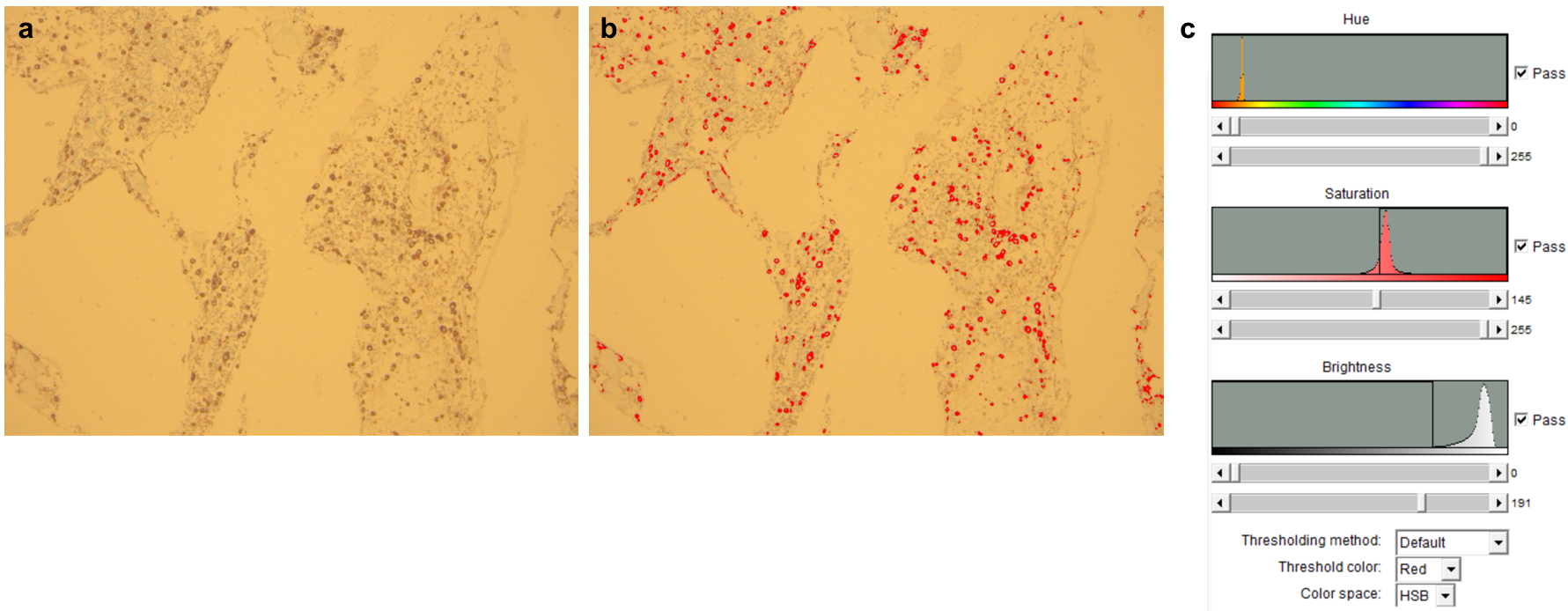


**Figure S12.** **MPO-positive cell count using ImageJ. (**a) MPO-positive cells with brown cytoplasm. (b) MPO-positive cells with red cytoplasm marked using ImageJ (1388 × 1040 pixels). (c) Threshold color setting of hue, saturation and brightness for image analysis using ImageJ. (a-b) MPO stain, ×200. MPO, myeloperoxidase.


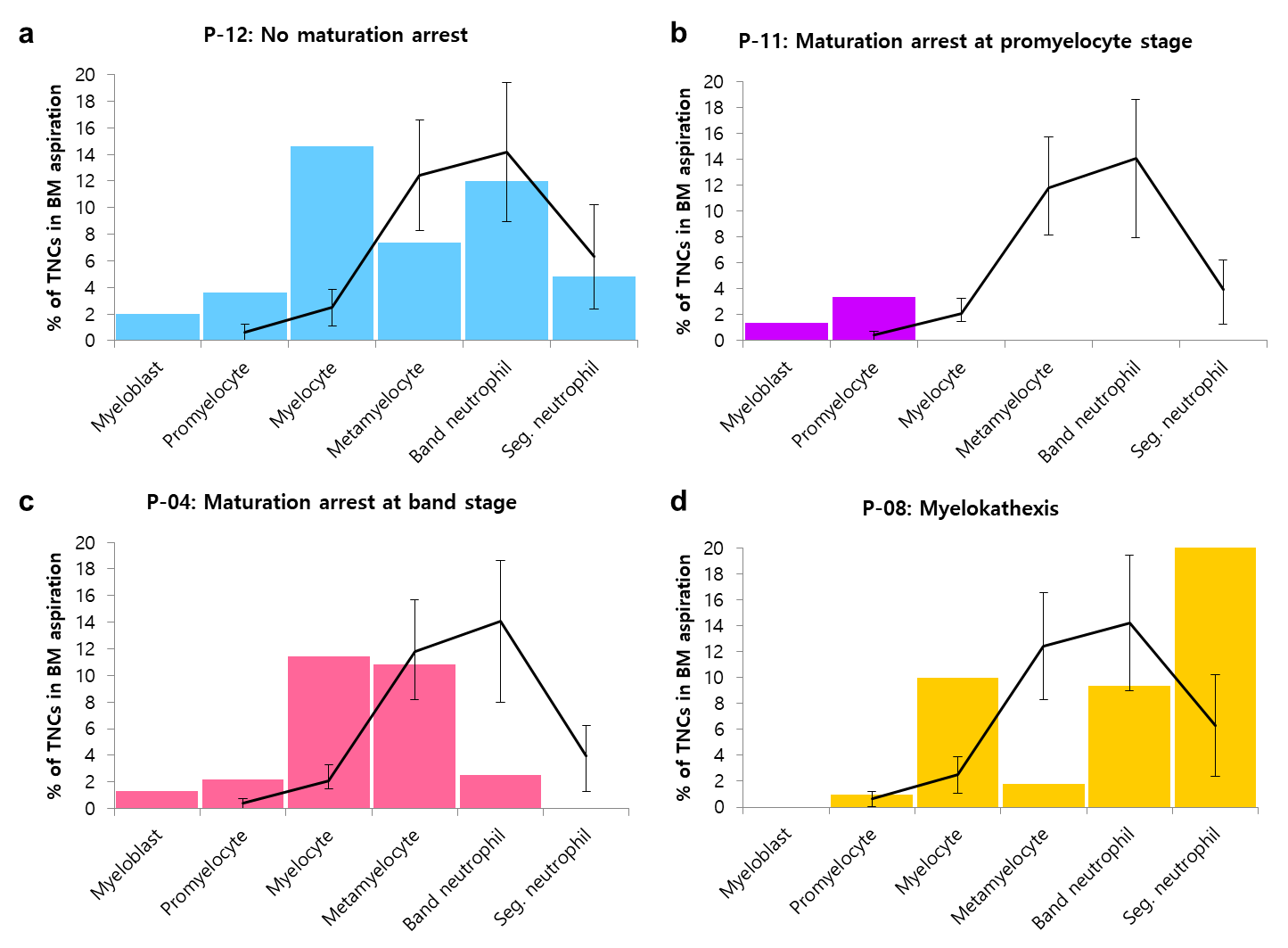


**Figure S13.** **Maturation arrest and myelokathexis assessment according to the pediatric age–specific reference range of BM differential count**. (a) Normal maturation. (b) Maturation arrest at the promyelocyte stage. (c) Maturation arrest at the band stage. (d) Melokathexis. Black broken lines indicate the median value of the reference range; black vertical lines denote the reference range. BM, bone marrow.


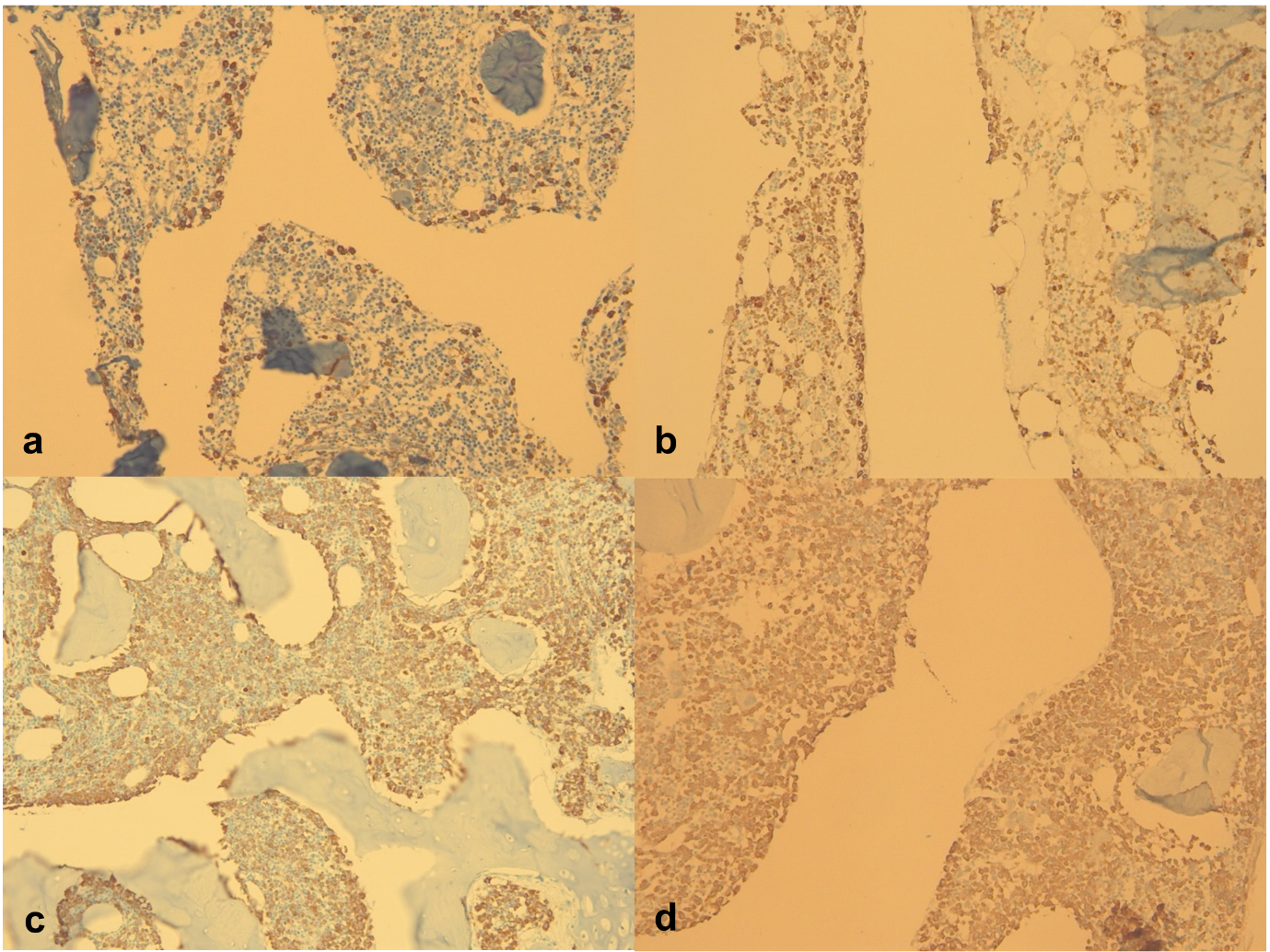


**Figure S14.** **BM section images showing different MPO grades.** (a-d) BM biopsy images with MPO grade 0, 1, 2 and 3. (a-d) MPO stain, ×200. BM, bone marrow; MPO, myeloperoxidase


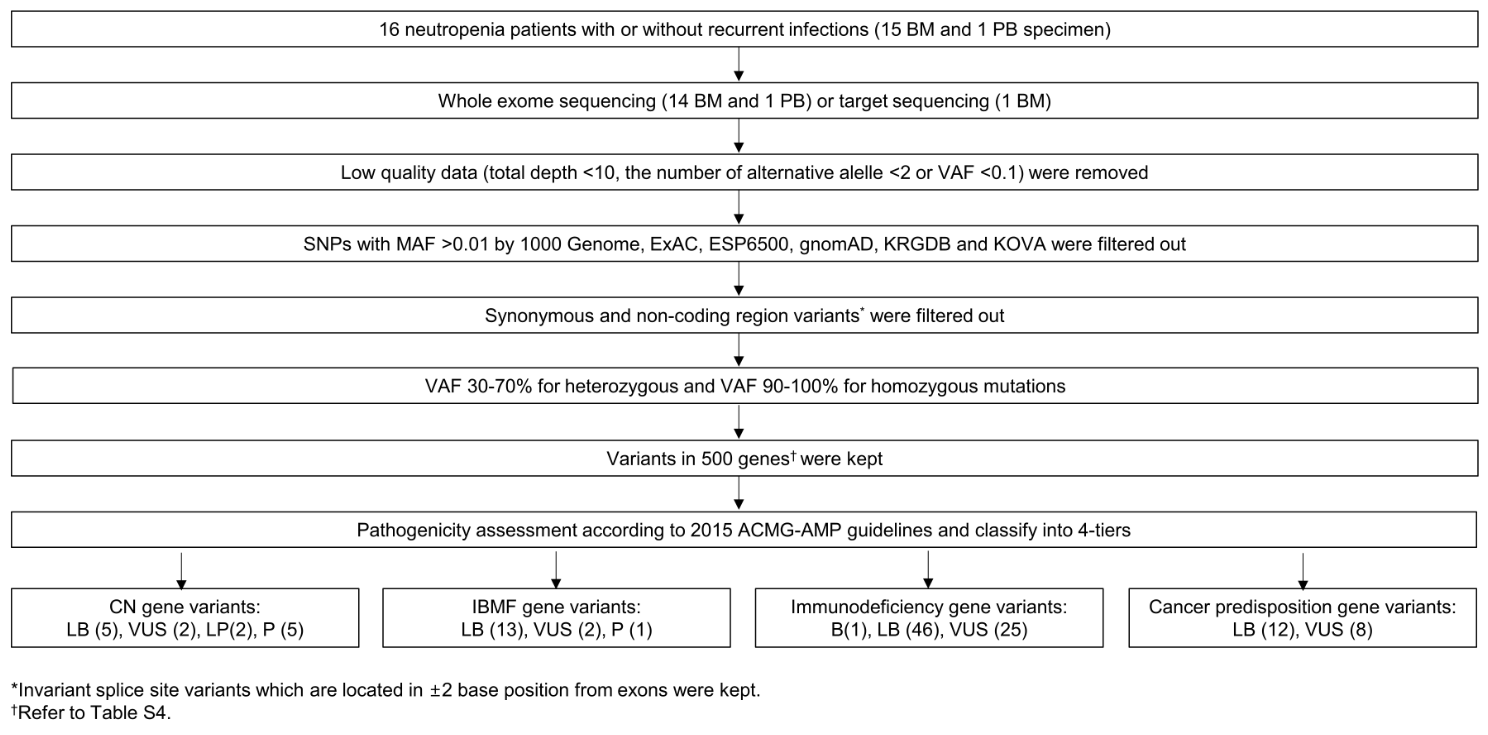
**Figure S15. Strategies for whole-exome or targeted sequencing variant analysis to search for disease-causing variants in 16 neutropenia patients.** B, benign; BM, bone marrow; CN, congenital neutropenia; IBMF, inherited bone marrow failure; LB, likely benign; LP, likely pathogenic; MAF, minor allele frequency; PB, peripheral blood; SNP, single nucleotide polymorphism; VAF, variant allele frequency; VUS, variant of unknown significance; WES, whole-exome sequencing.


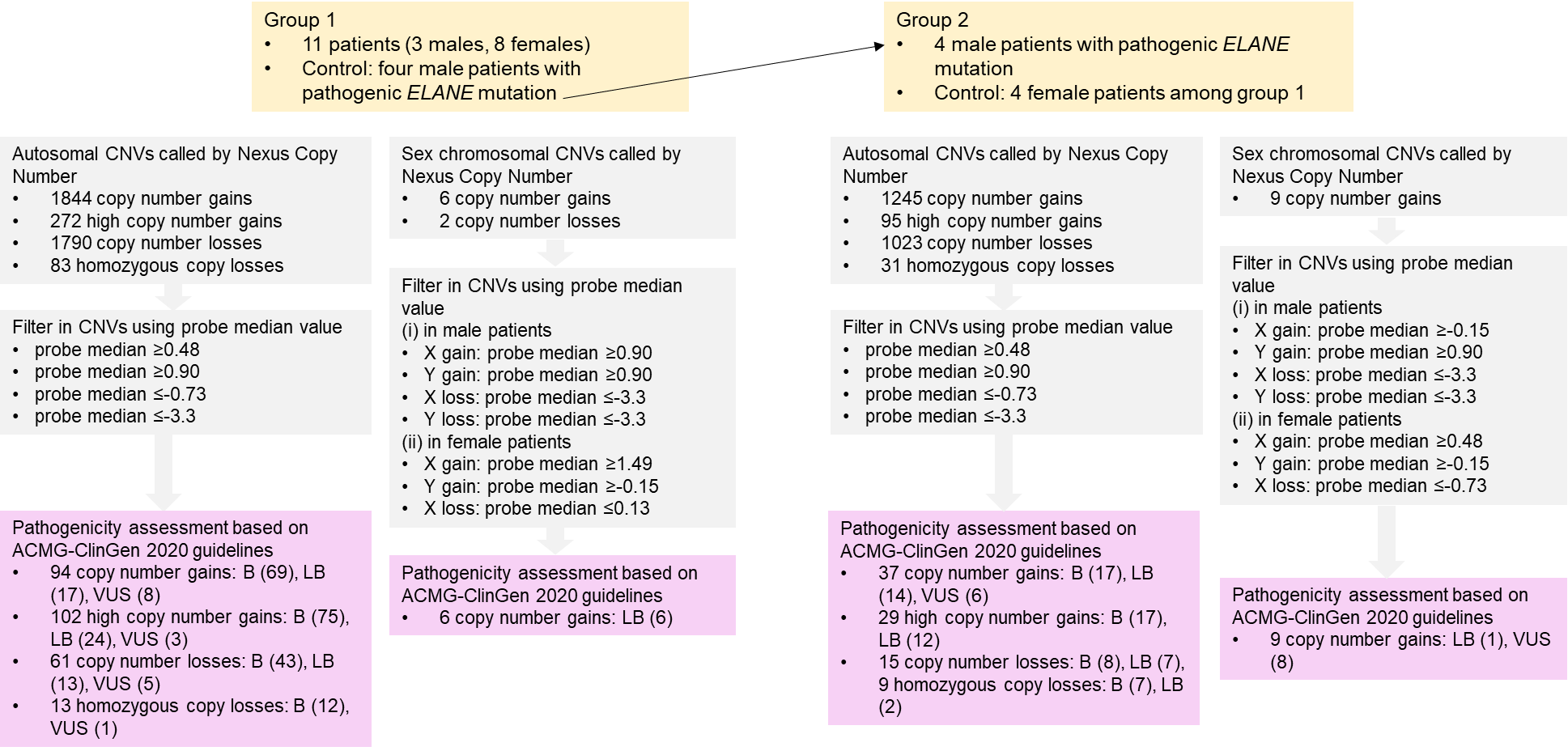


**Figure S16.** **CNV analysis strategies in 15 congenital neutropenia patients who underwent WES.** B, benign; CNV, copy-number variant; LB, likely benign; LP, likely pathogenic; VUS, variant of unknown significance; WES, whole-exome sequencing.

**Table S1.** Variants detected in the 16 neutropenia patients including VUS

| P | CN/IBMF/ID gene analysis | | | |  |  | Cancer predisposition gene analysis | | | |
| --- | --- | --- | --- | --- | --- | --- | --- | --- | --- | --- |
|  | Variants | VAF | Pathogenicity | Pathogenicity assessment^*^ |  | Variants | | VAF | Pathogenicity | Pathogenicity assessment^*^ |
| P-01 | *SEPTIN6* (NM_015129.5):c.1085A>G, p.(K362R) | 0.48 | VUS | PM2 |  | *DHX34* (NM_014681.5):c.1831G>A, p.(A611T)) | | 0.37 | VUS | PM2 |
|  |  |  |  |  |  | *KDM1A* (NM_001009999.2):c.44C>T, p.(A15V) | | 0.58 | VUS | PM2 |
| P-02 | *LRBA* (NM_006726.3):c.3778G>C, p.(A1260P) | 0.51 | VUS | PM2+BP1 |  | *ANKRD26* (NM_014915.2):c.3086A>T, p.(E1029V) | | 0.49 | VUS | . |
|  |  |  |  |  |  | *ERCC6* (NM_000124.2:c.2996A>G, p.(N999S) | | 0.55 | VUS | PM1+PM2+BP1 |
| P-03 | *ELANE* (NM_001972.2):c.452G>A, p.(C151Y) | 0.60 | Likely pathogenic | PM1+PM2+PM5+PP2 +PP3+PP5+PP4 |  | None | |  |  |  |
| P-04 | *IL12RB1* (NM_005535.1):c.1601C>T, p.(P534L) | 0.49 | VUS | PM2 |  | None | |  |  |  |
| P-05 | *ELANE* (NM_001972.2):c.640G>A, p.(G214R) | 0.48 | Pathogenic | PS1+PM1+PM2+PM +PP2+PP3+PP5+PP4 |  | None | |  |  |  |
|  | *SLC7A7* (NM_001126106.1):c.333T>G, p.(F111L) | 0.53 | VUS | PM2 |  |  | |  |  |  |
| P-06 | *FANCI* (NM_001113378.1):c.3568A>G, p.(I1190V) | 0.46 | VUS | PM2 |  | None | |  |  |  |
|  | *C6* (NM_000065.2):c.449G>A, p.(R150H) | 0.66 | VUS | PM2+PP3+BP1 |  |  | |  |  |  |
|  | *CFP* (NM_002621.2):c.1366C>G, p.(L456V) | 0.49 | VUS | PM2+BP1 |  |  | |  |  |  |
|  | *TCF3* (NM_003200.2):c.1069G>A, p.(V357M) | 0.49 | VUS | PM2+BP1 |  |  | |  |  |  |
| P-07 | *LYST* (NM_000081.2):c.5480G>A, p.(C1827Y) | 0.46 | VUS | PM2+PP3+BP1 |  | None | |  |  |  |
|  | *SMARCAL1* (NM_014140.3):c.1786G>A, p.(A596T) | 0.44 | VUS | PM2 |  |  | |  |  |  |
|  | *LIG1* (NM_000234.1):c.1879C>T, p.(R627W) | 0.48 | VUS | PM2+BP1 |  |  | |  |  |  |
| P-08 | *CXCR4* (NM_003467.2):c.966_967del, p.(G323fs) | 0.50 | Pathogenic | PVS1+PM2+PP4 |  | None | |  |  |  |
| P-09 | *LRBA* (NM_006726.3):c.1408A>T, p.(I470F) | 0.53 | VUS | PM2+BP1 |  | *BARD1* (NM_000465.2):c.1862T>C, p.(M621T) | | 0.48 | VUS | PM2+PP3+BP1 |
| P-10 | *MAGT1* (NM_032121.5):c.572G>A, p.(R191Q) | 0.52 | VUS | PM2+BP1 |  | None | |  |  |  |
|  | *NCF4* (NM_013416.3):c.457C>T, p.(R153C) | 0.57 | VUS | PM2+BP1 |  |  | |  |  |  |
|  | *RNASEH2C* (NM_032193.3):c.270G>C, p.(K90N) | 0.49 | VUS | PM2+BP4 |  |  | |  |  |  |
|  | *DNASE2* (NM_001375.2):c.319G>A, p.(D107N) | 0.55 | VUS | PM2+PP3 |  |  | |  |  |  |
| P-11 | *ELANE* (NM_001972.2):c.640G>A, p.(G214R) | 0.52 | Pathogenic | PS1+PM1+PM2+PM5  +PP2+PP3+PP5+PP4 |  | None | |  |  |  |
|  | *SLC7A7* (NM_001126106).1:c.333T>G, p.(F111L) | 0.51 | VUS | PM2+PP3+BP5 |  |  | |  |  |  |
| P-12 | *FANCI* (NM_001113378.1):c.3172G>T, p.(E1058X) | 0.40 | Pathogenic | PVS1+PM2+PP3 |  | None | |  |  |  |
|  | *DOCK2* (NM_004946.2):c.5017C>T, p.(P1673S) | 0.41 | VUS | PM2+BP1 |  |  | |  |  |  |
|  | *EPG5* (NM_020964.2):c.7736G>A, p.(R2579Q) | 0.56 | VUS | PM2+BP1 |  |  | |  |  |  |
|  | *FANCG* (NM_004629.1):c.70G>A, p.(V24I) | 0.46 | VUS | PM2+BP1+PP3 |  |  | |  |  |  |
|  | *SPINK5* (NM_006846.3):c.775G>C, p.(A259P) | 0.46 | VUS | PM2+BP1 |  |  | |  |  |  |
|  | *IRAK1* (NM_001569.3):c.609T>G, p.(C203W) | 0.31 | VUS | PM2 |  |  | |  |  |  |
|  | *TGFBR2* (NM_003242.5):c.1013C>T, p.(T338M) | 0.44 | VUS | PM2+PP2 |  |  | |  |  |  |
| P-13 | *G6PC3* (NM_138387.3):c.214delA, p.(K72fs) | 1.00 | Pathogenic | PVS1+PM2+PP4 |  | None | |  |  |  |
| P-14 | *G6PC3* (NM_138387.3):c.214delA, p.(K72fs) | 1.00 | Pathogenic | PVS1+PM2+PP4 |  | None | |  |  |  |
| P-15 | *ELANE* (NM_001972.2:c.608G>A), p.(G203D) | 0.53 | Likely pathogenic | PM1+PM2+PM5+PP2 +PP3+PP5+PP4 |  | *MST1R* (NM_002447.2):c.1729delC, p.(H577fs) | | 0.48 | VUS | PM2 |
|  | *NFE2L2* (NM_006164.3):c.76C>T, p.(R42X) | 0.36 | VUS | PM2+BP5 |  | *FH* (NM_000143.3):c.1434T>A, p.(N478K) | | 0.48 | VUS | PM2 |
|  |  |  |  |  |  | *SMARCA4* (NM_001128849.1):c.4925A>C, p.(K1642T) | | 0.62 | VUS | PM2 |
| P-16 | *CD3G* (NM_000073.2):c.56-1G>A | 0.44 | VUS | PVS1 |  | None | |  |  |  |
|  | *CD79B* (NM_000626.2):c.97G>C, p.(E33Q) | 0.52 | VUS | PM2+BP4 |  |  | |  |  |  |
|  | *NLRP3* (NM_004895.4):c.200C>G, p.(A67G) | 0.38 | VUS | PP2 |  |  | |  |  |  |
|  | *NSMCE3* (NM_138704.4):c.342C>A, p.(H114Q) | 0.45 | VUS | PM2 |  |  | |  |  |  |
|  | *SKIV2L* (NM_006929.5):c.151G>A, p.(A51T) | 0.45 | VUS | PM2+BP1 |  |  | |  |  |  |

^*^Pathogenicity of each variant was assessed according to 2015 ACMG-AMP guidelines for the interpretation of sequence variants [33].

Abbreviations: CN, congenital neutropenia; IBMF, inherited bone marrow failure; ID, immunodeficiency; LP, likely pathogenic; P, patient; VAF, variant allele frequency; VUS, variant of unknown significance.

**Table S2.** CNV analysis of 15 patients for whom WES was performed

| Patient | Sex | Control | Chromosome region | Cytoband | Event | Length | Probe median | Pathogenicity | Pathogenicity assessment^‡^ |
| --- | --- | --- | --- | --- | --- | --- | --- | --- | --- |
| P-02 | F | M^*^ | chr15:76,678,281-77,271,846 | q24.3 | CN gain | 593,566 | 0.48340754 | VUS | 1A+3B |
| P-02 | F | M^*^ | chr17:58,260,605-59,433,505 | q23.1 - q23.2 | CN gain | 1,172,901 | 0.48906463 | VUS | 1A+2J+3A |
| P-02 | F | M^*^ | chr7:100,494,267-100,624,831 | q22.1 | High copy gain | 130,565 | 1.18507564 | VUS | 1A+2J+3A |
| P-02 | F | M^*^ | chr17:62,385,805-62,486,400 | q23.3 | High copy gain | 100,596 | 0.92298582 | VUS | 1A+3A |
| P-03 | M | F^†^ | chr17:12,872,505-13,262,605 | p12 | CN gain | 390,101 | 0.49955885 | VUS | 1A+2J |
| P-03 | M | F^†^ | chrY:12,500,000-16,936,081 | q11.1 - q11.221 | CN gain | 4,436,082 | 1.13695192 | VUS | 1A+2L+3B |
| P-03 | M | F^†^ | chrY:2,655,180-10,037,833 | p11.31 - p11.2 | CN gain | 7,382,654 | 1.29101992 | VUS | 1A+2L+3A |
| P-04 | F | M^*^ | chr9:133,048,608-133,240,215 | q34.11 | CN gain | 191,608 | 0.49118426 | VUS | 1A+2L |
| P-04 | F | M^*^ | chr11:1,151,752-1,221,908 | p15.5 | CN gain | 70,157 | 0.54887050 | VUS | 1A+3A |
| P-04 | F | M^*^ | chr12:7,172,497-7,260,947 | p13.31 | CN gain | 88,451 | 0.50153291 | VUS | 1A+3A |
| P-05 | M | F^†^ | chr17:45,186,449-45,287,005 | p11.31 - p11.2 | CN gain | 100,557 | 0.52129602 | VUS | 1A+2L |
| P-05 | M | F^†^ | chrY:2,655,180-10,037,833 | p21.1 | CN gain | 7,382,654 | 1.33612883 | VUS | 1A+2L+3A |
| P-06 | F | M^*^ | chr1:104,162,210-104,469,210 | p21.1 | CN loss | 307,001 | -0.85330367 | VUS | 1A+3A |
| P-09 | F | M^*^ | chr7:100,549,731-100,691,231 | q22.1 | CN gain | 141,501 | 0.52355498 | VUS | 1A+2L+3A |
| P-09 | F | M^*^ | chr12:0-176,247 | p13.33 | CN loss | 176,248 | -0.86360770 | VUS | 1A+2C-1+4N |
| P-09 | F | M^*^ | chr11:1,263,708-1,273,708 | p15.5 | High copy gain | 10,001 | 1.31133997 | VUS | 2I |
| P-10 | F | M^*^ | chr1:104,166,710-104,616,110 | p21.1 | CN gain | 449,401 | 0.52691755 | VUS | 1A+2L+2G+3A |
| P-10 | F | M^*^ | chr3:196,510,115-196,554,315 | q29 | CN loss | 44,201 | -0.77570057 | VUS | 2E |
| P-10 | F | M^*^ | chr12:0-187,947 | p13.33 | CN loss | 187,948 | -0.82084373 | VUS | 1A+2C-1+4N |
| P-10 | F | M^*^ | chr12:9,626,047-9,751,283 | p13.31 | Homozygous copy loss | 125,237 | -3.49378848 | VUS | 1A+3A+4D |
| P-11 | M | F^†^ | chr6:133,562,733-133,849,149 | q23.2 | CN gain | 286,417 | 0.51672930 | VUS | 1A+2L+3A |
| P-11 | M | F^†^ | chr8:113,237,828-114,736,511 | q23.3 | CN gain | 1,498,684 | 0.48284447 | VUS | 1A+2J |
| P-11 | M | F^†^ | chrY:2,655,180-10,037,833 | p11.31 - p11.2 | CN gain | 7,382,654 | 0.95616442 | VUS | 1A+2L+3A |
| P-11 | M | F^†^ | chrY:2,655,180-10,037,833 | q11.1 - q11.221 | CN gain | 4,436,082 | 1.16044438 | VUS | 1A+2L+3B |
| P-12 | M | M^*^ | chr12:7,982,347-8,205,185 | p13.31 | CN loss | 222,839 | -0.84832293 | VUS | 1A+2C-1+4N |
| P-13 | M | M^*^ | chr17:45,287,005-48,276,944 | q21.32 - q21.33 | CN-LOH | 2,989,940 | -0.05550943 | N/A | N/A |
| P-14 | M | M^*^ | chr17:44,771,405-48,276,944 | q21.31 - q21.33 | CN-LOH | 3,505,540 | -0.06042904 | N/A | N/A |
| P-14 | M | M^*^ | chr1:104,120,510-104,469,210 | p21.1 | CN gain | 348,701 | 0.50930575 | VUS | 1A+2G+2L+3A |
| P-15 | M | F^†^ | chr5:92,920,830-92,929,730 | q15 | CN gain | 8,901 | 0.52647978 | VUS | 1A+2L+3A+2L |
| P-15 | M | F^†^ | chr15:96,831,446-96,880,946 | q26.2 | CN gain | 49,501 | 0.49010783 | VUS | 1A+2J |
| P-15 | M | F^†^ | chrX:155,001,830-155,270,560 | q28 | CN gain | 268,731 | 0.18132728 | VUS | 1A+2L |
| P-15 | M | F^†^ | chrY:12,500,000-16,936,081 | q11.1 - q11.221 | CN gain | 4,436,082 | 0.92853528 | VUS | 1A+2L+3B |
| P-15 | M | F^†^ | chrY:2,655,180-10,037,833 | p11.31 - p11.2 | CN gain | 7,382,654 | 1.395358 | VUS | 3A+1A+2L |

^*^Four male patients with likely pathogenic or pathogenic *ELANE* variants were used as controls for the copy-number variant analysis.

^†^Four female patients without likely pathogenic or pathogenic variants who were re-diagnosed with autoimmune neutropenia were used as controls for the copy-number variant analysis.

^‡^Pathogenicity of each copy-number variant was assessed according to 2020 ACMG-ClinGen guidelines for the interpretation and reporting of constitutional copy-number variants [34].

Abbreviations: CN, copy number; CNV, copy number variant; N/A, not applicable; VUS, variant of unknown significance; WES, whole-exome sequencing.

**Table S3.** Enumeration of MPO-positive cells on bone marrow section using ImageJ

| Patient | Image 1 | | |  | Image 2 | | |  | Image 3 | | |  | Total images | | |
| --- | --- | --- | --- | --- | --- | --- | --- | --- | --- | --- | --- | --- | --- | --- | --- |
|  | MPO+ cells | TNCs | MPO+ cells (%) |  | MPO+ cells | TNCs | MPO+ cells (%) |  | MPO+ cells | TNCs | MPO+ cells (%) |  | MPO+ cells | TNCs | MPO+ cells (%) |
| P-01 | 911 | 2431 | 37.5 |  | 674 | 2489 | 27.1 |  | 993 | 3640 | 27.3 |  | 2578 | 8560 | 30.1 |
| P-02 | 1289 | 3377 | 38.2 |  | 1696 | 3820 | 44.4 |  | 2337 | 4316 | 54.2 |  | 5322 | 11513 | 46.2 |
| P-03^*^ | N/A | N/A | N/A |  | N/A | N/A | N/A |  | N/A | N/A | N/A |  | N/A | N/A | N/A |
| P-04 | 1821 | 3244 | 56.1 |  | 1477 | 3144 | 47.0 |  | NT | NT | NT |  | 3298 | 6388 | 51.6 |
| P-05 | 352 | 3616 | 9.7 |  | 523 | 3334 | 15.7 |  | NT | NT | NT |  | 875 | 6950 | 12.6 |
| P-06 | 1326 | 3412 | 38.9 |  | 881 | 2620 | 33.6 |  | 912 | 2070 | 44.1 |  | 3119 | 8102 | 38.5 |
| P-07 | 2214 | 5185 | 42.7 |  | 1768 | 3166 | 55.8 |  | NT | NT | NT |  | 3982 | 8351 | 47.7 |
| P-08 | 1184 | 2397 | 49.4 |  | 1687 | 4076 | 41.4 |  | NT | NT | NT |  | 2871 | 6473 | 44.4 |
| P-09 | 1518 | 2647 | 57.4 |  | 1054 | 1722 | 61.2 |  | NT | NT | NT |  | 2572 | 4369 | 58.9 |
| P-10 | 275 | 1692 | 16.3 |  | 316 | 1308 | 24.2 |  | 382 | 1831 | 20.9 |  | 973 | 4831 | 20.1 |
| P-11 | 982 | 1665 | 59.0 |  | 1345 | 3447 | 39.0 |  | NT | NT | NT |  | 2327 | 5112 | 45.5 |
| P-12 | 1639 | 3423 | 47.9 |  | 1039 | 3431 | 30.3 |  | NT | NT | NT |  | 2678 | 6854 | 39.1 |
| P-13 | 1794 | 3921 | 45.8 |  | 1383 | 2905 | 47.6 |  | NT | NT | NT |  | 3177 | 6826 | 46.5 |
| P-14 | 1702 | 4178 | 40.7 |  | 549 | 1450 | 37.9 |  | 764 | 1834 | 41.7 |  | 3015 | 7462 | 40.4 |
| P-15 | 600 | 2556 | 23.5 |  | 1498 | 5762 | 26.0 |  | NT | NT | NT |  | 2098 | 8318 | 25.2 |
| P-16^†^ | N/A | N/A | N/A |  | N/A | N/A | N/A |  | N/A | N/A | N/A |  | N/A | N/A | N/A |

**^*^**Paraffin block for MPO immunohistochemical stain was not retrospectively available in P-03.

^†^Bone marrow section quality of P-16 was inadequate.

Abbreviations: MPO, myeloperoxidase; N/A, not applicable; NT, not tested; TNC, total nucleated cell.

**Table S4.** List of 500 genes selected for variant analysis

| **Gene** | **Location** | **Familial syndrome** | **Inheritance** | **Category** | **Reference** | **WHO 2016 classification** | **IUIS 2019 gene classification** | **Targeted sequencing** |
| --- | --- | --- | --- | --- | --- | --- | --- | --- |
| *ACKR1* | 1q23.2 | White blood cell count QTL | AR | CN | PMID: 28553950 | no | no | no |
| *AK2* | 1p35.1 | Reticular dysgenesis | AR | CN | PMID: 28593997 | no | yes | yes |
| *AP3B1* | 5q14.1 | Hermansky-Pudlak Syndrome | AR | CN | PMID: 28593997 | no | yes | yes |
| *CD40LG* | Xq26.3 | Immunodeficiency, X-linked, with hyper-IgM | XR | CN | PMID: 28593997 | no | yes | yes |
| *CLPB* | 11q13.4 | 3-methylglutaconic aciduria, type VII, with cataracts, neurologic involvement and neutropenia | AR | CN | PMID: 28593997 | no | yes | no |
| *CSF3R* | 1p34.3 | Neutropenia, severe congenital, 7, autosomal recessive | AR | CN | PMID: 21595885 | yes | yes | yes |
| *CXCR2* | 2q35 | Severe congenital neutropenia | AR | CN | PMID: 28593997 | no | no | no |
| *CXCR4* | 2q22.1 | WHIM syndrome | AD | CN | PMID: 21595885 | no | yes | yes |
| *EIF2AK3* | 2p11.2 | Wolcott-Rallison syndrome | AR | CN | PMID: 28593997 | no | no | no |
| *ELANE* | 19p13.3 | Neutropenia, severe congenital 1, autosomal dominant, Neutropenia, cyclic | AD | CN | PMID: 21595885 | yes | yes | yes |
| *G6PC3* | 17q21.31 | Neutropenia, severe congenital 4, autosomal recessive | AR | CN | PMID: 21595885 | yes | yes | yes |
| *GATA2* | 3q21.3 | Emberger syndrome, Immunodeficiency 21 | AD | CN | PMID: 28593997 | no | yes | yes |
| *GFI1* | 1p22.1 | Neutropenia, severe congenital 2, autosomal dominant, Neutropenia, nonimmune chronic idiopathic, of adults | AD | CN | PMID: 21595885 | yes | yes | yes |
| *HAX1* | 1q21.3 | Neutropenia, severe congenital 3, autosomal recessive | AR | CN | PMID: 21595885 | yes | yes | yes |
| *JAGN1* | 3p25.3 | Neutropenia, severe congenital, 6, autosomal recessive | AR | CN | PMID: 28593997 | no | yes | no |
| *LAMTOR2* | 1q22 | Immunodeficiency due to defect in MAPBP-interacting protein | AR | CN | PMID: 28593997 | no | yes | yes |
| *LYST* | 1q42.3 | Chediak-Higashi syndrome | AR | CN | PMID: 28593997 | no | yes | yes |
| *RAB27A* | 15q21.3 | Griscelli syndrome, type 2 | AR | CN | PMID: 28593997 | no | yes | yes |
| *RMRP* | 9p13.3 | Cartilage-hair hypoplasia | AR | CN | PMID: 21595885 | no | yes | yes |
| *SEPTIN6* | Xq24 | Severe congenital neutropenia with tetraploidy, Progressive myelodysplasia and cytogenetic aberrations |  | CN | Blood (2018) 132 (Supplement 1): 644. | no | no | no |
| *SLC37A4* | 11q23.3 | Glycogen storage disease Ib | AR | CN | PMID: 21595885 | no | yes | yes |
| *SRP54* | 14q13.2 | Neutropenia, severe congenital, 8, autosomal dominant | AD | CN | PMID: 31953710 | no | yes | no |
| *TAZ* | Xq28 | Barth syndrome | XR | CN | PMID: 21595885 | no | yes | yes |
| *TCIRG1* | 11q13.2 | Osteopetrosis, AR 1 | AR | CN | PMID: 28593997 | no | yes | no |
| *TCN2* | 22q12.2 | Transcobalamin II deficiency | AR | CN | PMID: 28593997 | no | yes | no |
| *USB1* | 16q21 | Poikiloderma with neutropenia | AR | CN | PMID: 28593997 | no | yes | yes |
| *VPS13B* | 8q22.2 | Cohen syndrome | AR | CN | PMID: 21595885 | no | no | yes |
| *VPS45* | 1q21.2 | Neutropenia, severe congenital, 5, AR | AR | CN | PMID: 28593997 | no | yes | yes |
| *WAS* | Xp11.23 | Neutropenia, severe congenital, X-linked | XR | CN | PMID: 21595885 | yes | yes | yes |
| *ACD* | 16q22.1 | Dyskeratosis congenita, autosomal dominant 6, Dyskeratosis congenita, autosomal recessive 7 | AD/AR | IBMF | PMID: 31953710 | no | yes | no |
| *BRCA1* | 17q21.31 | Fanconi anemia type S | AD | IBMF | PMID: 24237972 | no | yes | yes |
| *BRCA2* | 13q13.1 | Fanconi anemia, complementation group D1 | AR | IBMF | PMID: 24237972 | yes | yes | yes |
| *BRIP1* | 17q23.2 | Fanconi anemia, complementation group J | AR | IBMF | PMID: 24237972 | yes | yes | yes |
| *CTC1* | 17p13.1 | Cerebroretinal microangiopathy with calcifications and cysts | AR | IBMF | PMID: 31953710 | yes | yes | yes |
| *DKC1* | Xq28 | Dyskeratosis congenita | XR | IBMF | PMID: 24237972 | yes | yes | yes |
| *DNAJC21* | 5p13.2 | Bone marrow failure syndrome 3 | AR | IBMF | PMID: 31953710 | no | yes | no |
| *EFL1* | 15q25.2 | Shwachman-Diamond syndrome 2 | AR | IBMF | PMID: 31953710 | no | yes | no |
| *ERCC4* | 16p13.12 | Fanconi anemia, complementation group Q | AR | IBMF | PMID: 31953710 | no | yes | yes |
| *ERCC6L2* | 9q22.32 | Bone marrow failure syndrome 2 | AR | IBMF | PMID: 31953710 | no | yes | no |
| *FANCA* | 16q24.3 | Fanconi anemia, complementation group A | AR | IBMF | PMID: 24237972 | yes | yes | yes |
| *FANCB* | Xp22.2 | Fanconi anemia, complementation group B | XR | IBMF | PMID: 24237972 | yes | yes | yes |
| *FANCC* | 9q22.32 | Fanconi anemia, complementation group C | AR | IBMF | PMID: 24237972 | yes | yes | yes |
| *FANCD2* | 3p25.3 | Fanconi anemia, complementation group D2 | AR | IBMF | PMID: 24237972 | yes | yes | yes |
| *FANCE* | 6p21.31 | Fanconi anemia, complementation group E | AR | IBMF | PMID: 24237972 | yes | yes | yes |
| *FANCF* | 11p14.3 | Fanconi anemia, complementation group F |  | IBMF | PMID: 24237972 | yes | yes | yes |
| *FANCG* | 9p13.3 | Fanconi anemia, complementation group G | AR | IBMF | PMID: 24237972 | yes | yes | yes |
| *FANCI* | 15q26.1 | Fanconi anemia, complementation group I | AR | IBMF | PMID: 24237972 | yes | yes | yes |
| *FANCL* | 2p16.1 | Fanconi anemia, complementation group L | AR | IBMF | PMID: 24237972 | yes | yes | yes |
| *FANCM* | 14q21.2 | Fanconi anemia, type M | AR | IBMF | PMID: 24237972 | yes | yes | yes |
| *GATA1* | Xp11.23 | Anemia, X-linked, with/without neutropenia and/or platelet abnormalities | XR | IBMF | PMID: 10700180, 24453067 | yes | no | yes |
| *LIG4* | 13q33.3 | LIG4 syndrome | AR | IBMF | PMID: 11779494 | no | yes | yes |
| *MAD2L2* | 1p36.22 | Fanconi anemia, complementation group V | AR | IBMF | PMID: 31953710 | no | yes | no |
| *MECOM* | 3q26.2 | Radioulnar synostosis with amegakaryocytic thrombocytopenia 2 | AD | IBMF | PMID: 26581901 | no | no | no |
| *NAF1* | 4q32.2 | Dyskeratosis congenita | AD | IBMF | PMID: 28211564 | no | no | no |
| *NHP2* | 5q35.3 | Dyskeratosis congenita, autosomal recessive 2 | AR | IBMF | PMID: 24237972 | yes | yes | yes |
| *NOP10* | 15q14 | Dyskeratosis congenita, autosomal recessive 1 | AR | IBMF | PMID: 17507419 | yes | yes | yes |
| *PALB2* | 16p12.2 | Fanconi Anemia, Complementation Group N |  | IBMF | PMID: 24237972 | yes | yes | yes |
| *PARN* | 16p13.12 | Pulmonary fibrosis and/or bone marrow failure, telomere-related, 4, Dyskeratosis congenita, autosomal recessive 6 | AD/AR | IBMF | PMID: 31953710 | no | yes | no |
| *RAD51* | 15q15.1 | Fanconi anemia, complementation group R | AD | IBMF | PMID: 31953710 | no | yes | no |
| *RAD51C* | 17q22 | Fanconi anemia, complementation group O | AR | IBMF | PMID: 20400963 | yes | yes | yes |
| *RFWD3* | 16q23.1 | Fanconi anemia type W | AR | IBMF | PMID: 31953710 | yes | yes | no |
| *RPL11* | 1p36.11 | Diamond-Blackfan anemia 7 | AD | IBMF | PMID: 24237972 | yes | no | yes |
| *RPL35A* | 3q29 | Diamond-Blackfan anemia 5 | AD | IBMF | PMID: 24237972 | yes | no | yes |
| *RPL5* | 1p22.1 | Diamond-Blackfan anemia 6 | AD | IBMF | PMID: 24237972 | yes | no | yes |
| *RPS10* | Xq28 | X-linked syndromic mental retardation-35 | XR | IBMF | PMID: 24237972 | yes | no | yes |
| *RPS17* | 15q25.2 | Diamond-Blackfan anemia 4 | AD | IBMF | PMID: 24237972 | yes | no | yes |
| *RPS19* | 17q12 | Diamond-Blackfan anemia 1 | AD | IBMF | PMID: 24237972 | yes | no | yes |
| *RPS24* | 10q22.3 | Diamond-blackfan anemia 3 | AD | IBMF | PMID: 24237972 | yes | no | yes |
| *RPS26* | 12q13.2 | Diamond-Blackfan anemia 10 | AD | IBMF | PMID: 24237972 | yes | no | yes |
| *RPS7* | 2p25.3 | Diamond-Blackfan anemia 8 | AD | IBMF | PMID: 24237972 | yes | no | yes |
| *RTEL1* | 20q13.33 | Dyskeratosis congenita, autosomal dominant 4, Dyskeratosis congenita, autosomal recessive 5 | AD/AR | IBMF | PMID: 31953710 | yes | yes | yes |
| *SAMD9* | 7q21.2 | MIRAGE syndrome, Inherited predisposition to myeloid malignancies | AD | IBMF | PMID: 27182967 | no | yes | yes |
| *SAMD9L* | 7q21.2 | Ataxia-pancytopenia syndrome | AD | IBMF | PMID: 31953710 | no | yes | no |
| *SBDS* | 7q11.21 | Shwachman-Diamond syndrome | AR | IBMF | PMID: 21595885 | no | yes | yes |
| *SLX4* | 16p13.3 | Fanconi anemia, complementation group P | AR | IBMF | PMID: 21240277 | yes | yes | yes |
| *SRP72* | 4q12 | Bone marrow failure syndrome 1 | AD | IBMF | PMID: 22541560 | no | yes | yes |
| *STN1* | 10q24.33 | Dyskeratosis congenita | AD | IBMF | PMID: 31953710 | no | yes | no |
| *TERC* | 3q26.2 | Dyskeratosis congenita, autosomal dominant 1 | AD | IBMF | PMID: 24237972 | yes | yes | yes |
| *TERT* | 5p15.33 | Dyskeratosis congenita, autosomal dominant 2, Dyskeratosis congenita, autosomal recessive 4 | AD/AR | IBMF | PMID: 24237972 | yes | yes | yes |
| *TINF2* | 14q12 | Dyskeratosis congenita, autosomal dominant 3, Revesz syndrome | AD | IBMF | PMID: 24237972 | yes | yes | yes |
| *TP53* | 17p13.3 | BMFS5 | AD | IBMF | PMID: 31953710 | no | yes | yes |
| *UBE2T* | 1q32.1 | Fanconi anemia, complementation group T | AR | IBMF | PMID: 31953710 | no | yes | no |
| *WRAP53* | 17p13.1 | Dyskeratosis congenita, autosomal recessive 3 | AR | IBMF | PMID: 31953710 | yes | yes | yes |
| *XRCC2* | 7q36.1 | Fanconi anemia, complementation group U | AR | IBMF | PMID: 31953710 | no | yes | no |
| *ACP5* | 19p13.2 | Spondyloenchondrodysplasia with immune dysregulation | AR | ID | PMID: 31953710 | no | yes | no |
| *ACTB* | 7p22.1 | β actin deficiency | AD | ID | PMID: 31953710 | no | yes | yes |
| *ADA* | 20q13.12 | Severe combined immunodeficiency due to ADA deficiency | AR | ID | PMID: 31953710 | no | yes | yes |
| *ADA2* | 22q11.1 | Vasculitis, autoinflammation, immunodeficiency, and hematologic defects syndrome | AR | ID | PMID: 31953710 | no | yes | no |
| *ADAM17* | 2p25.1 | ADAM17 deficiency | AR | ID | PMID: 31953710 | no | yes | no |
| *ADAR* | 1q21.3 | ADAR1 deficiency, AGS6 | AD | ID | PMID: 31953710 | no | yes | no |
| *AICDA* | 12p13.31 | Immunodeficiency with hyper-IgM, type 2 | AR | ID | PMID: 31953710 | no | yes | no |
| *AIRE* | 21q22.3 | Autoimmune polyendocrinopathy syndrome, type I, with or without reversible metaphyseal dysplasia | AD/AR | ID | PMID: 31953710 | no | yes | no |
| *ALPI* | 2q37.1 | ALPI deficiency | AR | ID | PMID: 31953710 | no | yes | no |
| *AP1S3* | 2q36.1 | AP1S3 deficiency | AR | ID | PMID: 31953710 | no | yes | no |
| *AP3D1* | 19p13.3 | Hermansky-Pudlak syndrome 10 | AR | ID | PMID: 31953710 | no | yes | no |
| *APOL1* | 22q12.3 | Trypanosomiasis | AD | ID | PMID: 31953710 | no | yes | no |
| *ARHGEF1* | 19q13.2 | ARHGEF1 deficiency | AR | ID | PMID: 31953710 | no | yes | no |
| *ARPC1B* | 7q22.1 | Platelet abnormalities with eosinophilia and immune-mediated inflammatory disease | AR | ID | PMID: 31953710 | no | yes | no |
| *ATM* | 11q22.3 | Ataxia-telangiectasia | AR | ID | PMID: 31953710 | no | yes | yes |
| *ATP6AP1* | Xq28 | ATP6AP1 deficiency | XL | ID | PMID: 31953710 | no | yes | no |
| *B2M* | 15q21.1 | MHC class I deficiency | AR | ID | PMID: 31953710 | no | yes | yes |
| *BACH2* | 6q15 | Immunodeficiency 60 | AD | ID | PMID: 31953710 | no | yes | no |
| *BCL10* | 1p22.3 | Immunodeficiency 37 | AR | ID | PMID: 31953710 | no | yes | yes |
| *BCL11B* | 14q32.2 | Immunodeficiency 49 | AD | ID | PMID: 31953710 | no | yes | yes |
| *BLM* | 15q26.1 | Bloom syndrome | AR | ID | PMID: 31953710 | no | yes | yes |
| *BLNK* | 10q24.1 | Agammaglobulinemia 4 | AR | ID | PMID: 31953710 | no | yes | yes |
| *BTK* | Xq22.1 | Agammaglobulinemia, X-linked 1 | XR | ID | PMID: 31953710 | no | yes | yes |
| *C1QA* | 1p36.12 | C1q deficiency | AR | ID | PMID: 31953710 | no | yes | no |
| *C1QB* | 1p36.12 | C1q deficiency | AR | ID | PMID: 31953710 | no | yes | no |
| *C1QC* | 1p36.12 | C1q deficiency | AR | ID | PMID: 31953710 | no | yes | no |
| *C1R* | 12p13.31 | C1r deficiency | AR/AD | ID | PMID: 31953710 | no | yes | no |
| *C1S* | 12p13.31 | C1s deficiency |  | ID | PMID: 31953710 | no | yes | no |
| *C2* | 6p21.33 | C2 deficiency | AR | ID | PMID: 31953710 | no | yes | no |
| *C3* | 19p13.3 | C3 deficiency | AR | ID | PMID: 31953710 | no | yes | no |
| *C4A* | 6p21.33 | C4A deficiency | AR | ID | PMID: 31953710 | no | yes | no |
| *C4B* | 6p21.33 | C4B deficiency |  | ID | PMID: 31953710 | no | yes | no |
| *C5* | 9q33.2 | C5 deficiency | AR | ID | PMID: 31953710 | no | yes | no |
| *C6* | 5p13.1 | C6 deficiency |  | ID | PMID: 31953710 | no | yes | no |
| *C7* | 5p13.1 | C7 deficiency |  | ID | PMID: 31953710 | no | yes | no |
| *C8A* | 1p32.2 | C8α deficiency |  | ID | PMID: 31953710 | no | yes | no |
| *C8B* | 6p21.33 | C8 β deficiency |  | ID | PMID: 31953710 | no | yes | no |
| *C8G* | 9q34.3 | C8 γ deficiency |  | ID | PMID: 31953710 | no | yes | no |
| *C9* | 5p13.1 | C9 deficiency |  | ID | PMID: 31953710 | no | yes | no |
| *CARD11* | 7p22.2 | B-cell expansion with NFKB and T-cell anergy, Immunodeficiency 11A, Immunodeficiency 11B with atopic dermatitis | AD/AR | ID | PMID: 31953710 | no | yes | yes |
| *CARD14* | 17q25.3 | CAMPS (CARD14 mediated psoriasis) | AD | ID | PMID: 31953710 | no | yes | no |
| *CARD9* | 9q34.3 | Candidiasis, familial, 2, autosomal recessive | AR | ID | PMID: 31953710 | no | yes | no |
| *CARMIL2* | 16q22.1 | Immunodeficiency 58 | AR | ID | PMID: 31953710 | no | yes | no |
| *CASP10* | 2q33.1 | Autoimmune lymphoproliferative syndrome | AD | ID | PMID: 31953710 | no | yes | yes |
| *CASP8* | 2q33.1 | Autoimmune lymphoproliferative syndrome, type IIB | AR | ID | PMID: 31953710 | no | yes | yes |
| *CCBE1* | 18q21.32 | Hennekam-lymphangiectasia-lymphedema syndrome | AR | ID | PMID: 31953710 | no | yes | no |
| *CD19* | 16p11.2 | Immunodeficiency, common variable, 3 | AR | ID | PMID: 31953710 | no | yes | no |
| *CD247* | 1q24.2 | Immunodeficiency 25 | AR | ID | PMID: 31953710 | no | yes | no |
| *CD27* | 12p13.31 | Lymphoproliferative syndrome 2 | AR | ID | PMID: 31953710 | no | yes | yes |
| *CD3D* | 11q23.3 | Immunodeficiency 19 | AR | ID | PMID: 31953710 | no | yes | yes |
| *CD3E* | 11q23.3 | Immunodeficiency 18 | AR | ID | PMID: 31953710 | no | yes | yes |
| *CD3G* | 11q23.3 | Immunodeficiency 17, CD3 gamma deficient | AR | ID | PMID: 31953710 | no | yes | no |
| *CD40* | 20q13.12 | Immunodeficiency with Hyper-IgM | AR | ID | PMID: 31953710 | no | yes | no |
| *CD46* | 1q32.2 | Hemolytic uremic syndrome, atypical | AD/AR | ID | PMID: 31953710 | no | yes | no |
| *CD55* | 1q32.2 | Complement hyperactivation, angiopathic thrombosis, and protein-losing enteropathy | AR | ID | PMID: 31953710 | no | yes | no |
| *CD70* | 19p13.3 | Lymphoproliferative syndrome 3 | AR | ID | PMID: 31953710 | no | yes | yes |
| *CD79A* | 19q13.2 | Agammaglobulinemia 3 | AR | ID | PMID: 31953710 | no | yes | yes |
| *CD79B* | 17q23.3 | Agammaglobulinemia 6 | AR | ID | PMID: 31953710 | no | yes | yes |
| *CD81* | 11p15.5 | Immunodeficiency, common variable, 6 | AR | ID | PMID: 31953710 | no | yes | no |
| *CD8A* | 2p11.2 | CD8 deficiency, familial | AR | ID | PMID: 31953710 | no | yes | no |
| *CDCA7* | 2q31.1 | Immunodeficiency-centromeric instability-facial anomalies syndrome 3 | AR | ID | PMID: 31953710 | no | yes | no |
| *CEBPE* | 14q11.2 | Specific granule deficiency | AR | ID | PMID: 31953710 | no | yes | no |
| *CFB* | 6p21.33 | Complement factor B deficiency | AR | ID | PMID: 31953710 | no | yes | no |
| *CFD* | 19p13.3 | Complement factor D deficiency | AR | ID | PMID: 31953710 | no | yes | no |
| *CFH* | 1q31.3 | Complement factor H deficiency | AD/AR | ID | PMID: 31953710 | no | yes | no |
| *CFHR1* | 1q31.3 | Factor H-related protein deficiencies | AD/AR | ID | PMID: 31953710 | no | yes | no |
| *CFHR2* | 1q31.3 | Factor H-related protein deficiencies | AD/AR | ID | PMID: 31953710 | no | yes | no |
| *CFHR3* | 1q31.3 | Factor H-related protein deficiencies | AD/AR | ID | PMID: 31953710 | no | yes | no |
| *CFHR4* | 1q31.3 | Factor H-related protein deficiencies | AD/AR | ID | PMID: 31953710 | no | yes | no |
| *CFHR5* | 1q31.3 | Factor H-related protein deficiencies | AD/AR | ID | PMID: 31953710 | no | yes | no |
| *CFI* | 4q25 | Complement factor I deficiency | AR | ID | PMID: 31953710 | no | yes | no |
| *CFP* | Xp11.23 | Properdin deficiency, X-linked | XR | ID | PMID: 31953710 | no | yes | no |
| *CFTR* | 7q31.2 | Cystic fibrosis | AD/AR | ID | PMID: 31953710 | no | yes | no |
| *CHD7* | 8q12.2 | CHARGE syndrome | AD | ID | PMID: 31953710 | no | yes | no |
| *CIB1* | 15q26.1 | CIB1 deficiency | . | ID | PMID: 31953710 | no | yes | no |
| *CIITA* | 16p13.13 | Bare lymphocyte syndrome, type II, complementation group A | AR | ID | PMID: 31953710 | no | yes | yes |
| *CLCN7* | 16p13.3 | Osteopetrosis, autosomal dominant 2 | AD | ID | PMID: 31953710 | no | yes | no |
| *COLEC11* | 2p25.3 | 3MC syndrome 2 | AR | ID | PMID: 26454309 | no | no | no |
| *COPA* | 1q23.2 | Autoimmune interstitial lung, joint, and kidney disease | AD | ID | PMID: 31953710 | no | yes | no |
| *CORO1A* | 16p11.2 | Immunodeficiency 8 | AR | ID | PMID: 31953710 | no | yes | no |
| *CR2* | 1q32.2 | Immunodeficiency, common variable, 7 | AR | ID | PMID: 31953710 | no | yes | no |
| *CSF2RA* | Xp22.32 | Pulmonary alveolar proteinosis | XL | ID | PMID: 31953710 | no | yes | no |
| *CSFR2B* | 22q12.3 | Pulmonary alveolar proteinosis | AR | ID | PMID: 31953710 | no | yes | no |
| *CTLA4* | 2q33.2 | Autoimmune lymphoproliferative syndrome, type V | AD | ID | PMID: 31953710 | no | yes | no |
| *CTPS1* | 1p34.2 | Immunodeficiency 24 | AR | ID | PMID: 31953710 | no | yes | no |
| *CTSC* | 11q14.2 | Haim-Munk syndrome | AR | ID | PMID: 31953710 | no | yes | no |
| *CYBA* | 16q24.2 | Chronic granulomatous disease, autosomal, due to deficiency of CYBA | AR | ID | PMID: 31953710 | no | yes | no |
| *CYBB* | Xp21.1-p11.4 | Chronic granulomatous disease, X-linked, Immunodeficiency 34, mycobacteriosis, X-linked | XR | ID | PMID: 31953710 | no | yes | no |
| *CYBC1* | 17q25.3 | Autosomal recessive CGD | AR | ID | PMID: 31953710 | no | yes | no |
| *DBR1* | 3q22.3 | DBR1 deficiency | AR | ID | PMID: 31953710 | no | yes | no |
| *DCLRE1C* | 10p13 | Omenn syndrome, Severe combined immunodeficiency, Athabascan type | AR | ID | PMID: 31953710 | no | yes | yes |
| *DEF6* | 6p21.31 | DEF6 deficiency | AR | ID | PMID: 31953710 | no | yes | no |
| *DNASE1L3* | 3p14.3 | Pediatric systemic lupus erythematosus due to DNASE1L3 deficiency | AR | ID | PMID: 31953710 | no | yes | no |
| *DNASE2* | 19p13.13 | DNAse II deficiency | AR | ID | PMID: 31953710 | no | yes | no |
| *DNMT3B* | 20q11.21 | Immunodeficiency-centromeric instability-facial anomalies syndrome 1 | AR | ID | PMID: 31953710 | no | yes | yes |
| *DOCK2* | 5q35.1 | Immunodeficiency 40 | AR | ID | PMID: 31953710 | no | yes | no |
| *DOCK8* | 9p24.3 | Hyper-IgE recurrent infection syndrome, autosomal recessive | AR | ID | PMID: 31953710 | no | yes | no |
| *EPG5* | 18q12.3-q21.1 | Vici syndrome | AR | ID | PMID: 31953710 | no | yes | no |
| *ERBIN* | 5q12.3 | ERBIN deficiency | AD | ID | PMID: 31953710 | no | yes | no |
| *EXTL3* | 8p21.1 | Immunoskeletal dysplasia with neurodevelopmental abnormalities (ISDNA) | AR | ID | PMID: 31953710 | no | yes | no |
| *FAAP24* | 19q13.11 | FAAP24 deficiency | AR | ID | PMID: 31953710 | no | yes | no |
| *FADD* | 11q13.3 | Infections, recurrent, with encephalopathy, hepatic dysfunction, and cardiovascular malformations | AR | ID | PMID: 31953710 | no | yes | no |
| *FAS* | 10q23.31 | Autoimmune lymphoproliferative syndrome, type IA | AD/AR | ID | PMID: 31953710 | no | yes | yes |
| *FASLG* | 1q24.3 | Autoimmune lymphoproliferative syndrome, type IB | AD | ID | Phenotype MIM number: 601859 | no | no | yes |
| *FAT4* | 4q28.1 | Hennekam-lymphangiectasia-lymphedema syndrome | AR | ID | PMID: 31953710 | no | yes | yes |
| *FCGR3A* | 1q23.3 | CD16 deficiency | AR | ID | PMID: 31953710 | no | yes | no |
| *FCHO1* | 19p13.11 | FCHO1 deficiency | AR | ID | PMID: 31953710 | no | yes | no |
| *FCN3* | 1p36.11 | Ficolin 3 deficiency | AR | ID | PMID: 31953710 | no | yes | no |
| *FERMT1* | 20p12.3 | FERMT1 deficiency | AR | ID | PMID: 31953710 | no | yes | no |
| *FERMT3* | 11q13.1 | Leukocyte adhesion deficiency, type III | AR | ID | PMID: 31953710 | no | yes | no |
| *FOXN1* | 17q11.2 | T-cell immunodeficiency, congenital alopecia, and nail dystrophy | AR/AD | ID | PMID: 31953710 | no | yes | yes |
| *FOXP3* | Xp11.23 | Immunodysregulation, polyendocrinopathy, and enteropathy, X-linked | XR | ID | PMID: 31953710 | no | yes | yes |
| *FPR1* | 19q13.41 | Localized juvenile periodontitis | AR | ID | PMID: 31953710 | no | yes | no |
| *G6PD* | Xq28 | G6PD deficiency class I | XL | ID | PMID: 31953710 | no | yes | yes |
| *GINS1* | 20p11.21 | Immunodeficiency 55 | AR | ID | PMID: 31953710 | no | yes | no |
| *HAVCR2* | 5q33.3 | T cell lymphoma subcutaneous panniculitis-like (TIM3 deficiency) | AR | ID | PMID: 31953710 | no | yes | no |
| *HELLS* | 10q23.33 | Immunodeficiency-centromeric instability-facial anomalies syndrome 4 | AR | ID | PMID: 31953710 | no | yes | no |
| *HMOX1* | 22q12.3 | Isolated congenital asplenia (ICA) | AR | ID | PMID: 31953710 | no | yes | no |
| *HYOU1* | 11q23.3 | Immunodeficiency 59 and hypoglycemia | AR | ID | PMID: 31953710 | no | yes | no |
| *ICOS* | 2q33.2 | Immunodeficiency, common variable, 1 | AR | ID | PMID: 31953710 | no | yes | no |
| *ICOSLG* | 21q22.3 | ICOSL deficiency | AR | ID | PMID: 31953710 | no | yes | no |
| *IFIH1* | 2q24.2 | Singleton-Merten syndrome, Aicardi-Goutieres syndrome 7 | AD/AR | ID | PMID: 31953710 | no | yes | no |
| *IFNAR2* | 21q22.11 | Immunodeficiency 45 | AR | ID | PMID: 31953710 | no | yes | no |
| *IFNGR1* | 6q23.3 | Immunodeficiency 27A, mycobacteriosis, AR, Immunodeficiency 27B, mycobacteriosis, AD | AD/AR | ID | PMID: 31953710 | no | yes | no |
| *IFNGR2* | 21q22.11 | Immunodeficiency 28, mycobacteriosis | AR | ID | PMID: 31953710 | no | yes | no |
| *IGHM* | 14q32.33 | μ heavy chain deficiency | AR | ID | PMID: 31953710 | no | yes | no |
| *IGKC* | 2p11.2 | Kappa chain deficiency | AR | ID | PMID: 31953710 | no | yes | no |
| *IGLL1* | 22q11.23 | Agammaglobulinemia 2 | AR | ID | PMID: 31953710 | no | yes | no |
| *IKBKB* | 8p11.21 | Immunodeficiency 15A, Immunodeficiency 15B | AD/AR | ID | PMID: 31953710 | no | yes | no |
| *IKBKG* | Xq28 | EDA-ID due to NEMO/IKBKG deficiency | XL | ID | PMID: 31953710 | no | yes | no |
| *IKZF1* | 7p12.2 | Immunodeficiency, common variable, 13 | AD | ID | PMID: 31953710 | no | yes | yes |
| *IL10* | 1q32.1 | IL-10 deficiency | AR | ID | PMID: 31953710 | no | yes | no |
| *IL10RA* | 11q23.3 | IL-10R deficiency | AR | ID | PMID: 31953710 | no | yes | no |
| *IL10RB* | 21q22.11 | IL-10R deficiency | AR | ID | PMID: 31953710 | no | yes | no |
| *IL12B* | 5q33.3 | Immunodeficiency 29, mycobacteriosis | AR | ID | PMID: 31953710 | no | yes | no |
| *IL12RB1* | 19p13.11 | Immunodeficiency 30 | AR | ID | PMID: 31953710 | no | yes | no |
| *IL12RB2* | 1p31.3 | IL-12Rβ2 deficiency | AR | ID | PMID: 31953710 | no | yes | no |
| *IL17F* | 6p12.2 | IL-17F deficiency | AD | ID | PMID: 31953710 | no | yes | no |
| *IL17RA* | 22q11.1 | Immunodeficiency 51 | AR | ID | PMID: 31953710 | no | yes | no |
| *IL17RC* | 3p25.3 | Candiasis, familial, 9 | AR | ID | PMID: 31953710 | no | yes | no |
| *IL18BP* | 11q13.4 | IL-18BP deficiency | AR | ID | PMID: 31953710 | no | yes | no |
| *IL1RN* | 2q14.1 | Interleukin 1 receptor antagonist deficiency | AR | ID | PMID: 31953710 | no | yes | no |
| *IL21* | 4q27 | Immunodeficiency, common variable, 11 | AR | ID | PMID: 31953710 | no | yes | no |
| *IL21R* | 16p12.1 | Immunodeficiency 56 | AR | ID | PMID: 31953710 | no | yes | no |
| *IL23R* | 1p31.3 | IL-23R deficiency | AR | ID | PMID: 31953710 | no | yes | no |
| *IL2RA* | 10p15.1 | Immunodeficiency 41 with lymphoproliferation and autoimmunity | AR | ID | PMID: 31953710 | no | yes | no |
| *IL2RB* | 22q12.3 | CD122 deficiency | AR | ID | PMID: 31953710 | no | yes | no |
| *IL2RG* | Xq13.1 | Combined immunodeficiency, X-linked, moderate, Severe combined immunodeficiency, X-linked | XL | ID | PMID: 31953710 | no | yes | yes |
| *IL36RN* | 2q14.1 | DITRA (Deficiency of IL-36 receptor antagonist) | AR | ID | PMID: 31953710 | no | yes | no |
| *IL6R* | 1q21.3 | IL6 receptor deficiency | AR | ID | PMID: 31953710 | no | yes | no |
| *IL6ST* | 5q11.2 | IL6 signal transducer (IL6ST) deficiency | AR | ID | PMID: 31953710 | no | yes | no |
| *IL7R* | 5p13.2 | Severe combined immunodeficiency, T-cell negative, B-cell/natural killer cell-positive type | AR | ID | PMID: 31953710 | no | yes | yes |
| *INO80* | 15q15.1 | INO80 deficiency | AR | ID | PMID: 31953710 | no | yes | no |
| *IRAK1* | Xq28 | IRAK1 deficiency | XL | ID | PMID: 31953710 | no | yes | yes |
| *IRAK4* | 12q12 | IRAK4 deficiency, Invasive pneumococcal disease, recurrent, isolated, 1 |  | ID | PMID: 31953710 | no | yes | no |
| *IRF2BP2* | 1q42.3 | Immunodeficiency, common variable, 14 | AD | ID | PMID: 31953710 | no | yes | no |
| *IRF3* | 19q13.33 | IRF3 deficiency | AD | ID | PMID: 31953710 | no | yes | no |
| *IRF4* | 6p25.3 | IRF4 haploinsufficiency | AD | ID | PMID: 31953710 | no | yes | yes |
| *IRF7* | 11p15.5 | IRF7 deficiency | AR | ID | PMID: 31953710 | no | yes | no |
| *IRF8* | 16q24.1 | Immunodeficiency 32A, mycobacteriosis, autosomal dominant, Immunodeficiency 32B, monocyte and dendritic cell deficiency, autosomal recessive | AD/AR | ID | PMID: 31953710 | no | yes | yes |
| *IRF9* | 14q12 | IRF9 deficiency | AR | ID | PMID: 31953710 | no | yes | no |
| *ISG15* | 1p36.33 | Immunodeficiency 38 | AR | ID | PMID: 31953710 | no | yes | no |
| *ITCH* | 20q11.22 | ITCH deficiency | AR | ID | PMID: 31953710 | no | yes | no |
| *ITGB2* | 21q22.3 | Leukocyte adhesion deficiency | AR | ID | PMID: 31953710 | no | yes | no |
| *ITK* | 5q33.3 | Lymphoproliferative syndrome 1 | AR | ID | PMID: 31953710 | no | yes | yes |
| *JAK1* | 1p31.3 | Primary immunodeficiency | AR | ID | PMID: 31953710 | no | yes | yes |
| *JAK3* | 19p13.11 | SCID, autosomal recessive, T-negative/B-positive type | AR | ID | PMID: 31953710 | no | yes | yes |
| *KDM6A* | Xp11.3 | Kabuki syndrome (type 1 and 2) | XL | ID | PMID: 31953710 | no | yes | yes |
| *KMT2A* | 11q23.3 | KMT2A deficiency | AD | ID | PMID: 31953710 | no | yes | yes |
| *KMT2D* | 12q13.12 | Kabuki syndrome (type 1 and 2) | AD | ID | PMID: 31953710 | no | yes | yes |
| *KRAS* | 12p12.1 | RAS-associated autoimmune leukoproliferative disorder | AD | ID | PMID: 16474405 | no | no | yes |
| *LAT* | 16q13 | Immunodeficiency 52 | AR | ID | PMID: 31953710 | no | yes | no |
| *LCK* | 1p35.2 | Immunodeficiency 22 | AR | ID | PMID: 31953710 | no | yes | no |
| *LIG1* | 19q13.33 | Ligase I deficiency | AR | ID | PMID: 31953710 | no | yes | no |
| *LPIN2* | 18p11.31 | Majeed syndrome | AR | ID | PMID: 31953710 | no | yes | no |
| *LRBA* | 4q31.3 | Immunodeficiency, common variable, 8, with autoimmunity | AR | ID | PMID: 31953710 | no | yes | no |
| *MAGT1* | Xq21.1 | Immunodeficiency, X-linked, with magnesium defect, Epstein-Barr virus infection and neoplasia | XR | ID | PMID: 31953710 | no | yes | yes |
| *MALT1* | 18q21.32 | Immunodeficiency 12 | AR | ID | PMID: 31953710 | no | yes | yes |
| *MAP3K14* | 17q21.31 | Primary immunodeficiency with multifaceted aberrant lymphoid immunity | AR | ID | PMID: 31953710 | no | yes | yes |
| *MASP1* | 3q27.3 | 3MC syndrome 1 | AR | ID | PMID: 26454309 | no | no | no |
| *MASP2* | 1p36.22 | MASP2 deficiency | AR | ID | PMID: 31953710 | no | yes | no |
| *MCM4* | 8q11.21 | MCM4 deficiency | AR | ID | PMID: 31953710 | no | yes | no |
| *MEFV* | 16p13.3 | Familial Mediterranean fever | AD/AR | ID | PMID: 31953710 | no | yes | no |
| *MOGS* | 2p13.1 | Congenital disorder of glycosylation, type IIb | AR | ID | PMID: 31953710 | no | yes | no |
| *MRTFA* | 22q13.1-q13.2 | Immunodeficiency 66 | AR | ID | PMID: 31953710 | no | yes | no |
| *MS4A1* | 11q12.2 | CD20 deficiency | AR | ID | PMID: 31953710 | no | yes | no |
| *MSH6* | 2p16.3 | MSH6 deficiency | AR | ID | PMID: 31953710 | no | yes | yes |
| *MSN* | Xq12 | Immunodeficiency 50 | XR | ID | PMID: 31953710 | no | yes | no |
| *MTHFD1* | 14q23.3 | Combined immunodeficiency and megaloblastic anemia with or without hyperhomocysteinemia | AR | ID | PMID: 31953710 | no | yes | no |
| *MVK* | 12q24.11 | Mevalonate kinase deficiency | AR | ID | PMID: 31953710 | no | yes | no |
| *MYD88* | 3p22.2 | Pyogenic bacterial infections, recurrent, due to MYD88 deficiency |  | ID | PMID: 31953710 | no | yes | yes |
| *MYO5A* | 15q21.2 | Griscelli syndrome, type 1 | AR | ID | Phenotype MIM number: 214450 | no | no | no |
| *MYSM1* | 1p32.1 | MYSM1 deficiency | AR | ID | PMID: 31953710 | no | yes | no |
| *NBAS* | 2p24.3 | Acute liver failure due to NBAS deficiency | AR | ID | PMID: 31953710 | no | yes | no |
| *NBN* | 8q21.3 | Nijmegen breakage syndrome, Non-Hodgkin lymphoma, Acute lymphoblastic leukemia (primarily T cell) | AR | ID | PMID: 31953710 | no | yes | yes |
| *NCF1* | 7q11.23 | Chronic granulomatous disease due to deficiency of NCF-1 | AR | ID | PMID: 31953710 | no | yes | no |
| *NCF2* | 1q25.3 | Chronic granulomatous disease due to deficiency of NCF-2 | AR | ID | PMID: 31953710 | no | yes | no |
| *NCF4* | 22q12.3 | Granulomatous disease, chronic, autosomal recessive, cytochrome b-positive, type III | AR | ID | PMID: 31953710 | no | yes | no |
| *NCSTN* | 1q23.2 | Acne inversa, familial 1 | AD | ID | PMID: 31953710 | no | yes | no |
| *NFAT5* | 16q22.1 | NFAT5 haploinsufficiency | AD | ID | PMID: 31953710 | no | yes | no |
| *NFE2L2* | 2q31.2 | Activating de novo mutations in nuclear factor, erythroid 2- like (NFE2L2) | AD | ID | PMID: 31953710 | no | yes | no |
| *NFKB1* | 4q24 | Immunodeficiency, common variable, 12 | AD | ID | PMID: 26279205 | no | no | no |
| *NFKB2* | 10q24.32 | Immunodeficiency, common variable, 10 | AD | ID | PMID: 31953710 | no | yes | yes |
| *NFKBIA* | 14q13.2 | Ectodermal dysplasia and immunodeficiency 2 | AD | ID | PMID: 31953710 | no | yes | yes |
| *NHEJ1* | 2q35 | Severe combined immunodeficiency with microcephaly, growth retardation, and sensitivity to ionizing radiation |  | ID | PMID: 31953710 | no | yes | yes |
| *NLRC4* | 2p22.3 | Autoinflammation with infantile enterocolitis | AD | ID | PMID: 31953710 | no | yes | no |
| *NLRP1* | 17p13.2 | Autoinflammation with arthritis and dyskeratosis | AD/AR | ID | PMID: 31953710 | no | yes | no |
| *NLRP12* | 19q13.42 | Familial cold autoinflammatory syndrome 2 | AD | ID | PMID: 31953710 | no | yes | no |
| *NLRP3* | 1q44 | Chronic infantile neurologic cutaneous articular (CINCA) syndrome | AD | ID | PMID: 31953710 | no | yes | no |
| *NOD2* | 16q12.1 | Blau syndrome | AD | ID | PMID: 31953710 | no | yes | no |
| *NSMCE3* | 15q13.1 | Lung disease, immunodeficiency, and chromosome breakage syndrome (LICS) | AR | ID | PMID: 31953710 | no | yes | no |
| *OAS1* | 12q24.13 | OAS1 deficiency | AD | ID | PMID: 31953710 | no | yes | no |
| *ORAI1* | 12q24.31 | Immunodeficiency 9 | AR | ID | PMID: 31953710 | no | yes | yes |
| *OSTM1* | 6q21 | Osteopetrosis | AR | ID | PMID: 31953710 | no | yes | no |
| *OTULIN* | 5p15.2 | Otulipenia/ORAS | AR | ID | PMID: 31953710 | no | yes | no |
| *PEPD* | 19q13.11 | Prolidase deficiency | AR | ID | PMID: 31953710 | no | yes | no |
| *PGM3* | 6q14.1 | Immunodeficiency 23 | AR | ID | PMID: 31953710 | no | yes | no |
| *PIK3CD* | 1p36.22 | Immunodeficiency 14 | AD | ID | PMID: 31953710 | no | yes | no |
| *PIK3R1* | 5q13.1 | Immunodeficiency 36, Agammaglobulinemia 7 | AD/AR | ID | PMID: 31953710 | no | yes | yes |
| *PLCG2* | 16q23.3 | Autoinflammation, antibody deficiency, and immune dysregulation syndrome | AD | ID | PMID: 31953710 | no | yes | yes |
| *PLEKHM1* | 17q21.31 | Osteopetrosis | AR | ID | PMID: 31953710 | no | yes | no |
| *PMS2* | 7p22.1 | PMS2 deficiency | AR | ID | PMID: 31953710 | no | yes | yes |
| *PNP* | 14q11.2 | Immunodeficiency due to purine nucleoside phosphorylase deficiency | AR | ID | PMID: 31953710 | no | yes | yes |
| *POLA1* | Xp22.11-p21.3 | X-linked reticulate pigmentary disorder | XL | ID | PMID: 31953710 | no | yes | no |
| *POLD1* | 19q13.3 | Polymerase and deficiency | AR | ID | PMID: 31953710 | no | yes | no |
| *POLD2* | 7p13 | Polymerase and deficiency | AR | ID | PMID: 31953710 | no | yes | no |
| *POLE* | 12q24.33 | FILS syndrome, IMAGE-I syndrome | AR | ID | PMID: 31953710 | no | yes | no |
| *POLE2* | 14q21.3 | Combined immunodeficiency | AR | ID | PMID: 31953710 | no | yes | no |
| *POLR3A* | 10q22.3 | RNA polymerase III deficiency | AD | ID | PMID: 31953710 | no | yes | no |
| *POLR3C* | 1q21.1 | RNA polymerase III deficiency | AD | ID | PMID: 31953710 | no | yes | no |
| *POLR3F* | 20p11.23 | RNA polymerase III deficiency | AD | ID | PMID: 31953710 | no | yes | no |
| *PRF1* | 10q22.1 | Hemophagocytic lymphohistiocytosis, familial, 2 | AR | ID | PMID: 31953710 | no | yes | yes |
| *PRKCD* | 3p21.1 | Autoimmune lymphoproliferative syndrome type III | AR | ID | PMID: 31953710 | no | yes | no |
| *PRKDC* | 8q11.21 | Immunodeficiency 26, with or without neurologic abnormalities | AR | ID | PMID: 31953710 | no | yes | yes |
| *PSEN1* | 14q24.2 | Hidradenitis suppurativa | AD | ID | PMID: 31953710 | no | yes | no |
| *PSENEN* | 19q13.12 | Hidradenitis suppurativa | AD | ID | PMID: 31953710 | no | yes | no |
| *PSMB8* | 6p21.32 | Proteasome-associated autoinflammatory syndrome 1 and digenic forms | AR | ID | PMID: 31953710 | no | yes | no |
| *PSMG2* | 18p11.21 | CANDLE (chronic atypical neutrophilic dermatitis with lipodystrophy) | AR | ID | PMID: 31953710 | no | yes | no |
| *PSTPIP1* | 15q24.3 | Pyogenic sterile arthritis, pyoderma gangrenosum, and acne | AD | ID | PMID: 31953710 | no | yes | no |
| *PTEN* | 10q23.31 | PTEN deficiency (LOF) | AD | ID | PMID: 31953710 | no | yes | yes |
| *PTPRC* | 1q31.3-q32.1 | Severe combined immunodeficiency, T cell-negative, B-cell/natural killer-cell positive | AR | ID | PMID: 31953710 | no | yes | yes |
| *RAC2* | 22q13.1 | Neutrophil immunodeficiency syndrome | AD | ID | PMID: 31953710 | no | yes | yes |
| *RAG1* | 11p12 | Omenn syndrome, Alpha/beta T-cell lymphopenia with gamma/delta T-cell expansion, severe cytomegalovirus infection, and autoimmunity, Severe combined immunodeficiency, B cell-negative, Combined cellular and humoral immune defects with granulomas | AR | ID | PMID: 31953710 | no | yes | yes |
| *RAG2* | 11p12 | Omenn syndrome, Combined cellular and humoral immune defects with granulomas, Severe combined immunodeficiency, B cell-negative | AR | ID | PMID: 31953710 | no | yes | yes |
| *RANBP2* | 2q13 | Acute necrotizing encephalopathy | AR | ID | PMID: 31953710 | no | yes | no |
| *RASGRP1* | 15q14 | Immunodeficiency 64 | AR | ID | PMID: 31953710 | no | yes | no |
| *RBCK1* | 20p13 | Polyglucosan body myopathy 1 with or without immunodeficiency | AR | ID | PMID: 31953710 | no | yes | no |
| *RECQL4* | 8q24.3 | Baller-Gerold syndrome, RAPADILINO syndrome, Rothmund-Thomson syndrome | AR | ID | PMID: 18716613 | no | no | no |
| *REL* | 2p16.1 | c-Rel deficiency | AR | ID | PMID: 31953710 | no | yes | yes |
| *RELA* | 11q13.1 | RelA haploinsufficiency | AD | ID | PMID: 31953710 | no | yes | no |
| *RELB* | 19q13.32 | RelB deficiency | AR | ID | PMID: 31953710 | no | yes | no |
| *RFX5* | 1q21.3 | Bare lymphocyte syndrome, type II, complementation group C, E | AR | ID | PMID: 31953710 | no | yes | no |
| *RFXANK* | 19p13.11 | MHC class II deficiency, complementation group B | AR | ID | PMID: 31953710 | no | yes | no |
| *RFXAP* | 13q13.3 | Bare lymphocyte syndrome, type II, complementation group D | AR | ID | PMID: 31953710 | no | yes | yes |
| *RHOH* | 4p14 | Epidermodysplasia verruciformis, susceptibility to, 4 | AR | ID | PMID: 31953710 | no | yes | no |
| *RIPK1* | 6p25.2 | RIPK1 | AR | ID | PMID: 31953710 | no | yes | yes |
| *RNASEH2A* | 19p13.13 | Aicardi-Goutieres syndrome 4 | AR | ID | PMID: 31953710 | no | yes | no |
| *RNASEH2B* | 13q14.3 | Aicardi-Goutieres syndrome 2 | AR | ID | PMID: 31953710 | no | yes | no |
| *RNASEH2C* | 11q13.1 | Aicardi-Goutieres syndrome 3 | AR | ID | PMID: 31953710 | no | yes | no |
| *RNF168* | 3q29 | RIDDLE syndrome | AR | ID | PMID: 31953710 | no | yes | no |
| *RNF31* | 14q12 | HOIP and LUBAC deficiency | AR | ID | PMID: 31953710 | no | yes | no |
| *RNU4ATAC* | 2q14.2 | Roifman syndrome | AR | ID | PMID: 31953710 | no | yes | no |
| *RORC* | 1q21.3 | Immunodeficiency 42 | AR | ID | PMID: 31953710 | no | yes | no |
| *RPSA* | 3p22.1 | Asplenia, isolated congenital | AD | ID | PMID: 31953710 | no | yes | no |
| *SAMHD1* | 20q11.23 | Chilblain lupus 2, Aicardi-Goutières syndrome | AD/AR | ID | PMID: 31953710 | no | yes | yes |
| *SEC61A1* | 3q21.3 | SEC61A1 deficiency | AD | ID | PMID: 31953710 | no | yes | no |
| *SEMA3E* | 7q21.11 | CHARGE syndrome | AD | ID | PMID: 31953710 | no | yes | no |
| *SERPING1* | 11q12.1 | Complement component 4, partial deficiency of, Angioedema, hereditary, types I and II | AD/AR | ID | PMID: 31953710 | no | yes | no |
| *SH2D1A* | Xq25 | Lymphoproliferative syndrome | XR | ID | PMID: 31953710 | no | yes | yes |
| *SH3BP2* | 4p16.3 | Cherubism | AD | ID | PMID: 31953710 | no | yes | no |
| *SH3KBP1* | Xp22.12 | SH3KBP1 (CIN85) deficiency | XL | ID | PMID: 31953710 | no | yes | no |
| *SKIV2L* | 6p21.33 | Tricho-Hepato-Enteric Syndrome (THES) | AR | ID | PMID: 31953710 | no | yes | no |
| *SLC29A3* | 10q22.1 | Histiocytosis-lymphadenopathy plus syndrome | AR | ID | PMID: 31953710 | no | yes | no |
| *SLC35C1* | 11p11.2 | Congenital disorder of glycosylation, type IIc | AR | ID | PMID: 31953710 | no | yes | no |
| *SLC39A7* | 6p21.32 | SLC39A7 (ZIP7) deficiency | AR | ID | PMID: 31953710 | no | yes | no |
| *SLC46A1* | 17q11.2 | Folate malabsorption, hereditary | AR | ID | PMID: 31953710 | no | yes | no |
| *SLC7A7* | 14q11.2 | Lysinuric protein intolerance | AR | ID | PMID: 31953710 | no | yes | yes |
| *SMARCAL1* | 2q35 | Schimke immunoosseous dysplasia | AR | ID | PMID: 31953710 | no | yes | no |
| *SMARCD2* | 17q23.3 | Specific granule defiency 2 | AR | ID | PMID: 31953710 | no | yes | no |
| *SNX10* | 7p15.2 | Osteopetrosis | AR | ID | PMID: 31953710 | no | yes | no |
| *SP110* | 2q37.1 | Hepatic venoocclusive disease with immunodeficiency | AR | ID | PMID: 31953710 | no | yes | no |
| *SPINK5* | 5q32 | Netherton syndrome | AR | ID | PMID: 31953710 | no | yes | no |
| *SPPL2A* | 15q21.2 | SPPL2a deficiency | AR | ID | PMID: 31953710 | no | yes | no |
| *STAT1* | 2q32.2 | Immunodeficiency 31A, 31B, 31C | AD/AR | ID | PMID: 31953710 | no | yes | no |
| *STAT2* | 12q13.3 | Immunodeficiency 44, Pseudo-TORCH syndrome 3 | AR | ID | PMID: 31953710 | no | yes | no |
| *STAT3* | 17q21.2 | Hyper-IgE recurrent infection syndrome, Autoimmune disease, multisystem, infantile onset | AD | ID | PMID: 31953710 | no | yes | yes |
| *STAT5B* | 17q21.2 | Growth hormone insensitivity with immunodeficiency |  | ID | PMID: 31953710 | no | yes | yes |
| *STIM1* | 11p15.4 | Stormorken syndrome, Immunodeficiency 10 | AD/AR | ID | PMID: 31953710 | no | yes | yes |
| *STING1* | 5q31.2 | STING-associated vasculopathy, infantile-onsent (SAVI) | AD | ID | PMID: 31953710 | no | yes | no |
| *STK4* | 20q13.12 | T-cell immunodeficiency, recurrent infections, autoimmunity, and cardiac malformations |  | ID | PMID: 31953710 | no | yes | no |
| *STX11* | 6q24.2 | Hemophagocytic lymphohistiocytosis, familial, 4 | AR | ID | PMID: 31953710 | no | yes | yes |
| *STXBP2* | 19p13.2 | Hemophagocytic lymphohistiocytosis, familial, 5 | AR | ID | PMID: 31953710 | no | yes | yes |
| *TAP1* | 6p21.32 | Bare lymphocyte syndrome, type I | AR | ID | PMID: 31953710 | no | yes | no |
| *TAP2* | 6p21.32 | Bare lymphocyte syndrome, type I, due to TAP2 deficiency | AR | ID | PMID: 31953710 | no | yes | no |
| *TAPBP* | 6p21.32 | Bare lymphocyte syndrome, type I | AR | ID | PMID: 31953710 | no | yes | no |
| *TBK1* | 12q14.2 | TBK1 deficiency | AD | ID | PMID: 31953710 | no | yes | no |
| *TBX1* | 22q11.21 | DiGeorge syndrome, Velocardiofacial syndrome | AD | ID | PMID: 31953710 | no | yes | yes |
| *TCF3* | 19p13.3 | Agammaglobulinemia 8, autosomal dominant | AD | ID | PMID: 31953710 | no | yes | no |
| *TFRC* | 3q29 | Immunodeficiency 46 | AR | ID | PMID: 31953710 | no | yes | no |
| *TGFBR1* | 9q22.33 | Loeys-Dietz syndrome (TGFBR deficiency) | AD | ID | PMID: 31953710 | no | yes | no |
| *TGFBR2* | 3p24.1 | Loeys-Dietz syndrome (TGFBR deficiency) | AD | ID | PMID: 31953710 | no | yes | no |
| *THBD* | 20p11.21 | Thrombomodulin deficiency | AD | ID | PMID: 31953710 | no | yes | no |
| *TICAM1* | 19p13.3 | TRIF deficiency | AD | ID | PMID: 31953710 | no | yes | no |
| *TIRAP* | 11q24.2 | TIRAP deficiency | AR | ID | PMID: 31953710 | no | yes | no |
| *TLR3* | 4q35.1 | TLR3 deficiency | AD/AR | ID | PMID: 31953710 | no | yes | no |
| *TMC6* | 17q25.3 | Epidermodysplasia verruciformis | AR | ID | PMID: 31953710 | no | yes | no |
| *TMC8* | 17q25.3 | Epidermodysplasia verruciformis | AR | ID | PMID: 31953710 | no | yes | no |
| *TNFAIP3* | 6q23.3 | Autoinflammatory syndrome, familial, Behcet-like | AD | ID | PMID: 31953710 | no | yes | yes |
| *TNFRSF11A* | 18q21.33 | Osteopetrosis | AR | ID | PMID: 31953710 | no | yes | no |
| *TNFRSF13B* | 17p11.2 | Immunodeficiency, common variable, 2, Immunoglobulin A deficiency 2 | AD/AR | ID | PMID: 31953710 | no | yes | no |
| *TNFRSF13C* | 22q13.2 | BAFF receptor deficiency | AR | ID | PMID: 31953710 | no | yes | no |
| *TNFRSF1A* | 12p13.31 | Periodic fever, familial | AD | ID | Phenotype MIM number: 142680 | no | no | no |
| *TNFRSF1A* | 12p13.31 | TNF receptor-associated periodic syndrome (TRAPS) | AD | ID | PMID: 31953710 | no | yes | no |
| *TNFRSF4* | 1p36.33 | Immunodeficiency 16 | AR | ID | PMID: 31953710 | no | yes | no |
| *TNFRSF9* | 1p36.23 | CD137 deficiency | AR | ID | PMID: 31953710 | no | yes | no |
| *TNFSF11* | 13q14 | Osteopetrosis | AR | ID | PMID: 31953710 | no | yes | no |
| *TNFSF12* | 17p13.1 | TWEAK deficiency | AD | ID | PMID: 31953710 | no | yes | no |
| *TOP2B* | 3p24.2 | Hoffman syndrome/TOP2B deficiency | AD | ID | PMID: 31953710 | no | yes | no |
| *TPP2* | 13q33.1 | Tripeptidyl-peptidase II deficiency | AR | ID | PMID: 31953710 | no | yes | no |
| *TRAC* | 14q11.2 | TCRa deficiency | AR | ID | PMID: 31953710 | no | yes | no |
| *TRAF3* | 14q32.32 | TRAF3 deficiency | AD | ID | PMID: 31953710 | no | yes | yes |
| *TRAF3IP2* | 6q21 | Candidiasis, familial, 8 | AR | ID | PMID: 31953710 | no | yes | no |
| *TREX1* | 3p21.31 | Aicardi-Goutieres syndrome 1, dominant and recessive | AD/AR | ID | PMID: 31953710 | no | yes | no |
| *TRIM22* | 11p15.4 | TRIM22 | AR | ID | PMID: 31953710 | no | yes | no |
| *TRNT1* | 3p26.2 | Sideroblastic anemia with B-cell immunodeficiency, periodic fevers, and developmental delay | AR | ID | PMID: 31953710 | no | yes | no |
| *TTC37* | 5q15 | Tricho-Hepato-Enteric Syndrome (THES) | AR | ID | PMID: 31953710 | no | yes | no |
| *TTC7A* | 2p21 | Gastrointestinal defects and immunodeficiency syndrome | AR | ID | PMID: 31953710 | no | yes | no |
| *TYK2* | 19p13.2 | Immunodeficiency 35 | AR | ID | PMID: 31953710 | no | yes | yes |
| *UNC119* | 17q11.2 | Immunodeficiency 13 | AR | ID | PMID: 22184408 | no | no | no |
| *UNC13D* | 17q25.1 | Hemophagocytic lymphohistiocytosis, familial, 3 | AR | ID | PMID: 31953710 | no | yes | yes |
| *UNC93B1* | 11q13.2 | Encephalopathy, acute, infection-induced (herpes-specific), susceptibility to, 1 |  | ID | PMID: 31953710 | no | yes | no |
| *UNG* | 12q24.11 | Immunodeficiency with hyper-IgM, type 5 | AR | ID | PMID: 31953710 | no | yes | no |
| *USP18* | 22q11.21 | Pseudo-TORCH syndrome 2 | AR | ID | PMID: 31953710 | no | yes | no |
| *WDR1* | 4p16.1 | Immunodeficiency/autoinflammatory syndrome with aberrant morphology and function of myeloid cells |  | ID | PMID: 31953710 | no | yes | no |
| *WIPF1* | 2q31.1 | Wiskott-Aldrich syndrome 2 |  | ID | PMID: 31953710 | no | yes | yes |
| *XIAP* | Xq25 | Lymphoproliferative syndrome, X-linked, 2 | XR | ID | PMID: 31953710 | no | yes | yes |
| *ZAP70* | 2q11.2 | Immunodeficiency 48, Autoimmune disease, multisystem, infantile-onset, 2 | AR | ID | PMID: 31953710 | no | yes | yes |
| *ZBTB24* | 6q21 | Immunodeficiency-centromeric instability-facial anomalies syndrome 2 | AR | ID | PMID: 31953710 | no | yes | no |
| *ZNF341* | 20q11.22 | Hyper-IgE recurrent infection syndrome 3, autosomal recessive | AR | ID | PMID: 31953710 | no | yes | no |
| *AKT1* | 14q32.33 | Cowden syndrome 6 |  | Cancer predisposition | Phenotype MIM number: 615109 | no | no | no |
| *ANKRD26* | 10p12.1 | ANKRD26-related thrombocytopenia | AD | Cancer predisposition | PMID: 29927566 | yes | no | yes |
| *APC* | 5q22.2 | Familial adenomatous polyposis | AD | Cancer predisposition | PMID: 11135435 | no | no | yes |
| *ASXL1* | 20q11.1 | Bohring-Opitz syndrome | AD | Cancer predisposition | PMID: 21706002 | no | no | yes |
| *ATG2B* | 14q32.2 | Predisposition to familial myeloid malignancie |  | Cancer predisposition | PMID: 27308616 | no | no | no |
| *ATR* | 3q23 | Cutaneous telangiectasia and cancer syndrome | AD | Cancer predisposition | PMID: 22341969 | no | no | yes |
| *BAP1* | 3p21.1 | Tumor predisposition syndrome | AD | Cancer predisposition | Phenotype MIM number: 614327 | no | no | no |
| *BARD1* | 2q35 | Familial cancer of breast | AD | Cancer predisposition | PMID: 15342711 | no | no | yes |
| *BMPR1A* | 10q23.2 | Juvenile polyposis syndrome | AD | Cancer predisposition | PMID: 11381269 | no | no | yes |
| *CBL* | 11q23.3 | Juvenile myelomonocytic leukemia, Noonan syndrome-like disorder with or without juvenile myelomonocytic leukemia | AD | Cancer predisposition | Phenotype MIM number: 607785 | no | no | yes |
| *CDC73* | 1q31.2 | Hyperparathyroidism-jaw tumor syndrome | AD | Cancer predisposition | PMID: 12434154 | no | no | yes |
| *CDH1* | 16q22.1 | Hereditary diffuse gastric cancer, Familial cancer of breast | AD | Cancer predisposition | 9537325, 17660459 | no | no | yes |
| *CDK4* | 12q14.1 | Melanoma, cutaneous malignant, 3 | AD | Cancer predisposition | PMID: 21051013 | no | no | no |
| *CDKN2A* | 9p21.3 | Pancreatic cancer/melanoma syndrome | AD | Cancer predisposition | PMID: 7666917 | no | no | yes |
| *CEBPA* | 19q13.1 | CEBPA-Associated Familial Acute Myeloid Leukemia | AD | Cancer predisposition | PMID: 15575056 | yes | no | yes |
| *CHEK2* | 22q12.1 | Li-Fraumeni syndrome | AD | Cancer predisposition | PMID: 10617473 | no | no | yes |
| *CREBBP* | 16p13.3 | Rubinstein-Taybi syndrome | AD | Cancer predisposition | PMID: 9294190 | no | no | yes |
| *DDX41* | 5q35.3 | Familial myeloproliferative/lymphoproliferative neoplasms | AD | Cancer predisposition | PMID: 25920683 | yes | no | yes |
| *DICER1* | 14q32.13 | Pleuropulmonary blastoma, Rhabdomyosarcoma, embryonal, 2 | AD | Cancer predisposition | Phenotype MIM number: 180295 | no | no | no |
| *EP300* | 22q13.2 | Rubinstein-Taybi syndrome | AD | Cancer predisposition | PMID: 15706485 | no | no | yes |
| *EPCAM* | 2p21 | Colorectal cancer, hereditary nonpolyposis, type 8 |  | Cancer predisposition | Phenotype MIM number: 613244 | no | no | no |
| *ERBB3* | 12q13.2 | Erythroleukemia, familial, susceptibility to | AD | Cancer predisposition | Phenotype MIM number: 133180 | no | no | no |
| *ERCC6* | 10q11.23 | Lung cancer, susceptibility to | AD | Cancer predisposition | Phenotype MIM number: 211980 | no | no | yes |
| *ETV6* | 12p13.2 | Thrombocytopenia 5 | AD | Cancer predisposition | PMID: 25581430 | yes | no | yes |
| *EZH2* | 7q36.1 | Weaver syndrome | AD | Cancer predisposition | PMID: 22177091 | no | no | yes |
| *FH* | 1q43 | Leiomyomatosis and renal cell cancer | AD | Cancer predisposition | Phenotype MIM number: 150800 | no | no | no |
| *FLCN* | 17p11.2 | Birt-Hogg-Dube syndrome | AD | Cancer predisposition | PMID: 28970150 | no | no | no |
| *GALNT12* | 9q22.33 | Colorectal cancer, susceptibility to, 1 |  | Cancer predisposition | Phenotype MIM number: 608812 | no | no | no |
| *GREM1* | 15q13.3 | Predisposition to colorectal cancer |  | Cancer predisposition | PMID: 30584801 | no | no | no |
| *GSKIP* | 14q32.2 | Predisposition to familial myeloid malignancie |  | Cancer predisposition | PMID: 27308616 | no | no | no |
| *HLTF* | 3q24 | DNA damage accumulation in familial MDS |  | Cancer predisposition | PMID: 30696947 | no | no | no |
| *HOXB13* | 17q21.32 | Prostate cancer, hereditary, 9 |  | Cancer predisposition | Phenotype MIM number: 6610997 | no | no | no |
| *HRAS* | 11p15.5 | Costello syndrome | AD | Cancer predisposition | PMID: 16170316 | no | no | yes |
| *KDM1A* | 1p36.12 | Susceptibility to multiple myeloma |  | Cancer predisposition | PMID: 29559475 | no | no | no |
| *KIT* | 4q12 | Gastrointestinal stromal tumor, familial |  | Cancer predisposition | Phenotype MIM number: 606764 | no | no | yes |
| *LAPTM5* | 1p35.2 | Familial Waldenström macroglobulinemia |  | Cancer predisposition | PMID: 26903547 | no | no | no |
| *MAX* | 14q23.3 | Pheochromocytoma, susceptibility to | AD | Cancer predisposition | Phenotype MIM number: 171300 | no | no | no |
| *MBD4* | 3q21.3 | Predisposition to uveal melanoma |  | Cancer predisposition | PMID: 32239153 | no | no | no |
| *MC1R* | 16q24.3 | Melanoma, cutaneous malignant, 5 |  | Cancer predisposition | Phenotype MIM number: 613099 | no | no | no |
| *MEN1* | 11q13.1 | Multiple endocrine neoplasia 1 | AD | Cancer predisposition | PMID: 25099597 | no | no | no |
| *MET* | 7q31.2 | Papillary renal cell carcinoma |  | Cancer predisposition | PMID: 9140397 | no | no | yes |
| *MITF* | 3p13 | Melanoma, cutaneous malignant, susceptibility to, 8 |  | Cancer predisposition | Phenotype MIM number: 614456 | no | no | no |
| *MLH1* | 3p22.2 | Hereditary non-polyposis colon cancer |  | Cancer predisposition | PMID: 7903889 | no | no | yes |
| *MSH2* | 2p21-p16 | Hereditary nonpolyposis colon cancer, type1 | AD | Cancer predisposition | PMID: 8252616 | no | no | yes |
| *MST1R* | 3p21.31 | Nasopharyngeal carcinoma, susceptibility to, 3 | AD | Cancer predisposition | Phenotype MIM number: 617075 | no | no | yes |
| *MUTYH* | 1p34.1 | Familial adenomatous polyposis 2 | AR | Cancer predisposition | PMID: 12393807 | no | no | yes |
| *NF1* | 17q11.2 | Neurofibromatosis, type 1, Juvenile myelomonocytic leukemia | AD | Cancer predisposition | PMID: 9639526 | no | no | yes |
| *NF2* | 22q12.2 | Neurofibromatosis, type 2, predisposition to central and peripheral nervous system tumors (meningiomas, schwannomas, ependymomas), subcutaneous tumors | AD | Cancer predisposition | Phenotype MIM number: 101000 | no | no | no |
| *PAX5* | 9p13.2 | B-cell acute lymphoblastic leukemia-3 |  | Cancer predisposition | PMID: 24013638 | no | no | yes |
| *PDGFRA* | 4q12 | Gastrointestinal stromal tumor/GIST-plus syndrome, somatic or familial |  | Cancer predisposition | Phenotype MIM number: 175510 | no | no | yes |
| *PIK3CA* | 3q26.32 | Cowden syndrome 5 |  | Cancer predisposition | Phenotype MIM number: 615108 | no | no | no |
| *POT1* | 7q31.33 | Glioma susceptibility 9, Melanoma, cutaneous malignant, susceptibility to, 10 | AD | Cancer predisposition | Phenotype MIM number: 616568 | no | no | yes |
| *PTCH1* | 9q22.32 | Basal cell nevus syndrome, Holoprosencephaly 7 | AD | Cancer predisposition | Phenotype MIM number: 109400 | no | no | no |
| *PTPN11* | 12q24.13 | Noonan syndrome | AD | Cancer predisposition | PMID: 11704759 | no | no | yes |
| *RAD51D* | 17q12 | Breast-ovarian cancer, familial, susceptibility to, 4 |  | Cancer predisposition | Phenotype MIM number: 614291 | no | no | no |
| *RB1* | 13q14.2 | Retinoblastoma | AD | Cancer predisposition | PMID: 2895471 | no | no | yes |
| *RBBP6* | 16p12.1 | Predisposition to myeloproliferative neoplasms | ? | Cancer predisposition | PMID: 26574608 | no | no | no |
| *RET* | 10q11.21 | Medullary thyroid carcinoma, Multiple endocrine neoplasia IIA, IIB, Pheochromocytoma | AD | Cancer predisposition | Phenotype MIM number: 155240 | no | no | no |
| *RUNX1* | 21q22.12 | Familial platelet disorder with associated myeloid malignancy | AD | Cancer predisposition | PMID: 11830488 | yes | no | yes |
| *SMARCA4* | 19p13.2 | rhabdoid tumor predisposition syndrome-2 | AD | Cancer predisposition | Phenotype MIM number: 613325 | no | no | no |
| *SDHA* | 5p15.33 | Paragangliomas 5 | AD | Cancer predisposition | Phenotype MIM number: 614165 | no | no | no |
| *SDHAF2* | 11q12.2 | Paragangliomas 2 | AD | Cancer predisposition | Phenotype MIM number: 601650 | no | no | no |
| *SDHB* | 1p36.13 | Gastrointestinal stromal tumor, Paragangliomas 4, Pheochromocytoma | AD | Cancer predisposition | Phenotype MIM number: 115310 | no | no | no |
| *SDHC* | 1q23.3 | Gastrointestinal stromal tumor, Paragangliomas 3 | AD | Cancer predisposition | Phenotype MIM number: 605373 | no | no | no |
| *SDHD* | 11q23.1 | Paragangliomas 1, with or without deafness, Pheochromocytoma | AD | Cancer predisposition | Phenotype MIM number: 171300 | no | no | no |
| *SETBP1* | 18q12.3 | Schinzel-Giedion midface retraction syndrome | AD | Cancer predisposition | PMID: 20436468 | no | no | yes |
| *SH2B3* | 12q24.12 | Predisposition to acute lymphoblastic leukemia |  | Cancer predisposition | PMID: 23908464 | no | no | yes |
| *SMAD4* | 18q21.2 | Juvenile polyposis syndrome | AD | Cancer predisposition | PMID: 9545410 | no | no | yes |
| *SMARCA4* | 19p13.2 | Rhabdoid tumor predisposition syndrome 2 | AD | Cancer predisposition | PMID: 20137775 | no | no | yes |
| *SMARCB1* | 22q11.23 | Rhabdoid tumor predisposition syndrome 1 | AD | Cancer predisposition | PMID: 10521299 | no | no | yes |
| *SOS1* | 2p22.1 | Noonan syndrome 4 | AD | Cancer predisposition | PMID: 610733 | no | no | yes |
| *STK11* | 19p13.3 | Peutz-Jeghers syndrome | AD | Cancer predisposition | Phenotype MIM number: 175200 | no | no | yes |
| *TERF2IP* | 16q23.1 | Familial chronic lymphocytic leukemia |  | Cancer predisposition | PMID: 27528712 | no | no | no |
| *TMEM127* | 2q11.2 | Pheochromocytoma, susceptibility to | AD | Cancer predisposition | Phenotype MIM number: 171300 | no | no | no |
| *VHL* | 3p25.3 | von Hippel-Lindau syndrome | AD | Cancer predisposition | PMID: 8493574 | no | no | yes |
| *WRN* | 8p12 | Werner syndrome, Exocrine pancreatic cancer | AR | Cancer predisposition | PMID: 20657174 | no | no | no |
| *WT1* | 11p13 | Wilms tumor, type 1 | AD | Cancer predisposition | PMID: 15150775 | no | no | yes |

^*^Genes related to CN or IBMF introduced by WHO (WHO Classification of Tumours of Haematopoietic and Lymphoid Tissues, Revised 4^th^ Edition)

^†^Immunodeficiency-related genes reported by IUIS (J Clin Immunol. 2020 Jan;40(1):24-64)

^‡^Targeted sequencing using an in-house panel of 507 genes was performed on one patient (P-08).

Abbreviations: AD, autosomal dominant; AR, autosomal recessive; IBMF, inherited bone marrow failure; IUIS, International Union of Immunological Societies; WHO, World Health Organization; XR, X-linked recessive.
